# Synergistic Tuning of Diffusion Enables Surface-Passivation-Directed Electrodeposition of Shaped Bimetallic Nanoparticles

**DOI:** 10.1021/acsnano.6c13712

**Published:** 2026-10-25

**Authors:** Gabriel C. Halford, Abigail M. Sublett, Adam J. Buratowski, Kaelyn M. Harmon, Harikrishnan N. Nambiar, Michelle L. Personick

**Affiliations:** † Department of Chemistry, 2358 University of Virginia, Charlottesville, Virginia 22904, United States; ‡ Department of Materials Science and Engineering, University of Virginia, Charlottesville, Virginia 22904, United States

**Keywords:** electrodeposition, metal nanoparticles, nanoparticle synthesis, shape control, gold, silver, bimetallic

## Abstract

Many applications for shape-controlled metal nanoparticles require their immobilization on a surface, creating a need for development of direct substrate-based synthesis routes for increasingly complex materials. Colloidal chemical syntheses for shape-controlled nanoparticles are common, but transferring insight from synthesis in solution to growth on a substrate requires accounting for significant differences in reagent diffusion between these two growth environments—a nontrivial challenge. In the present work, we establish fundamental principles for rationally adapting reaction parameters from colloidal syntheses to electrodeposition such that the growth of nanoparticles on the electrode proceeds under comparable reagent diffusion conditions to those that drive shape direction during colloidal growth. To demonstrate these principles, we successfully translate reaction conditions from colloidal synthesis to electrodeposition for a series of dilute bimetallic (silver) gold nanoparticles whose shapes are controlled by surface deposition of silver: concave cubes, octahedra, rhombic dodecahedra, truncated ditetragonal prisms, and tetrahexahedra. By comparing open-circuit potential measurements of the colloidal syntheses with chronopotentiometry measurements from galvanostatic electrodeposition, we determine the adjustments required to establish ideal growth kinetics for each electrodeposition. We identify specific synthetic handles—mode of mass transport, surfactant concentration, applied current density, and secondary metal concentration—required to modulate reagent transport to the electrode surface, as well as the direction they must be modified so that the relative rates of silver and gold ion reduction mimic colloidal growth. These generalizable synthetic parameters for controlling reagent diffusion and concentrations near the electrode surface open possibilities for the electrodeposition of complex multimetallic and shaped nanomaterials.

## Graphical abstract

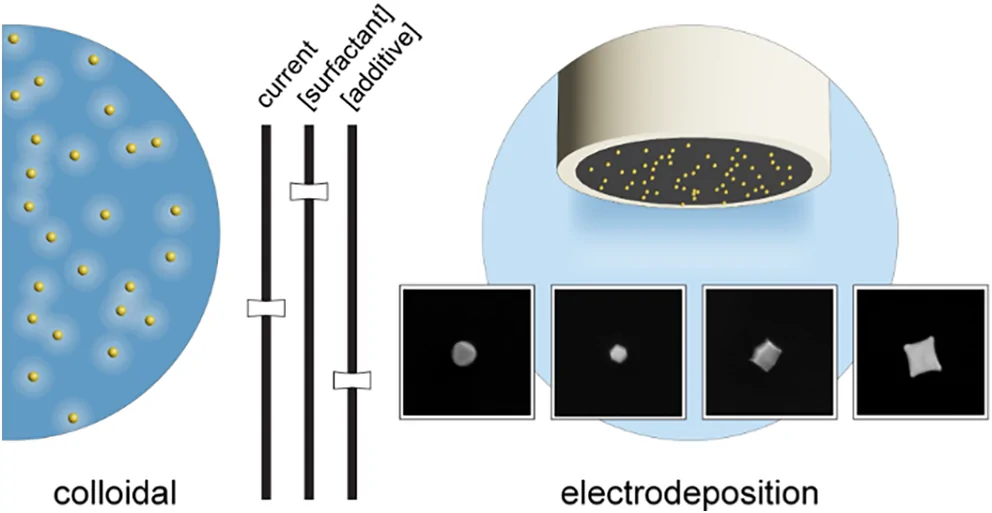

Shape-controlled metal nanomaterials are of broad interest for applications such as sensing^1,2^ and heterogeneous catalysis.^3–5^ Many implementations of these applications require well-defined nanoparticles to be immobilized on substrates, including electrode surfaces.^4,6^ Shaped metal nanoparticles are most commonly prepared through solution-phase (colloidal) synthesis with a chemical reducing agent and then processed onto a surface.^7,8^ This need for multiple processing steps to immobilize colloidal nanoparticles on a surface increases the complexity of functional substrate fabrication and can also introduce sample-to-sample inconsistencies, subsequently leading to irreproducible performance.^9^ Conversely, using established colloidal chemical methods to grow shaped nanoparticles directly on substrates is nontrivial and often unsuccessful, though a few recent successes have been reported.^10–14^ A rich literature of both templated and template-free electrochemical synthesis (electrodeposition) also exists for certain shaped nanomaterials, particularly nanorods, but it is more limited in scope of shape than what is accessible colloidally, and, with limited exceptions, templated syntheses generally yield polycrystalline materials as well as shapes with rounded edges and corners.^15–20^ Many of the central challenges in transferring established colloidal chemical methods to shaped nanoparticle growth on a surface arise from the significant differences in reagent diffusion for suspended nanoparticles vs those immobilized on surfaces.^21,22^ In colloidal chemical synthesis, particles undergo Brownian motion and readily access reagents via passive diffusion, an important process for some shape control mechanisms,^21^ while the movement of particles adsorbed to an electrode or other support is restricted.^22^ Reagents therefore must diffuse to the electrode or support surface directionally, and reagent access to particles is additionally blocked from one direction by the support. To better understand and address the gap between colloidal and supported nanoparticle growth, analytical methods that probe these dynamic processes during growth are required.

Our group has pioneered the use of open-circuit potential (OCP) measurements of the mixed solution potential during colloidal metal nanoparticle synthesis as a method for obtaining real-time information about growth chemistry, including the role of diffusion in driving shape formation.^21,23–25^ These mixed potential transients from OCP measurements of colloidal nanoparticle growth can be readily compared to real-time measurements of electrodeposition syntheses to inform the selection of electrodeposition conditions. We have used this approach to directly translate colloidal syntheses of monometallic Pd nanoparticles, including cubes, octahedra, and tetrahexahedra (THH), to electrodeposition by applying current waveforms designed to generate potential transients that mimic OCP measurements of the original colloidal syntheses.^23,24^ While controlled-potential deposition is more common in the electrodeposition literature because the potential can be set equal to the redox potential of metal ions in solution, minimizing the possibility of some side reactions,^18,19^ our group and others have had success with controlled-current approaches for the precise control of nanoparticle shape.^23,24,26,27^ We propose that the success of applied current approaches is a result of the modulation of the working electrode potential over time in response to the changing flux of reagent molecules near the working electrode, which mimics the crucial role of reagent diffusion in setting and refining growth rates of colloidal nanoparticles.^21^ The use of applied current is additionally powerful for translation of syntheses from colloidal growth, because it enables real-time measurement of the resulting potential during electrodeposition (chronopotentiometry), which can be compared to OCP measurements of colloidal synthesis to extract more specific and detailed fundamental understanding of the roles of metal ion diffusion and reaction kinetics during the electrodeposition process.^21,23–26^

This translation of colloidal growth to electrodeposition for Pd nanoparticles has generally required minor empirical adjustment of specific chemical parameters, such the concentrations of the metal ion precursor or surfactant, to achieve the target product morphology in high yield.^23,24^ It has also exclusively involved nanoparticles with kinetically controlled shape-formation mechanisms.^23,24^ Methods for colloidal synthesis of shape-controlled Au nanoparticles have been extensively developed but present a greater challenge than Pd for translation to electrodeposition, due to a multistep Au ion reduction pathway (Au^3+^ to Au^+^ to Au^0^) and more complex shape control mechanisms, often involving dilute Ag as a shape control agent.^28,29^ To enable the electrodeposition of more complex, but important, materials—such as shaped bimetallic nanoparticles and nanoparticles whose shape evolution is determined by selective surface passivation rather than solely by reaction kinetics—as well as shaped nanoparticles of materials other than Pd, it is necessary to understand the fundamental underlying principles that determine how ideal reagent concentrations for electrodeposition differ from those for colloidal growth.

In the present work, we combine morphological results of electrodeposition with *in situ* chronopotentiometry measurements to establish fundamental principles for tuning chemical solution and electrochemical parameters to broadly enable the direct translation of colloidal syntheses to electrodeposition, including for bimetallic materials and materials whose shape evolution is governed by selective surface passivation. Using the Ag-assisted synthesis of shaped (Ag)­Au nanoparticles as a model system, we show that controlling the local concentrations of reagents near growing nanoparticles on the electrode surface is critical, and that the bulk concentrations and mode of mass transport required to achieve optimal near-surface concentrations for shape control in electrodeposition differ from colloidal growth. In particular, for the case of both bimetallics and surface passivation-controlled growth, it is important that the different reagents (such as two different metal ions) are present near the surface of the working electrode at concentrations that result in them being reduced at the same relative rate as they would be in colloidal chemical growth. We further identify that establishing the correct reagent concentrations requires synergy between surfactant concentration, for tuning of bulk metal ion transport, and applied current, to control local diffusion and reaction rate. Together, these principles for parameter tuning in electrodeposition syntheses provide a general framework for the rational translation, design, and optimization of “colloidal inspired” electrodeposition syntheses for shaped metal nanostructures.

## Results and Discussion

### Experimental Considerations for (Ag)Au Nanoparticle Electrodeposition

Colloidally synthesized (Ag)Au nanoparticles have shapes that are surface passivation-controlled, with clear shape evolution to different morphologies—from octahedra to rhombic dodecahedra (RD) to truncated ditetragonal prisms (TDP) to concave cubes (CC)—based on the ratio of Ag^+^ to Au^+^ ions in the growth solution.^29–32^ Increasing [Ag^+^] in the bulk solution leads to an increased coverage of Ag on the growing Au nanoparticle surface, stabilizing increasingly higher-index facets as a result. In contrast to our prior electrodeposition synthesis work, where shape was solely controlled by metal ion reduction kinetics,^23,24^ a “colloidal inspired” electrodeposition synthesis for shaped (Ag)Au particles is more challenging to design in that it requires tuning of both reduction kinetics (matching the chronopotentiometry to the benchmark OCP for the colloidal synthesis) and surface effects (extent of Ag coverage/passivation). Therefore, (Ag)Au shaped nanoparticles were selected as a model system for translating a complex bimetallic colloidal synthesis to electrodeposition. The electrodeposition of (Ag)Au nanoparticles was conducted in an electrochemical cell with a three-electrode configuration, including a glassy carbon (GC) working electrode, upon which the metal nanoparticles were grown, a counter electrode (either a platinum (Pt) wire or a GC rod, immersed in solution or separated in a bridge tube containing 0.1 M H_2_SO_4_ supporting electrolyte; *vide infra*) and a reference electrode (Hg/Hg_2_SO_4_; sat. K_2_SO_4_ electrolyte; separated in a bridge tube containing 0.1 M H_2_SO_4_ supporting electrolyte) (Figure 1B).

**Figure 1:**
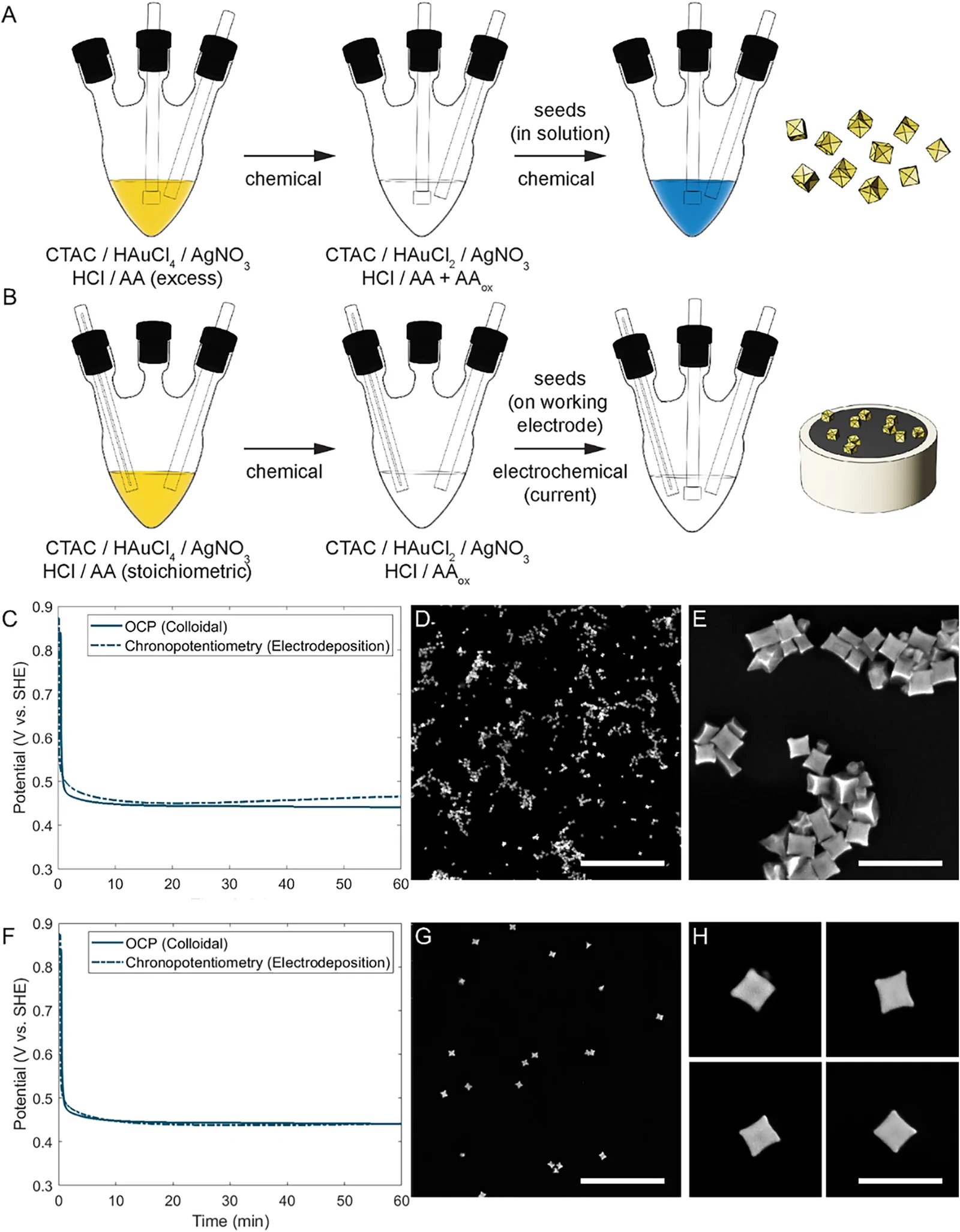
(A, B) Schemes comparing the steps of the (A) colloidal synthesis of (Ag)Au concave cubes (CC) and (B) electrodeposition of (Ag)Au CC, including the initial chemical preparation of the Au^+^ precursor. (C) Comparison between OCP measurement of colloidal CC growth (solid line) and chronopotentiometry measurement (dashed line) of galvanostatic electrodeposition of CC in 125 mM cetyltrimethylammonium chloride (CTAC) with 20.9 μM Ag^+^ and −0.815 μA/cm^2^ applied current for 60 min, onto Au seeds (200-fold diluted seed colloid). (D, E) Representative SEM images of electrodeposited CC nanoparticles, showing (D) deposition density and (E) particle morphology. (F) Comparison between OCP measurement of colloidal CC growth (solid line) and chronopotentiometry measurement (dashed line) of galvanostatic electrodeposition of well-separated CC in 125 mM CTAC with 30.0 μM Ag^+^ and −0.815 μA/cm^2^ applied current for 60 min, using a decreased seed concentration (500-fold diluted seed colloid). (G, H) Representative SEM images of electrodeposited CC nanoparticles, showing (G) deposition density and (H) high-magnification images of individual CC particles. Scale bars: 5 μm (D, G) and 500 nm (E, H). Triplicates of C–H are provided in the Supporting Information. Solid trace in (C, F) adapted with permission from ref 21. Available under a Creative Commons CC-BY 4.0 license. Copyright 2026 Halford, Sublett, and Personick.

The electrodeposition of shaped-controlled Au and (Ag)Au nanoparticles presents an additional experimental consideration, which is the importance of the oxidation state of the Au precursor prior to nanoparticle formation and shape evolution. In colloidal syntheses of shaped nanoparticles, Au follows a multistep reduction pathway in which a Au^3+^ species, often introduced as HAuCl_4_, is first reduced to Au^+^ in a two-electron reduction by a chemical reducing agent, commonly ascorbic acid (AA) (Figure 1A).^32,28^ In an acidic solution, AA is a weak reducing agent and the further reduction of Au^+^ to Au^0^ is kinetically slow, proceeding at a negligible rate.^32–34^ The subsequent introduction of preformed Au seed particles to the Au^+^-containing growth solution then catalyzes the one-electron reduction of Au^+^ ions to Au^0^ by AA (or another weak chemical reducing agent), leading to nanoparticle growth.^34–36^ Importantly, the reduction of Au^3+^ directly to Au^0^ by a strong reducing agent does not produce analogous nanoparticle products to the multistep Au^3+^/Au^+^/Au^0^ pathway. It is difficult to access the multistep reduction of Au ions in a purely electrochemical process because the reduction potential of AuCl_2_^–^ to Au^0^ is more positive (+1.15 V vs SHE) than that of AuCl_4_^–^ to AuCl_2_^–^ (+0.93 V vs SHE), and thus favorable under conditions where the reduction of the AuCl_4_^–^ precursor to form AuCl_2_^–^ would take place.^37^

To obtain an Au^+^ ion source for use in electrodeposition, we added a stoichiometric amount of AA and HAuCl_4_ to the growth solution prior to electrodeposition, causing reduction of Au^3+^ to Au^+^. Since the AA and Au salt are in an equimolar ratio and all Au^3+^ is rapidly converted to Au^+^, we assume that all AA is oxidized (AA_ox_) during this reaction. This assumption is supported by the disappearance of the 275 nm peak associated with AA in the UV–vis spectrum of the growth solution (Figure S1A). AA_ox_ is a reducing agent, but is weak and kinetically slow compared to reduced AA,^38^ so it is unlikely to contribute significantly to Au^+^ reduction on the short time scale of the electrodeposition experiments. However, it is critical to prevent the rereduction of AA_ox_ to AA, which could occur electrochemically at the working electrode or thermally at the counter electrode. Indeed, when a Pt wire counter electrode was inserted directly into the growth solution during electrodeposition of Au particles from a solution containing 1:1 [AA]: [Au^3+^], significant colloidal nanoparticle formation occurred in solution (Figure S1C–D). Au also plated on the Pt counter electrode, which was expected from literature reports.^39,40^ Additionally, when working electrodes both with and without preformed seeds dropcast on their surface were immersed overnight in a growth solution along with a Pt counter electrode, Au deposits were observed both in solution and on the working electrode, even without the application of current (Figure S1E,F). Isolation of the Pt counter electrode in a fritted bridge tube containing 0.1 M H_2_SO_4_ or replacement of the Pt counter electrode with a GC counter electrode—a material with far lower affinity for AA and AA_ox_—fully inhibited the generation of colloidal nanoparticles in the growth solution (Figure S1B). When these changes were made to the counter electrode, no Au particle growth was seen on the working electrode without the application of current/potential, further implying that the AA_ox_ to AA reduction leading to chemical nanoparticle growth was thermal in nature (taking place at the counter electrode),^41^ rather than electrochemical (taking place at the working electrode). To ensure that AA_ox_ was not (1) being rereduced or (2) capable of acting as a weak reducing agent during the time scale of electrodeposition experiments, UV–vis spectra of all growth solutions were taken immediately following electrodeposition to ensure that no Au^0^ nanoparticles formed in solution.

### Establishing Design Parameters for Shaped (Ag)Au Nanoparticle Electrodeposition

With these considerations in mind, we adapted the colloidal synthesis for (Ag)Au CC to electrodeposition. Since the shape direction between different Ag^+^ concentrations is not due to a differential change in the kinetics of metal ion reduction but rather selective passivation of specific facets by Ag, OCP measurements of syntheses for different shaped colloidal (Ag)Au nanoparticles are roughly identical (0.45 ± 0.025 V vs SHE) in mixed potential.^21^ However, it is still important to have optimal overall reaction kinetics in addition to the specific shape-directing role of surface passivation by Ag. As a first step for translation of the colloidal conditions to electrodeposition, chronoamperometry experiments were used to determine an initial range of currents most appropriate for applied-current electrodeposition of CC. Potentials of +0.44 and +0.46 V vs SHE, selected to match the OCP of colloidal CC synthesis, were applied for 60 min to a growth solution containing 100 mM cetyltrimethylammonium chloride (CTAC), 18.2 mM HCl, 0.45 mM HAuCl_4_, 0.45 mM AA, and 92.0 μM AgNO_3_, with 1 μL of undiluted Au seeds dropcast and dried on the working electrode. These conditions are analogous to the colloidal synthesis conditions for CC except AA is stoichiometric rather than in excess and the seeds are dropcast on the working electrode rather than introduced in suspension to the growth solution. Based on the steady-state currents measured when either +0.44 or +0.46 V vs SHE was applied (Figure S2), a range of possible applied currents from between −0.45 μA/cm^2^ and −1.35 μA/cm^2^ was selected.

Experiments using galvanostatic (constant current) electrodeposition coupled with chronopotentiometry were then carried out to establish the required (1) chemical composition of the growth solution and (2) electrochemical parameters for electrodeposition of CC. The empirical development of the electrodeposition parameters for CC led to the emergence of fundamental principles for synthesis design that then informed the development of electrodeposition methods for other (Ag)Au shapes. In particular, analogously to colloidal synthesis, the relative concentrations of Ag^+^ and Au^+^ near the surface of the growing metal nanoparticles were critical to successful surface passivation-controlled shaped development of CC. However, the dynamics of the near-surface concentrations of Ag^+^ and Au^+^ differ between colloidal and electrochemical growth, requiring the tuning of additional parameters to achieve the correct flux of Ag^+^ and Au^+^ ions in the electrochemical case. In colloidal growth, metal ions are depleted at the nanoparticle surface based on the rate of surface reaction (reduction by the chemical reducing agent) and replaced via passive mass transport from the bulk solution to establish a concentration gradient that eventually influences the steady-state growth rate.^21^ In electrodeposition, positively charged ions are attracted to the negatively polarized working electrode surface, leading to their reduction and rapid depletion at the electrode surface. In this case, passive mass transport is insufficient to compensate for the rapid depletion of metal ions and establish the appropriate concentration gradients of Ag^+^ and Au^+^ ions for the rate of metal ion reduction in the electrodeposition synthesis to mimic the colloidal reaction. As a result, constant moderate mechanical stirring (∼300 rpm) is required to refresh metal ion concentrations near the working electrode surface throughout the reaction—an adjustment which we have previously made empirically in the design of colloidal-inspired Pd electrodeposition syntheses but not rationalized mechanistically.^23,24^ As well as adding mechanical stirring to the electrodeposition synthesis, fine-tuning of metal ion (Au^+^ and Ag^+^) diffusion to the electrode was necessary to successfully electrodeposit CC.

Three central parameters were identified as the primary controlling factors for modulating diffusion to establish appropriate near-surface concentrations of metal ions during electrodeposition: [Ag^+^], [CTAC], and current density. The ideal growth solution for electrodeposition of CC contained 125 mM CTAC, 18.2 mM HCl, 0.45 mM HAuCl_4_, 0.45 mM AA, and 20.9 μM AgNO_3_. One μL of a 200-fold diluted Au seed colloid was dropcast and dried directly on the working electrode, and −0.815 μA/cm^2^ was applied for 60 min, leading to formation of well-defined CC (23 ± 7 particles per μm^2^) and a corresponding chronopotentiometry measurement that matched the OCP measured for colloidal CC growth, indicating optimal reduction kinetics (Figures 1B–E, S3, and S4A). This represents a 25% increase in the concentration of CTAC relative to the colloidal growth solution and a 77% decrease in the concentration of AgNO_3_, decreasing the ratio of Ag^+^:Au^+^ from 1:5 to ∼1:22. In colloidal growth, a 1:22 Ag^+^:Au^+^ ratio would yield a mixture of RD, TDP, and {210} or {310} bipyramids, representing a transition point between the two facets (Figure S5). The decreased bulk [Ag^+^] required for electrodeposition of CC implies that transport of Ag^+^ to the working electrode may be enhanced during electrodeposition relative to diffusion of Ag^+^ to seeds during colloidal growth due to the charge attraction between metal cations and the working electrode. A charge attraction would not occur in the same way for Au ions, which are present as an AuCl_2_^–^ complex. An attraction of Ag^+^ to the electrode would also lead to rapid near-surface depletion of metal ions overall, which then would need to be refreshed by convective mass transport (mechanical stirring) to achieve appropriate metal ion reduction kinetics to enable appropriate shape control by Ag^+^ (Figure S6). Once optimized for a 300 rpm stir rate, the morphology of electrodeposited particles was sensitive to the speed of mechanical stirring within ±100 rpm (Figure S7), with higher stir rates leading to shape changes associated with increased Ag coverage of the Au particles and lower stir rates leading to shape changes associated with decreased Ag coverage of the Au particles and/or decreased electrodeposition overall (*vide infra*). Synergistically with stirring, the [CTAC] is important for more finely tuning the diffusion of metal ions necessary to establish the appropriate overall reduction kinetics and help control the [Ag^+^] near the working electrode, likely by counterbalancing the acceleration of metal ion diffusion to the growing nanoparticles driven by the negatively charged electrode. Of note, the increased [CTAC] required to achieve desired shape direction in electrodeposition does not simply offset increased metal ion transport induced by stirring, as unstirred electrodeposition results are equivalent with the typical [CTAC] used for colloidal synthesis (100 mM) and increased [CTAC] used for shape-controlled electrodeposition (125 mM) (Figure S6). Finally, the applied current density, which needed to be finely tuned to produce CC, directly influences the metal ion reduction rate and likely also plays a role in the local diffusion dynamics of reagents, once again tuning the Ag^+^ concentration gradient in the near surface region and therefore indirectly modulating the rate of Ag deposition on the growing Au nanoparticles.

The CC electrodeposition was further optimized to improve the spacing of particles by adjusting the surface density of seed particles deposited on the WE, which also required a slight reoptimization of the [Ag^+^]. Decreased [Ag^+^] was required to produce CC at increased seed coverages while increased [Ag^+^] was required at decreased seed coverages (Figures S8–S10). This result differs from what might be expected based on the bulk [Ag^+^] per seed, but we hypothesize that the bulk [Ag^+^] and the [Ag^+^] in the near surface region differ and that this difference is dependent on seed coverage. An increased coverage of seeds leads to increased diffusional coupling between closely spaced seed particles (*vide infra*), which could drive increased transport of Ag^+^ to the near surface region, increasing the effective [Ag^+^] at the electrode even as it is lower in the bulk solution.^42–44^ At more diffuse seed coverages, diffusional coupling would lessen, decreasing the difference between the near-surface and bulk [Ag^+^]. Correctly balancing the bulk [Ag^+^] with the diffusional coupling effects from seed coverage can, therefore, allow for a consistent ratio of Ag^+^:Au^+^ near the growing nanoparticles, even though the ratio of seeds to bulk [Ag^+^] is not constant across all conditions. By tuning the surface density of dropcast seed particles and adjusting [Ag^+^] accordingly, the particle surface density was tunable from 39 ± 25 to 3 ± 1 particles per μm^2^ while still maintaining the CC shape.

Well-formed and well-dispersed CC (3 ± 1 particles per μm^2^) were deposited using a 2.5-fold lower concentration of seed colloid than the initial experiments (500-fold seed colloid dilution) and 30.0 μM Ag^+^ (a ∼43% increase) (Figures 1F–H, S4B, and S10–S11). The need for these changes in the [Ag^+^]:[Au^+^] ratio in solution implies that the facilitated diffusion of Ag^+^ to growing Au nanoparticles on the working electrode (and, therefore, the ability to form the correct CC shape) is dependent on seed coverage, which influences diffusion at the electrode surface. This differs from colloidal growth, where changing the concentration of seeds in solution generally changes particle size while maintaining the same particle shape.^31^ The growth of Au nanoparticles onto the dropcast seeds with AuCl_2_^–^ serving as the Au precursor was confirmed via control experiments in a standard growth solution (1) without dropcast seeds on the electrode and (2) with dropcast seeds but without HAuCl_4_ or AA added to the deposition solution. In both cases, no particle growth was observed and the chronopotentiometry was shifted to a more positive potential than under typical growth conditions (Figures S12–S13). The lack of particle growth when HAuCl_4_ or AA were not present in the growth solution implies that the dropcast Au seeds are sufficiently stable that they do not dissolve and contribute Au ions to particle growth (Figure S13).

Anodic stripping voltammetry (ASV) was used to confirm the presence of metallic Ag, characterize the type of Ag coverage, and calculate the ratio of Ag:Au in the electrodeposited nanoparticles at this well-dispersed condition (Figures S14–S18 and Tables S1–S3). Following (Ag)Au CC electrodeposition, Ag was stripped in 500 mM H_2_SO_4_, and, next, Au was stripped in a mixture of 100 mM KClO_4_ plus 10 mM KBr, consistent with other literature procedures for ASV of AgAu bimetallic nanoparticles.^45^ Appropriate cleaning of samples to remove residual CTAC was necessary to obtain optimal and reproducible ASV data (Figures S14–S16; see Supporting Information for additional discussion). In triplicate ASV experiments, the stripping peak in H_2_SO_4_, which was not noted for a control using a bare GC electrode, confirmed the presence of metallic Ag in the electrodeposited (Ag)Au CC, while the peak potential (*E*_p_) for the Ag stripping peak (∼1.0 V vs SHE) was consistent with the literature value for a submonolayer coverage of underpotentially deposited metallic Ag on Au (Figure S17).^46^ Analogous ASV experiments were conducted for stripping of washed, diluted colloidal (Ag)Au particles that were dropcast and dried on a glassy carbon electrode (Figure S18), and integration of the Ag and Au stripping peaks for both electrodeposited and colloidal samples was used to obtain ratios of their charge (Figures S17–S18). From ASV, a ratio of 0.011 ± 0.001 Ag:Au was calculated for electrodeposited (Ag)Au CC, while a ratio of 0.012 ± 0.002 Ag:Au was calculated for colloidal (Ag)Au CC (Table S3); both ratios are consistent with the ∼0.012 Ag:Au ratio obtained by ICP-OES spectroscopy for colloidal (Ag)Au particles of a similar size.^29^ These results suggest that in the electrodeposited (Ag)Au CC, Ag is metallic—implying reduction by a UPD mechanism—and present at the particle surface in a submonolayer coverage, similarly to the colloidal (Ag)Au CC.^29^ Of note, considerable debate has occurred in the literature about the mechanism for Ag-assisted Au nanorod growth and the relationship, if any, between this mechanism and Ag UPD shape control of Au particle growth.^28,47–49^

In contrast to the well-formed CC accessible at lower seed coverages, a greater shape dispersity of particles was observed with an increased coverage of seeds on the working electrode (39 ± 25 particles per μm^2^; Figure S10), even at the optimized [Ag^+^]. We attribute this to a disturbance of the flux of Au^+^ and Ag^+^ ions to the seed sites because the seeds are located too close together, causing the diffusion of reagents to each seed site to become coupled.^50^ This phenomenon, known as interparticle diffusion coupling,^42,50,51^ is likely to be perturbative to CC growth, due to the need to tightly control the ratio of [Ag^+^]:[Au^+^] at each seed site to obtain the correct shape-directing passivation effect of Ag. At the standard seed coverage (23 ± 7 particles per μm^2^), as well as 1.5-fold and 2.5-fold decreases in seed colloid concentration (corresponding to 13 ± 9 and 3 ± 1 particles per μm^2^, respectively), the chronopotentiometry for the electrodeposition closely matches the OCP of colloidal growth in both potential and line shape (Figures 1C,F and S8A). In contrast, the chronopotentiometry corresponding to a 20-fold increase in seed colloid concentration (39 ± 25 particles per μm^2^), which produces a comparatively poor shape yield of CC, is shifted slightly negative from the OCP benchmark (Figure S8 and Table S4). Additionally, the chronopotentiometry trace for the higher seed concentration shows a much sharper and more “squared-off” initial dip in potential compared to the OCP for colloidal CC growth, likely related to a difference in the initial diffusion of reagents to and/or reduction of metal ions at high coverages of Au seed particles adsorbed on the electrode vs in colloidal suspension. This further supports the possibility of coupling of diffusion spheres between closely spaced seed particles adsorbed on the electrode in the case of electrodeposition with highly concentrated seeds.

### Applying the Design Parameters to the Surface-Passivation Controlled Electrodeposition of Other (Ag)Au Shapes

Using the [Ag^+^] required for the successful electrodeposition of CC as a foundation, we carried out electrodeposition with a gradient of [Ag^+^] predicted to correspond to each of the other shapes, while keeping all other chemical and electrochemical parameters identical to those established for CC, including the original seed concentration (Figures 2 and S19). Octahedral, RD, and TDP nanoparticles are expected to form at 1%, 10%, and 40–60% of the [Ag^+^] required for colloidal CC formation, respectively, if [Au^3+^] is held constant.^29,32^ In the absence of Ag^+^, a mixture of {111}-faceted and truncated structures should be produced.^32^

**Figure 2:**
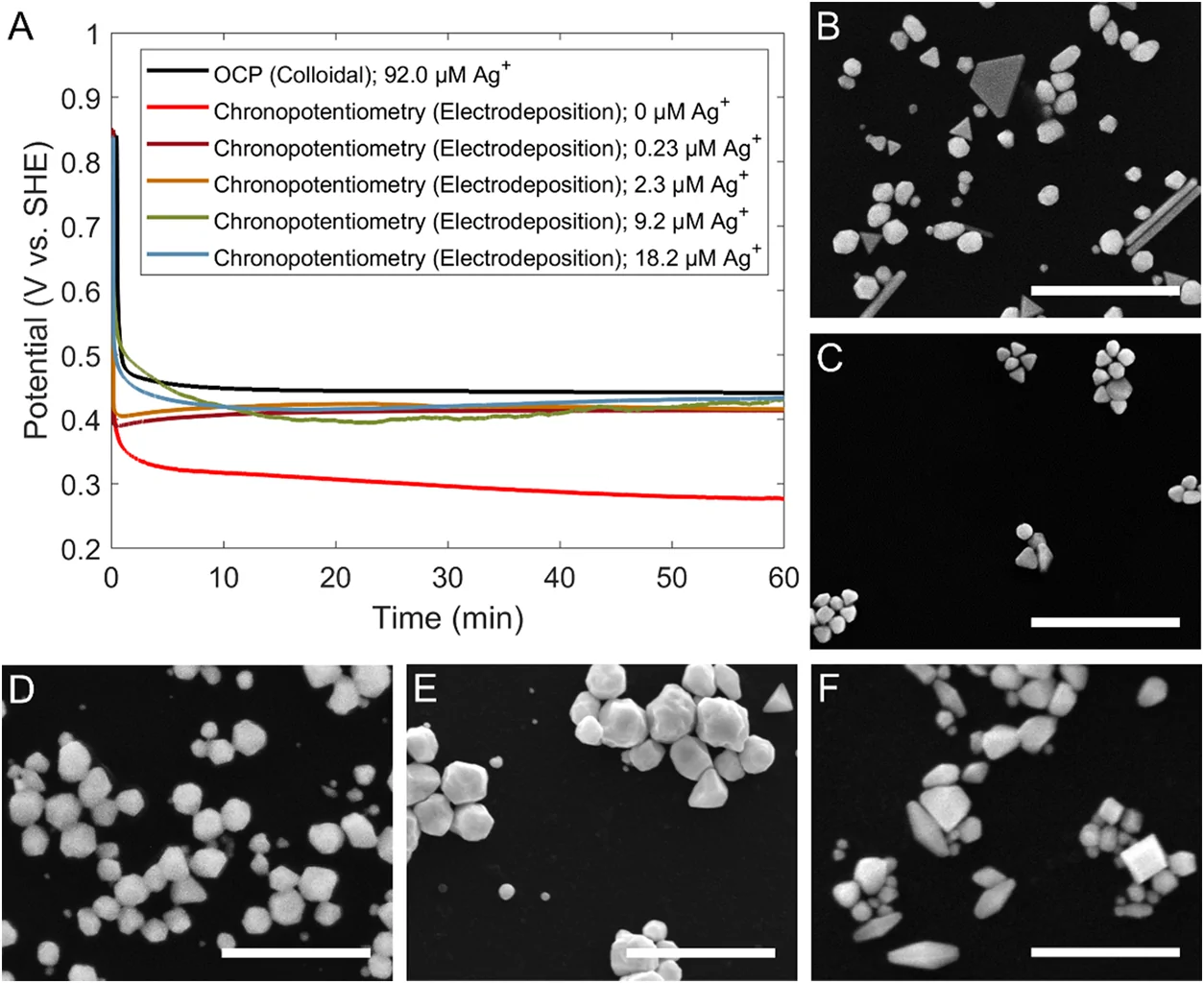
(A) Comparison between OCP measurements of colloidal (Ag)­Au CC growth and chronopotentiometry measurements of galvanostatic electrodeposition of (Ag)Au nanoparticles in 125 mM CTAC with varied [Ag^+^] and −0.815 μA/cm^2^ applied current for 60 min, onto Au seeds (200-fold diluted seed colloid). (B–F) SEM images of electrodeposited (Ag)Au nanoparticles with (B) 0 μM Ag^+^ (monometallic Au; red trace), (C) 0.23 μM Ag^+^ (dark red trace), (D) 2.3 μM Ag^+^ (orange trace), (E) 9.2 μM Ag^+^ (green trace), and (F) 18.2 μM Ag^+^ (blue trace). Scale bars: 500 nm. Black trace in (A) adapted with permission from ref 21. Available under a Creative Commons CC-BY 4.0 license. Copyright 2026 Halford, Sublett, and Personick.

When monometallic Au particles were electrodeposited (no AgNO_3_ added to the growth solution), their morphologies included a mixture of octahedra and truncated octahedra, triangular plates, nanorods, and smaller pentatwinned particles, as expected from colloidal precedent (Figures 2B and S20). The chronopotentiometry trace for monometallic Au electrodeposition was shifted ∼130 ± 25 mV more negative in potential than for the electrodeposition of CC, analogously to OCP measurements of colloidal growth solutions in the absence of Ag^+^ (Figures 2A and S20).^21^ This more negative potential corresponds to a higher rate of Au^+^ reduction in the absence of surface passivation by Ag.^21^ The similarity in both time-resolved potential and particle morphologies for monometallic Au vs (Ag)Au particle growth in electrodeposition and colloidal synthesis provides additional evidence that the role of Ag is the same in both synthesis environments: selective surface passivation and a corresponding overall slowing of the rate of Au^+^ reduction.

Electrodeposition with 0.23 μM Ag^+^—approximately 1% of the [Ag^+^] used to electrodeposit CC at the same seeding density—produced octahedral nanoparticles, as predicted (Figures 2C and S21). Intermediate concentrations of [Ag^+^], expected to produce RD and TDP, were less successful for shape-direction. Electrodeposition with 2.3 μM Ag^+^—approximately 10% of the concentration used to electrodeposit CC—did not produce RD, instead yielding rounded nanoparticles with some {110} or {h10}-like features, and a subpopulation of {110} bipyramids in some samples (Figure 2D). These products are what would be expected if the [Ag^+^] was slightly too high in colloidal growth. Adjusting the [Ag^+^] was insufficient to produce RD or increase the percentage of {110} bipyramids. Similarly, electrodeposition with 9.2 μM Ag^+^—approximately 44% of the concentration used to electrodeposit CC—did not generate TDP nanoparticles, instead producing a similar mixture of rounded nanoparticles, with some {110} bipyramids and shard-like {h10} bipyramids (Figure 2E). Doubling the concentration of [Ag^+^] from the expected value for TDP, to 18.2 μM, did lead to the production of {h10} bipyramids as the major product, with very few TDP also present (Figure 2F). In each of these cases, the chronopotentiometry measurements during electrodeposition were more negative in potential that the OCP measurements of the corresponding colloidal syntheses, indicating that, at minimum, these initial electrodeposition conditions do not have ideal reaction kinetics for the successful formation of these shapes (Figure 2A). The octahedra are less sensitive to reaction conditions even in colloidal syntheses, so they form successfully despite the reaction kinetics not being not identical to the colloidal reaction measured by OCP.

Since tuning the bulk [Ag^+^] was insufficient to yield high quality RD or TDP, we then used [CTAC] and current density to rationally design conditions for successful shape formation in these two cases, while keeping [Ag^+^] at the predicted concentrations of 2.3 μM and 9.2 μM, respectively. For RD, the initial electrodeposition conditions yielded shapes that suggested the [Ag^+^] at the surface of the electrode was too high (Figure 2D), so we increased [CTAC] while decreasing the current, based on the principles developed for CC. A minor increase in the [CTAC] to 140 mM (from 125 mM) combined with a decrease in current magnitude to −0.713 μA/cm^2^ (from −0.815 μA/cm^2^) were sufficient to produce well-faceted RD in high yield (Figures 3A,B and S22–S23). Of note, independently increasing CTAC concentration or decreasing the current density (−0.713 μA/cm^2^ to −0.662 μA/cm^2^) was insufficient, instead producing rounded nanoparticles (Figure S24), which implies that these two factors work synergistically. It was less apparent from the shapes produced for TDP under the initial electrodeposition conditions which direction the local [Ag^+^] should be adjusted (Figure 2E), particularly because TDP form over a larger range of [Ag^+^] colloidally. However, since both CC and RD electrodeposition required adjustments to decrease the local [Ag^+^], we hypothesized that syntheses along same mechanistic pathway would behave similarly. Indeed, an increase in [CTAC] to 130 mM coupled with a decrease in applied current to −0.739 μA/cm^2^ also led to a high yield of TDP (Figures 3A,C and S22–S23). The chronopotentiometry for the growth of both RD and TDP was shifted to higher potentials compared to the initial electrodeposition conditions, showing good agreement with OCP measurements of colloidal growth and confirming that ideal reaction kinetics had been established, along with successful surface passivation (Figure 3A).

**Figure 3:**
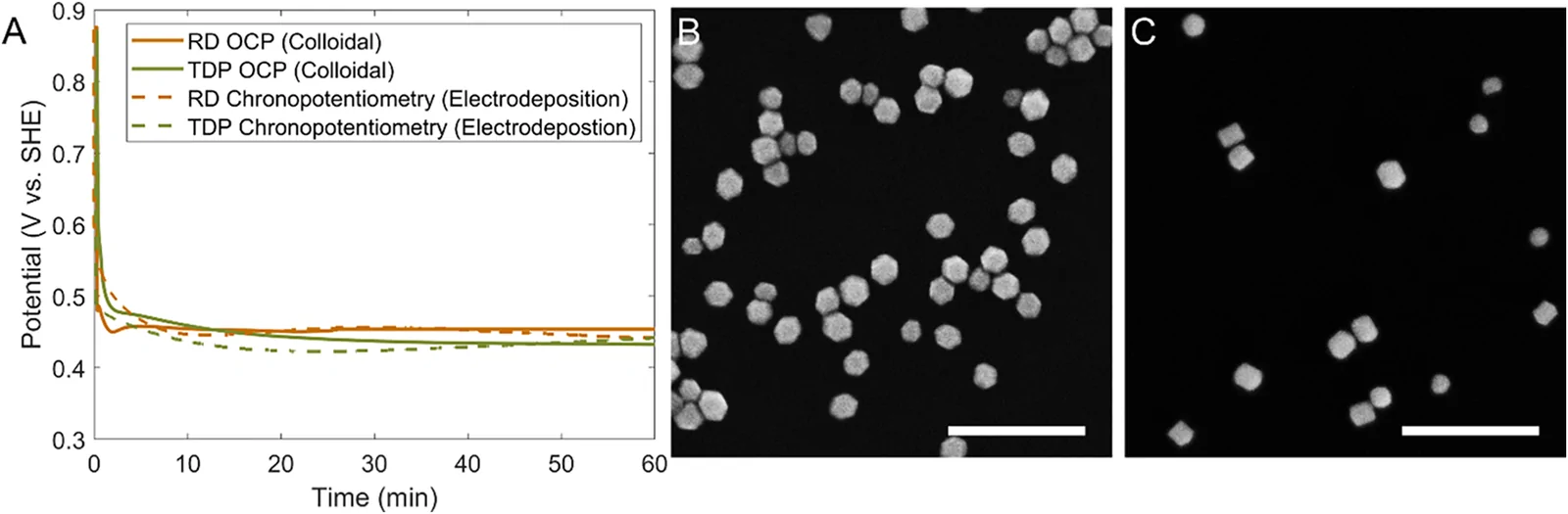
(A) Comparison between OCP measurements of colloidal RD (solid orange) and TDP (solid green) growth and chronopotentiometry measurements of galvanostatic electrodeposition of RD (dashed orange) and TDP (dashed green). RD were electrodeposited in 140 mM CTAC with 2.3 μM Ag^+^ and −0.713 μA/cm^2^ applied current for 60 min, onto Au seeds (200-fold diluted seed colloid). TDP were electrodeposited in 130 mM CTAC with 9.2 μM Ag^+^ and −0.739 μA/cm^2^ applied current for 60 min, onto Au seeds (200-fold diluted seed colloid). (B) Representative SEM image of electrodeposited RD. (C) Representative SEM image of electrodeposited TDP. Scale bars: 500 nm. Solid orange and green traces in (A) adapted with permission from ref 21. Available under a Creative Commons CC-BY 4.0 license. Copyright 2026 Halford, Sublett, and Personick.

After identification of conditions required for successful shape development for all of the morphologies, the electrodeposition was then carried out at a 2.5-fold decreased concentration of seed colloid (lower seed surface coverage) to again improve particle dispersion on the electrode (Figures 4 and S25). For each shape, [Ag^+^] was adjusted according to the scaling factor (1.43) established during the CC experiments for translation to lower seed coverage. Well-dispersed, well-defined shaped particles were successfully formed in each case (Figures 4 and S26–S28). Of note, while octahedra had formed successfully at the same current used for CC, based on the optimizations for RD and TDP, the current for the deposition of octahedra was decreased to −0.739 μA/cm^2^, successfully improving the correspondence between the chronopotentiometry and the OCP for this shape while maintaining particle quality (Figure 4A–C). The surface coverage density of these particles was comparable to (Ag)­Au CC electrodeposited at the same seeding conditions (3 ± 1 octahedral and truncated bipyramidal particles per μm^2^, 5 ± 2 RD per μm^2^, and 4 ± 2 TDP per μm^2^; Figure S29).

**Figure 4:**
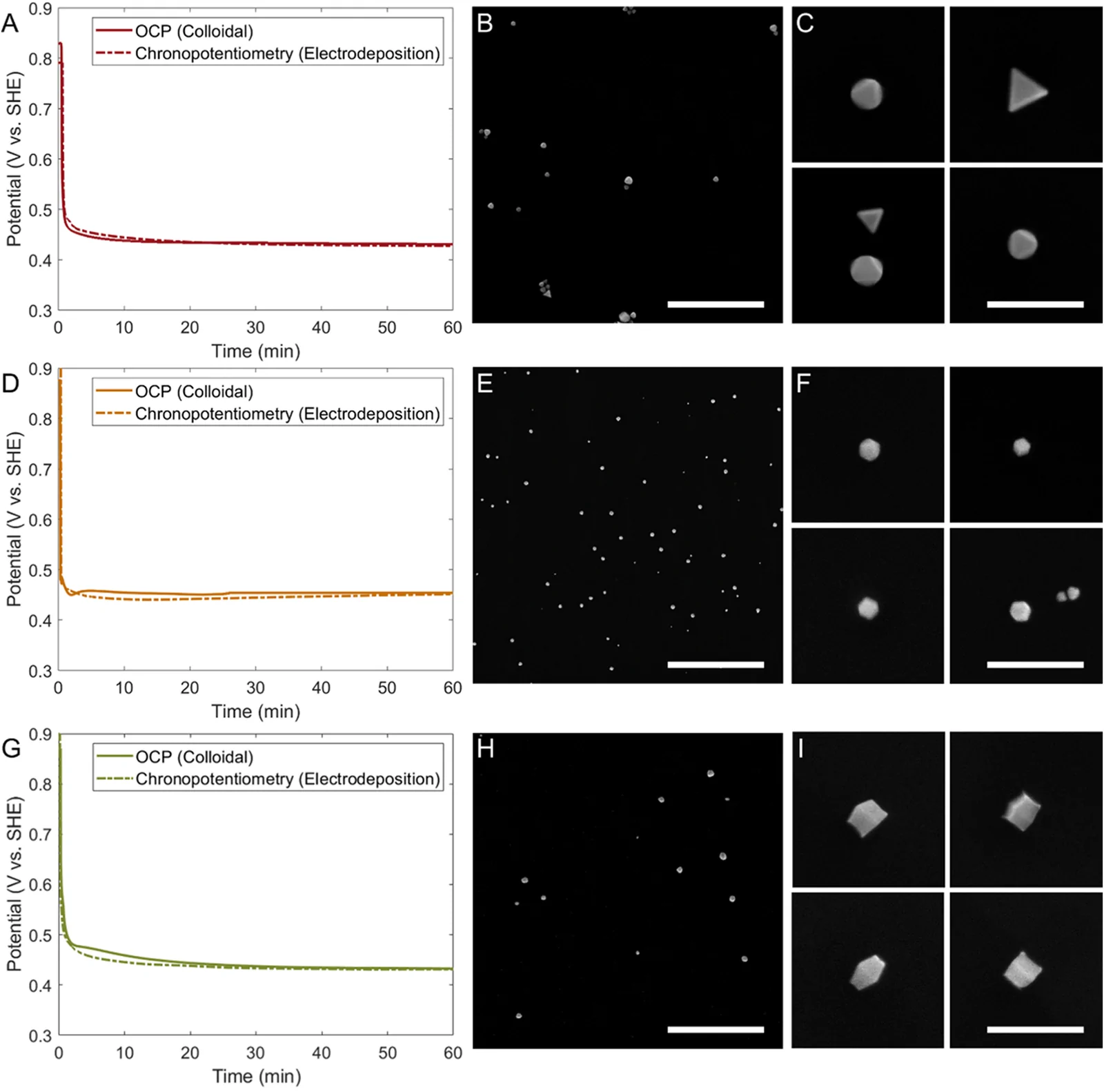
(A) Comparison between OCP measurement of colloidal octahedra growth and chronopotentiometry measurement of galvanostatic electrodeposition of octahedra. Octahedra were electrodeposited in 125 mM CTAC with 0.33 μM Ag^+^ and −0.739 μA/cm^2^ applied current for 60 min, onto Au seeds (500-fold diluted seed colloid). (B–C) Representative SEM images of electrodeposited octahedral and truncated bitetrahedral nanoparticles, showing (B) deposition density and (C) individual particle morphology. (D) Comparison between OCP measurements of colloidal RD growth and chronopotentiometry measurements of galvanostatic electrodeposition of RD. RD were electrodeposited in 140 mM CTAC with 3.3 μM Ag^+^ and −0.713 μA/cm^2^ applied current for 60 min, onto Au seeds (500-fold diluted seed colloid). (E, F) Representative SEM images of electrodeposited RD nanoparticles, showing (E) deposition density and (F) individual particle morphology. (G) Comparison between OCP measurements of colloidal TDP growth and chronopotentiometry measurements of galvanostatic electrodeposition of TDP. TDP were electrodeposited in 130 mM CTAC with 15.5 μM Ag^+^ and −0.739 μA/cm^2^ applied current for 60 min, onto Au seeds (500-fold diluted seed colloid). (H–I) Representative SEM images of electrodeposited RD nanoparticles, showing (H) deposition density and (I) individual particle morphology. Scale bars: 5 μm (B, E, H) and 500 nm (C, F, I). Triplicates are provided in the Supporting Information. Solid OCP traces in (A), (D), and (G) adapted with permission from ref 21. Available under a Creative Commons CC-BY 4.0 license. Copyright 2026 Halford, Sublett, and Personick.

### Testing the Generalizability of the Design Parameters: (Ag)­Au Nanoparticle Electrodeposition in Bromide-Containing Surfactant

(Ag)Au nanoparticles grown colloidally in cetyltrimethylammonium bromide (CTAB) surfactant—rather than CTAC—with all other growth conditions identical to standard CC-forming conditions, form a convex tetrahexahedral (THH) morphology rather than CC.^30,32^ The mechanism for the change from concave to convex particle morphology resulting from the switch from chloride to bromide is posited to be a lower coverage of Ag on the surface combined with the increased thermodynamic favorability of the convex THH morphology compared to a concave morphology.^32^ With this in mind, we sought to electrodeposit (Ag)Au particles in CTAB as a test case for the principles for modulating reagent diffusion and growth kinetics that we established for (Ag)Au electrodeposition in CTAC.

The OCP measurement of colloidal (Ag)Au THH growth in CTAB was shifted slightly more negative in potential (25–45 mV, depending on reaction progress) compared to the OCP for (Ag)Au CC (Figure S30). In addition to the change from a chloride counterion to a bromide counterion, THH growth is also conducted at 40 °C due to the poor solubility of CTAB at room temperature, and this temperature change must be accounted for when tuning the reduction kinetics in electrodeposition. The OCP of the THH was compared to both OCP and chronopotentiometry measurements of CC synthesized at 40 °C to isolate the influence of temperature from that of bromide (Figure S30). The increased temperature shifted the OCP measurement for the colloidal CC more negative as a result of the enhanced reducing strength of AA at higher temperatures, while the temperature change increased the measured potential during CC electrodeposition, reflecting facilitated electron transfer during electrodeposition at higher temperatures. Thus, we hypothesized that an increased magnitude of current density would be required to achieve appropriate reaction kinetics during electrodeposition of THH at 40 °C, represented by good agreement between the chronopotentiometry and OCP.

The morphology of (Ag)Au nanoparticles electrodeposited in 125 mM CTAB under otherwise the same conditions used for the well-dispersed CC was indeed convex, but rather than well-faceted THH nanoparticles, the products were spiky and stellated, indicating poor shape control (Figure S31). Similar spikey (Ag)Au products are known to occur colloidally when the [Ag^+^] and/or the effective Ag surface coverage is too high for CC/THH formation.^32^ These results demonstrate an increase in the facilitation of Ag^+^ diffusion during (Ag)Au electrodeposition in CTAB, possibly due to the increased binding affinity of bromide with both Ag^+^ and the nanoparticle surface. Increasing the current density—as predicted from the temperature experiments—to −0.866 μA/cm^2^ so that the chronopotentiometry matched the OCP (correct reaction kinetics), combined with decreasing the concentration of [Ag^+^] to 1.3 μM (a 96% decrease compared to the CC) to compensate for this enhanced diffusion, led to the formation of THH and convex side products (Figure S32). While these particles were clearly THH, they were not as high quality as the (Ag)Au shaped particles synthesized in CTAC. However, we also observed that the THH had a higher coverage density on the electrode than the particles electrodeposited in CTAC, possibly due to the preformed seeds that were synthesized in CTAB being of a higher concentration than those synthesized in CTAC.

Therefore, to improve particle quality, we further decreased the seed coverage of the Au seeds synthesized in CTAB to better separate growth sites and minimize interparticle diffusional coupling. With a 2-fold decrease in seed concentration, minor reoptimization of [CTAB] (decreased to 115 mM) and current density (decreased to −0.713 μA/cm^2^) was required to achieve the correct reaction kinetics, along with an increase of the concentration of [Ag^+^] to 2.3 μM (92% less than the concentration used to synthesize CC) as was previously the case when moving to lower seed concentrations. These adjustments yielded well-formed and well-separated THH particles (11 ± 4 particles per μm^2^; Figures 5 and S33–S36), with some corner truncations (Figure S37). The ability to electrodeposit well-formed THH nanoparticles with appropriate, rational adjustments to reaction conditions further confirms the generalizability of the design parameters for translation of colloidal synthesis to electrodeposition.

**Figure 5:**
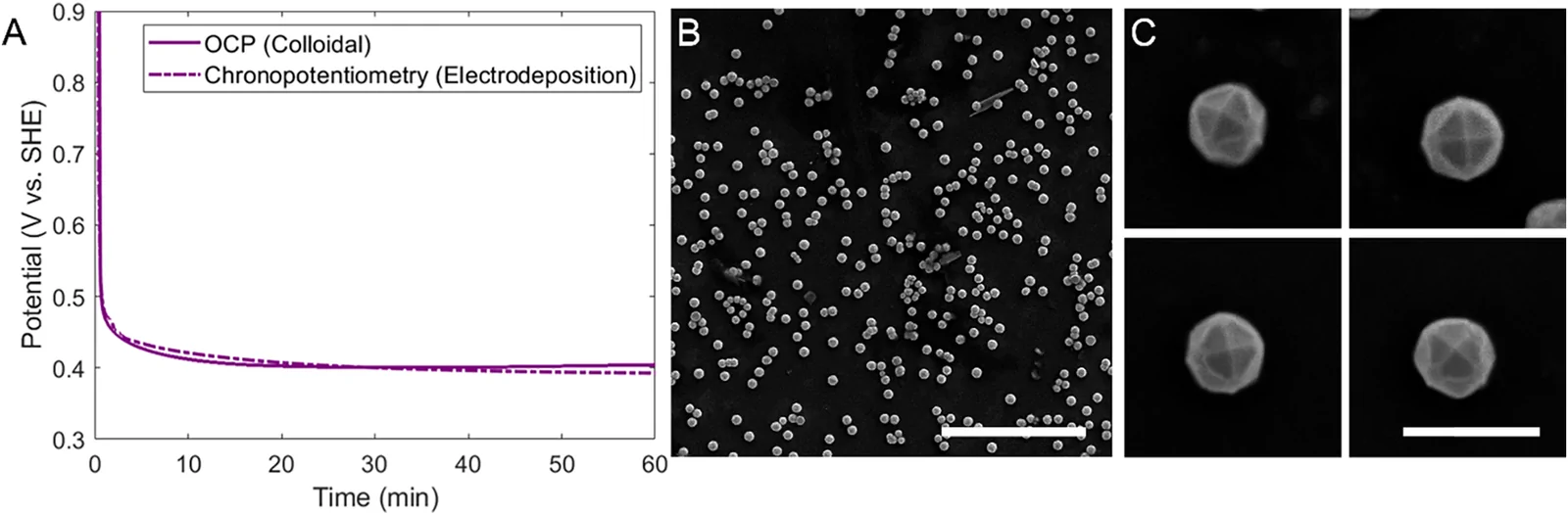
(A) Comparison between OCP measurement of colloidal THH growth and chronopotentiometry measurement of galvanostatic electrodeposition of THH in 115 mM CTAB with 2.3 μM Ag^+^ and −0.713 μA/cm^2^ applied current for 60 min, onto CTAB-capped Au seeds (1000-fold diluted seed colloid). (B–C) Representative SEM images of electrodeposited THH, showing (B) deposition density and (C) particle morphology. Scale bars: 5 μm (B) and 500 nm (C). Triplicates are provided in the Supporting Information.

## Conclusion

The direct colloidal-to-electrodeposition translation approach presented in this work provides a method for electrochemically synthesizing a range of well-defined faceted (Ag)Au nanoparticles and, more broadly, demonstrates the feasibility of shape control via facet-selective surface passivation during the electrodeposition of bimetallic nanoparticles, where previous colloidal-inspired electrodeposition work had been confined to kinetic shape control of monometallic nanostructures. Importantly, this work establishes and validates the key design parameters for rationally adapting colloidal syntheses to current-driven growth on an electrode surface: adding convective mass transport (to compensate for rapid depletion of metal ions near the electrode surface), increasing surfactant concentration (to control bulk diffusion in the presence of the charged electrode) and adjusting current density (to control local diffusion and reaction rate) in tandem to achieve optimal growth kinetics. For surface-passivation-controlled syntheses, these adjustments should be combined with a decrease in the concentration of ionic shape-directing species to compensate for facilitated diffusion to the charged electrode. By using these handles to set growth kinetics and bulk reagent diffusion appropriately, while finely controlling local concentrations of metal ions near the working electrode through both bulk and near-surface diffusion, it is also possible to tune the relative rates of reduction of two different metals. This method presents opportunities for the controlled electrodeposition of other kinds of multimetallic nanostructures and complex materials, particularly in combination with recent findings regarding the importance of the instantaneous concentration of different metal ions, rather than the reduction kinetics of their precursors, in controlling the differential incorporation of various metals in multimetallic colloidal particles.^52^ Our fundamental insights regarding reagent diffusion at nanoparticles immobilized on surfaces are likely to also be relevant for other synthetic translations from solution to surface growth, such as the *chemical* growth of nanoparticles on surfaces or support materials, although local diffusion in that case would need to be tuned chemically: possibly by modulating surfactant density at the surface, the solution temperature, or by temporally controlling the introduction of different precursors.

## Methods

### Materials

Cetyltrimethylammonium chloride solution (CTAC, Lot No. STBL3571, 25 wt % solution in water), cetyltrimethylammonium bromide (CTAB, BioXtra Lot No. SLCJ8356 ≥ 99%), l-ascorbic acid (AA, ACS reagent grade ≥ 99%), sodium borohydride (NaBH_4_, 99.99% trace metal basis), gold­(III) chloride trihydrate (chloroauric acid, HAuCl_4_, ≥ 99.9% trace metals basis), silver nitrate (AgNO_3_, 99.9999% trace metal basis), and potassium perchlorate (KClO_4_, ACS reagent grade ≥ 99%) were purchased from MilliporeSigma. Hydrochloric acid (HCl, 1 M solution) was purchased from VWR. Concentrated hydrochloric acid (HCl, ACS Plus grade), concentrated nitric acid (HNO_3_, ACS Plus grade), sulfuric acid solution (H_2_SO_4_, 0.5 M solution), methanol (MeOH, ACS reagent grade), and potassium bromide (KBr, 99.999%) were purchased from Thermo Fisher Scientific. Alumina powder (0.05 and 0.3 μm MicroPolish) was purchased from Buehler. Saturated potassium sulfate electrode storage solution (K_2_SO_4_, in water) was purchased from Koslow Scientific Co.

All chemicals were used without further purification and all solutions were prepared with ultrapure deionized (DI) water (18.2 MΩ resistivity, Milli-Q IQ 7000). All ascorbic acid, silver nitrate, and sodium borohydride solutions, as well as diluted seed solutions, were prepared immediately before use and remade fresh on the half-hour as needed. Potassium bromide stock for the potassium bromide and potassium perchlorate mixture used in anodic stripping of Au was remade daily.

### Synthesis of Au Seeds in CTAC

∼7 nm Au seeds were synthesized in CTAC according to a previously reported method.^31^ 10 mL of 100 mM CTAC and 250 μL of 10 mM HAuCl_4_ were added to a 20 mL scintillation vial and stirred to vortex. Once the solution was thoroughly mixed, 600 μL of freshly prepared 10 mM NaBH_4_ was rapidly injected under continued vortex stirring, inducing an instantaneous color change of the solution from yellow to red. The seed solution was allowed to stir for an additional minute to ensure uniformity in the dispersion of the reducing agent. The stirring was then halted and the seed solution was aged on the benchtop for 2 h to ensure degradation of excess NaBH_4_. Seeds were used within 24 h of synthesis. These “Au-CTAC seeds” were used for all nanoparticle growth conducted in CTAC.

### Synthesis of Au Seeds in CTAB

∼7 nm Au seeds were synthesized in CTAB according to a previously reported method.^30^ The “Au-CTAB seed” synthesis was identical to the synthesis of Au seeds in CTAC, with the exception of the use of 9.75 mL of 100 mM CTAB rather than CTAC. Following synthesis and aging, these seeds were stored in a 40 °C water bath and used within 24 h of synthesis.

### Colloidal Synthesis of (Ag)Au Nanoparticles

Shape-controlled colloidal (Ag)Au nanoparticles were synthesized in CTAC using a previously reported method.^29,31^ A growth solution was prepared at room temperature by adding 10 mL of 100 mM room-temperature CTAC (Sigma 25 wt % solution), 500 μL of 10 mM HAuCl_4_, 200 μL of 1 M HCl, and *x* μL of 10 mM freshly prepared AgNO_3_ (*x* = 0 μL for {111} products with mixed twin structures, 1 μL for octahedra, 10 μL for RD, 40 μL for TDP, and 100 μL for CC) to a 20 mL scintillation vial and swirling. 100 μL of 100 mM fresh ascorbic acid was added and the vial was swirled again. Au-CTAC seeds were serially diluted 1000-fold in 100 mM Sigma CTAC, and 100 μL of the diluted seeds were added to the growth solution. The reaction was swirled immediately after the addition of the seeds and then left undisturbed on the benchtop for ∼120 min.

(Ag)­Au THH were synthesized using a previously reported method.^30^ The synthesis was identical to the synthesis of (Ag)Au CC, with the following exceptions: the synthesis was conducted at 40 °C rather than room temperature, in 10 mL of 100 mM CTAB rather than CTAC, and Au-CTAB seeds were used rather than Au-CTAC seeds.

### General Procedure for Working Electrode Preparation

Glassy carbon electrodes (GCEs; 5 mm OD × 4 mm thick disk insert, Pine Research) were prepared by polishing successively with slurries of 0.3 μM and 0.05 μM alumina (MicroPolish Alumina, Buehler), rinsing with DI water and sonicating three times in a fresh vial of DI water between each polishing step. Polished GCEs were stored in fresh DI water until use. Before use in OCP measurements or electrodeposition experiments, GCEs were inserted into a Teflon electrode tip holder (Pine Research), rinsed with water and dried with an air gun.

### Open-Circuit Potential Measurement of Colloidal Particle Syntheses

Open-circuit potential measurements of colloidal (Ag)Au nanoparticle syntheses were taken according to our previously reported method.^21,24^ An electrochemical cell was prepared in a 4- or 5-neck heart-shaped flask (Gamry Instruments Dr. Bob’s Cell) using a glassy carbon electrode (GCE; 5 mm OD × 4 mm thick disk insert, Pine Research) as the working electrode and a Hg/Hg_2_SO_4_ reference electrode (Koslow Scientific) as well as a miniature stir bar (3 mm × 10 mm; PTFE-coated). A counter electrode was not added. The reference electrode was isolated from the reaction solution by a bridge tube with a glass frit containing 100 mM sulfuric acid. The electrodes were immersed in a particle growth solution containing all reagents except for the chemical reducing agent and seed particles. The necks of the flask that were not in use were capped. The electrochemical cell was clamped above a stir plate, with a 40 °C water bath if applicable, and electrode leads were connected to the potentiostat (Gamry Instruments 1010E). The growth solution was stirred at 200 rpm for 10 min. After that time, an OCP measurement was started using the Gamry Framework software interface. The electrochemical cell was uncapped, and reducing agent and seeds were rapidly injected into the growth solution. Stirring was continued for 10 s after injection of seeds and reducing agent, then turned off. OCP data was analyzed using Gamry Echem Analyst software and MATLAB. A factor of +0.65 V was used to convert potentials from Hg/Hg_2_SO_4_ to the SHE scale. Any data points before 60 s prior to the introduction of both seeds and reducing agent were omitted from plotting of OCP traces of colloidal reactions to align the reaction start times.

### General Procedure for Galvanostatic Electrodeposition of (Ag)­Au Nanoparticles

All electrodeposition syntheses were conducted in a five-port glass electrochemical cell (Gamry Instruments Dr. Bob’s Cell) with a seeded GCE as the working electrode, a GC rod as the counter electrode (Gamry Instruments), a saturated Hg/Hg_2_SO_4_ reference electrode (Koslow Scientific), and a miniature stir bar (3 mm × 10 mm; PTFE-coated). One μL of a solution of Au seeds, diluted between 10-fold and 1000-fold in 12.5 mM CTAC or CTAB, was dropcast onto the center of a dry GCE and allowed to completely dry for 30 min in a desiccator. The counter and reference electrodes were isolated from the reaction solution using bridge tubes with glass frits containing 100 mM sulfuric acid. The electrochemical cell was clamped above a stir plate (at room temperature or in a 40 °C water bath), and 10 mL of 100–150 mM CTAC or CTAB, 500 μL of 10 mM HAuCl_4_, 200 μL of 1 M HCl, and 0–100 μL of 2.5–10 mM AgNO_3_ were injected. The stir bar was added, and the solution was stirred at 300 rpm throughout the synthesis from this point. Bridge tubes and reference and counter electrodes were added, and electrode leads were connected to the potentiostat (Gamry Instruments 1010E). After stirring for 5 min, 50 μL of 10 mM AA was injected. The solution was stirred for an additional 90 s. Following this, the working electrode with dropcast, dried Au seeds was introduced to the growth solution and connected to the potentiostat. A hold current of 0 μA/cm^2^ was applied for 10 s, followed by a constant current of between −0.662 μA/cm^2^ and −0.866 μA/cm^2^ for 3600 s. Following the conclusion of the deposition, the working electrode was gently rinsed with 40 °C DI water and dried for electron microscopy analysis. The growth solution was immediately removed from the electrochemical cell, vortexed, and a 1 mL aliquot was analyzed with UV–vis spectroscopy. A factor of +0.65 V was used to convert potentials from Hg/Hg_2_SO_4_ to the SHE scale. See Table S5 for specific experimental conditions (seed dilution factor, [surfactant], [AgNO_3_], and current density) for each shaped deposition condition.

### Potentiostatic Electrodeposition of (Ag)Au Nanoparticles

Potentiostatic deposition of (Ag)Au nanoparticles was conducted analogously to the galvanostatic electrodeposition procedure described above, except that +0.44 V or +0.46 V vs SHE potential was applied for 3600 s, rather than applying current. Potentiostatic electrodeposition was conducted with 1 μL of undiluted Au-CTAC seeds dropcast and dried on the working electrode, in a growth solution containing 10 mL 100 mM CTAC, 500 μL of 10 mM HAuCl_4_, 200 μL of 1 M HCl, 100 μL of 10 mM AgNO3, and 50 μL of 100 mM AA. A factor of +0.65 V was used to convert potentials from Hg/Hg_2_SO_4_ to the SHE scale.

### Anodic Stripping Voltammetry (ASV) of Electrodeposited (Ag)­Au Nanoparticles

Electrodeposited (Ag)Au CC samples on GCEs were washed with three droplets of 50 °C water and dried in a desiccator. This procedure was repeated three additional times. Electrodes were then washed with several droplets of warm methanol and dried again in a desiccator. The repetition of the washing procedure and washing with methanol was necessary to remove surfactant from the particle surface, which blocked stripping; washing with 50 °C water one time was inadequate (see Figures S14–S16). All ASVs were performed using a Gamry 1010E potentiostat in a five-port glass electrochemical cell (Gamry Instruments Dr. Bob’s Cell) with a GC rod as the counter electrode (Gamry Instruments), and a saturated Hg/Hg_2_SO_4_ reference electrode (Koslow Scientific). Following washing, ASV was performed using procedures adapted from the literature.^45^ For Ag stripping, which was conducted first, the potential was swept from 0.2 to 1.8 V vs SHE (optimization experiments) or 0.2 to 1.5 V vs SHE (experiments for ASV peak integration) in 10 mL of a 500 mM H_2_SO_4_ solution at a scan rate of 30 mV/s. 1.5 to 1.8 V vs SHE was omitted from ASVs used for quantitative analysis of the Ag:Au ratio following optimization of stripping conditions, as water splitting in that potential range during Ag stripping might affect the Au stripping. This was repeated four additional times for a total of five Ag stripping steps, to ensure total stripping of Ag and prevent formation of AgBr during the Au stripping, although no stripping peak current was observed after the first Ag stripping. The same electrode was then rinsed with water and dried in a desiccator before ASV was performed again in 10 mL of a 100 mM KClO_4_ and 10 mM KBr solution using a potential sweep of 0.2 to 1.8 V vs SHE (optimization experiments) or 0.2 to 1.5 V vs SHE (experiments for ASV peak integration) at a scan rate of 30 mV/s to strip Au. A factor of +0.65 V was used to convert potentials from Hg/Hg_2_SO_4_ to the SHE scale. This process was conducted analogously for stripping of monometallic Au nanoparticles, except that stripping was only conducted in a 100 mM KClO_4_ plus 10 mM KBr solution.

ASV of colloidal (Ag)Au CC samples was conducted analogously to ASV of electrodeposited samples, except for the preparation of the nanoparticle sample on the GCE. Colloidal (Ag)Au CC were synthesized using typical methods (see above). Following the completion of growth (>3 h), a 1 mL aliquot of (Ag)Au CC colloid was removed and transferred to a microcentrifuge tube. The solution was then centrifuged (Eppendorf 5430) at 6000 rpm for 4 min to spin down a pellet. The supernatant was removed with a Pasteur pipet, and the microcentrifuge tube refilled to 1 mL with DI water, vortexed, sonicated, vortexed, and then centrifuged again at 6000 rpm for 4 min. This procedure was repeated four additional times for a total of five washing steps. After the fifth wash, the supernatant was removed again, and the particle pellet was resuspended in 600 μL DI water and 600 μL methanol. One μL of this suspension was dropcast on a GCE and dried in a desiccator. After drying, the electrode was carefully washed with several droplets of warm methanol and dried again in the desiccator. Following this process, ASV was conducted.

Gamry Echem Analyst 2 and MATLAB Curve Fitter were used to analyze ASV data. Fitting of ASV peaks was necessary due to overlaps between the Au stripping and water splitting peaks. Ag stripping data were analyzed by subtracting the ASV from a blank electrode in an analogous solution, fitting a one-term Gaussian curve to the ASV peak of interest, integrating under this Gaussian fit across the Ag stripping peak potentials (0.9 to 1.1 V vs SHE), and dividing by the scan rate (30 mV/s) to obtain the charge passed during the stripping. Au stripping data were analyzed by subtracting the ASV from a blank electrode in an analogous solution, fitting a two-term Gaussian curve (with one term accounting for the Au stripping and one for the water splitting peak to the ASV peak of interest), integrating under the Gaussian fit from 0.95 to 1.35 V vs SHE, and dividing by the scan rate (30 mV/s) to obtain the charge passed during the stripping. Gaussian fits and R^2^ values can be found in Tables S1–S2. Prior to comparing Ag and Au stripping peaks, the charge calculated from the Au stripping peak was divided by three,^45^ as Au stripping occurs primarily via eq 1 (a three-electron oxidation), with limited or no contribution under Au stripping conditions from eq 2 (a one-electron oxidation),^53^ due to the latter’s lower thermodynamic favorability.

1 $${\mathrm{Au}}^{0}+4{\mathrm{Br}}^{-}\to {\mathrm{AuBr}}_{4}^{-}+3e^{-}\;\left(E^{0}=0.85\;\mathrm{V vs SHE}\right)$$

2 $${\mathrm{Au}}^{0}+2{\mathrm{Br}}^{-}\to {\mathrm{AuBr}}_{4}^{-}+1e^{-}\;\left(E^{0}=0.96\;\mathrm{V vs SHE}\right)$$

In contrast, Ag stripping occurs via a one-electron pathway.^45^ The charge obtained by integrating the Ag stripping peak was divided by 1/3 of the charge obtained by integrating the Au stripping peak to obtain an Ag:Au ratio.

### Considerations and Cleaning for Glassware and Electrochemical Apparatus

Au seeds, all stock solutions, and all growth solutions (except for those prepared in a heart-shaped electrochemical flask for OCP measurement) were prepared in 20 mL glass scintillation vials (DWK Life Sciences 986560; Wheaton Glass 20 mL Scintillation Vials: Polypropylene Caps, Foamed Polyethylene Liner). (Ag)Au syntheses are sensitive to the use of other lines of glass scintillation vials or plastic vials, and the expected shape development did not occur when other vials were used.

Electrochemistry glassware, including 4- or 5-neck heart-shaped flasks, bridge tubes for reference electrodes, miniature stir bars (3 mm × 10 mm), glass stoppers, and Teflon holders for glassy carbon disk electrode inserts were cleaned with aqua regia (1:3 ratio of concentrated nitric acid: concentrated hydrochloric acid; **caution: strong acid**) between each use. Porous glass frits on bridge tubes were replaced every ∼6 weeks or whenever they became permanently discolored. Electrochemistry flasks that were used for syntheses which contain chloride only vs chloride and bromide were kept separate, as adsorbed halides were challenging to remove from the glassware.

### Nanoparticle Characterization by Electron Microscopy

Electrodeposited particles on GCEs were washed 3–4 times with 40 °C DI water and placed in a custom metal sample holder to dry for at least 30 min before characterization by scanning electron microscopy (SEM; FEI Quanta 650 FE-SEM).

To prepare colloidal samples for characterization via SEM, the completed reaction vial was vortexed, sonicated briefly (around 30 s), and vortexed again. From this dark, cloudy solution, 1 mL was removed and transferred to a microcentrifuge tube. The solution was then centrifuged (Eppendorf 5430) at 6000 rpm for 4 min to spin down a pellet. The supernatant was removed with a Pasteur pipet, and the microcentrifuge tube refilled to 1 mL with DI water, vortexed, sonicated, vortexed, and then centrifuged again at 6000 rpm for 4 min. The supernatant was removed again, and a minimal amount of DI water (200–400 μL) was added, and the tube was sonicated and vortexed to disperse the particles. One μL of this solution was then dropcast onto a prepared silicon wafer and allowed to dry for at least 45 min in a desiccator before characterization by SEM (FEI Quanta 650 FE-SEM).

Nanoparticles were imaged within 72 h of sample preparation. Average particle shape was determined using >100 particles. When triplicate samples were prepared, particle size and shape were determined using >100 particles across images of all three samples. Particle surface coverage density was determined by counting the number of particles per image in 10 images of identical low magnification taken at different locations on the electrode. Electrode area in each image was determined using ImageJ. Brightness and contrast of electron microscopy images were normalized using the Curves adjustment tool in Adobe Photoshop.

### Growth Solution Characterization by UV–visible Spectroscopy

Following the completion of electrodeposition, the growth solution was vortexed, and a 1 mL aliquot was analyzed using UV–vis spectroscopy (Cary 60 UV–vis spectrophotometer). UV–vis characterization was done within 15 min of electrodeposition reaction completion.

## Supplementary Material



## Notes

Replication data repository, additional discussion of anodic stripping voltammetry experiments, plots of UV–vis spectroscopy, additional SEM images, additional graphs of OCP and chronopotentiometry profiles, plots of anodic stripping voltammograms, tables of anodic stripping voltammetry data and fitting parameters, table of nanoparticle shape distributions, table of optimized electrodeposition conditions (PDF)
