# Supplementary material for: Synergistic Tuning of Diffusion Enables Surface-Passivation-Directed Electrodeposition of Shaped Bimetallic Nanoparticles
Source: ACS Nano. 2026 Oct 25;20(44):30011–24. doi: 10.1021/acsnano.6c13712 (PMC13758392; doi:10.1021/acsnano.6c13712)
Supplement: Supplementary file 1 [file nn6c13712_si_001.pdf]

Supporting Information for

Synergistic Tuning of Diffusion Enables Surface-Passivation-Directed Electrodeposition of Shaped Bimetallic Nanoparticles

Gabriel C. Halford,[a] Abigail M. Sublett,[a] Adam J. Buratowski,[a] Kaelyn M. Harmon,[b] Harikrishnan N. Nambiar,[a] and Michelle L. Personick*[a]

[a] Department of Chemistry, University of Virginia, Charlottesville, Virginia 22904, United States

[b] Department of Materials Science and Engineering, University of Virginia, Charlottesville, Virginia 22904, United States

* Email: mpersonick@virginia.edu

Replication Data

Halford, Gabriel C.; Sublett, Abigail M.; Buratowski, Adam J.; Harmon, Kaelyn M.; N. Nambiar, Harikrishnan; Personick, Michelle L., 2026, "Replication Data for: Synergistic Tuning of Diffusion Enables Surface-Passivation-Directed Electrodeposition of Shaped Bimetallic Nanoparticles," https://doi.org/10.18130/V3/ACBYOE, University of Virginia Dataverse.

Details of Anodic Stripping Voltammetry (ASV) Optimization and Analysis

Anodic stripping voltammetry (ASV) was used to probe the presence, nature, and amount of Ag coverage on the electrodeposited (Ag)Au CC deposited on 500-fold diluted Au seeds (Figure S14-S18 and Table S1-S3). Following (Ag)Au CC electrodeposition, Ag was stripped in 500 mM H_2SO_4, and, next, Au was stripped in a mixture of 100 mM $KClO_4$ plus 10 mM KBr, consistent with other literature procedures for ASV of AgAu bimetallic nanoparticles.[1] To correctly interpret these results, appropriate cleaning of residual surfactant off the nanoparticle surface was necessary (see Methods in the main text). When more minimal washing (similar to SEM sample preparation) was conducted, the ASV peak for Ag was shifted to an atypically positive value compared to other literature and the peak was very broad (Figure S14),[2–4] while the peak potential (E_p) for the Au stripping peak was typical of bulk Au stripping.[4] Though ASV peak potentials can have a

dependance on nanoparticle size,[5,6] the stripping peak potentials approach the values for the bulk metals for particle diameters >40 nm.[7] We attribute the unusual position of the Ag peak but not the Au peak in stripping of (Ag)Au CC to the fact that Ag stripping must be conducted first, as a similar peak shift for the first Au stripping was noted when monometallic Au nanoparticles (see Figure S20) were electrodeposited and then ASV was only taken in a 100 mM $KClO_4$ plus 10 mM KBr mixture (Figure S15). When a more rigorous five-step washing procedure with warm water and methanol was used, the Au stripping peak for stripping of electrodeposited monometallic Au nanoparticles was consistent with the Au stripping peak obtained for stripping of (Ag)Au CC (following Ag stripping) and with literature (Figure S15). The decrease in peak height, peak broadening, and shift of the oxidative ASV peaks to more positive potentials with excess CTAC present on the particle surface is consistent with other reports of the effects of excess surface-bound surfactant[2,8,9] or other surface capping agents[10] on ASV, where these species make oxidation more difficult by "blocking" the particle surface. The more rigorous washing procedure was confirmed to not significantly remove (Ag)Au particles from the electrode surface (Figure S16), and it was conducted prior to anodic stripping of Ag and Au from (Ag)Au CC (Figure S17). The presence of a peak during the Ag stripping—not noted on a control with a bare electrode in 500 mM H_2SO_4—confirmed the presence of Ag in the CC particles, while the E_p (~1.0 V vs. SHE) is consistent with the literature value (0.95 to 1.1 V vs. SHE) for a submonolayer coverage of underpotential deposited metallic Ag on Au (Figure S17).[2] Of note, the expected E_p for a full monolayer coverage of Ag on Au, a bilayer shell coverage of Ag on Au, or bulk Ag deposition are all significantly more negative (0.68 V vs. SHE, 0.62 V vs. SHE, and 0.5-0.6 V vs. SHE, respectively).[2–4] Ag and Au stripping peaks were fit with Gaussian profiles (to allow for the deconvolution of Au stripping and water splitting peaks), and these were integrated to obtain ratios

of their charge, from which a ratio of 0.011±0.001 Ag:Au was calculated (see Methods for additional detail). Analogous ASV experiments were conducted for stripping of well-washed, diluted colloidal (Ag)Au particles that were dropcast and dried on a glassy carbon electrode. Similar Ag and Au stripping peak positions (±30 mV) were observed to the ASVs of the electrodeposited particles (Figure S18). While the peak current was ~1 order of magnitude higher for Ag and Au stripping with colloidal particles than with electrodeposited particles, likely due to a higher number of dropcast colloidal particles present on the electrode, the ratio of Ag:Au determined via peak integrals was in reasonable agreement (0.012±0.002) to the ratio calculated from ASV of electrodeposited samples. Both ratios are roughly consistent with the ~0.012 Ag:Au ratio obtained by ICP-OES spectroscopy for colloidal (Ag)Au particles of a similar size.[11]

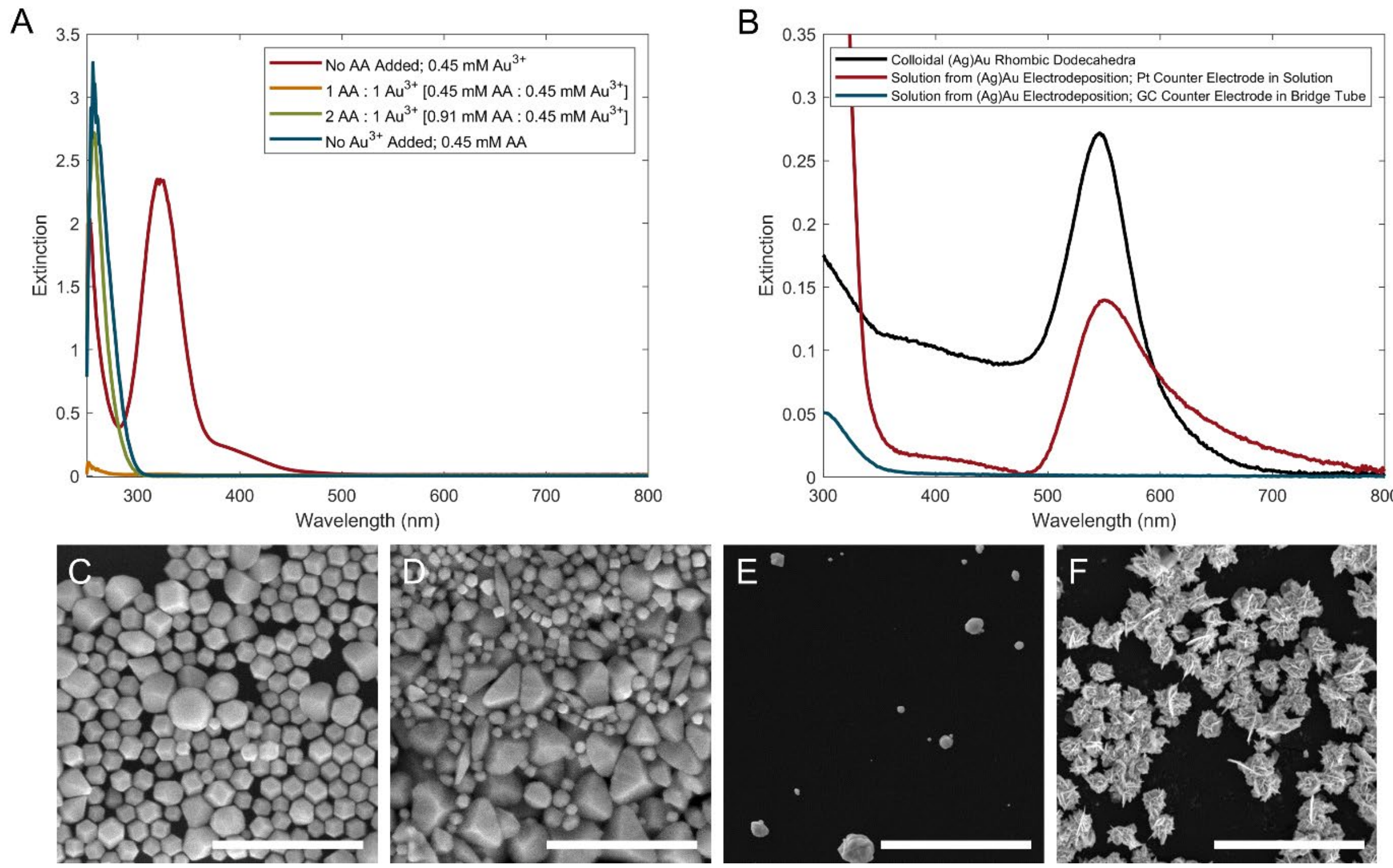

Figure S1. (A) UV-vis spectra of aqueous solutions of 0.45 mM $HAuCl_4$ (without AA); 0.45 mM AA and 0.45 mM $HAuCl_4$ (1 AA : 1 Au^{3+}); 0.91 mM AA and 0.45 mM $HAuCl_4$ (2 AA : 1 Au^{3+}); and 0.45 mM AA (no Au^{3+}). All solutions also contained 100 mM CTAC and 18.2 mM HCl. (B) UV-vis spectra of a representative suspension of as-synthesized (Ag)Au rhombic dodecahedral nanoparticles in 100 mM CTAC; a representative growth solution following an electrodeposition synthesis of (Ag)Au nanoparticles (-0.815 $\mu A/cm^2$ applied for 60 minutes) with a Pt wire counter electrode inserted directly into solution, demonstrating solution-phase growth of (Ag)Au nanoparticles due to chemical reduction; and a representative growth solution following an electrodeposition synthesis of (Ag)Au nanoparticles (-0.815 $\mu A/cm^2$ applied for 60 minutes) with a glassy carbon counter electrode isolated in a bridge tube. The disappearance of the broad localized surface plasmon resonance (LSPR) peak around 550 indicates a lack of solution-phase nanoparticle growth when the counter electrode is isolated. (C-D) SEM images of colloidal (Ag)Au nanoparticles formed in solution when galvanostatic electrodeposition was performed with a Pt wire counter electrode inserted directly into the growth solution. (E-F) SEM images of nanoparticles formed chemically on an (E) unseeded or (F) seeded glassy carbon working electrode immersed overnight in a (Ag)Au nanoparticle growth solution along with a Pt wire. Note that no current or potential was applied. Scale bars for (C-D): 500 nm. Scale bars for (E-F): 5 µm.

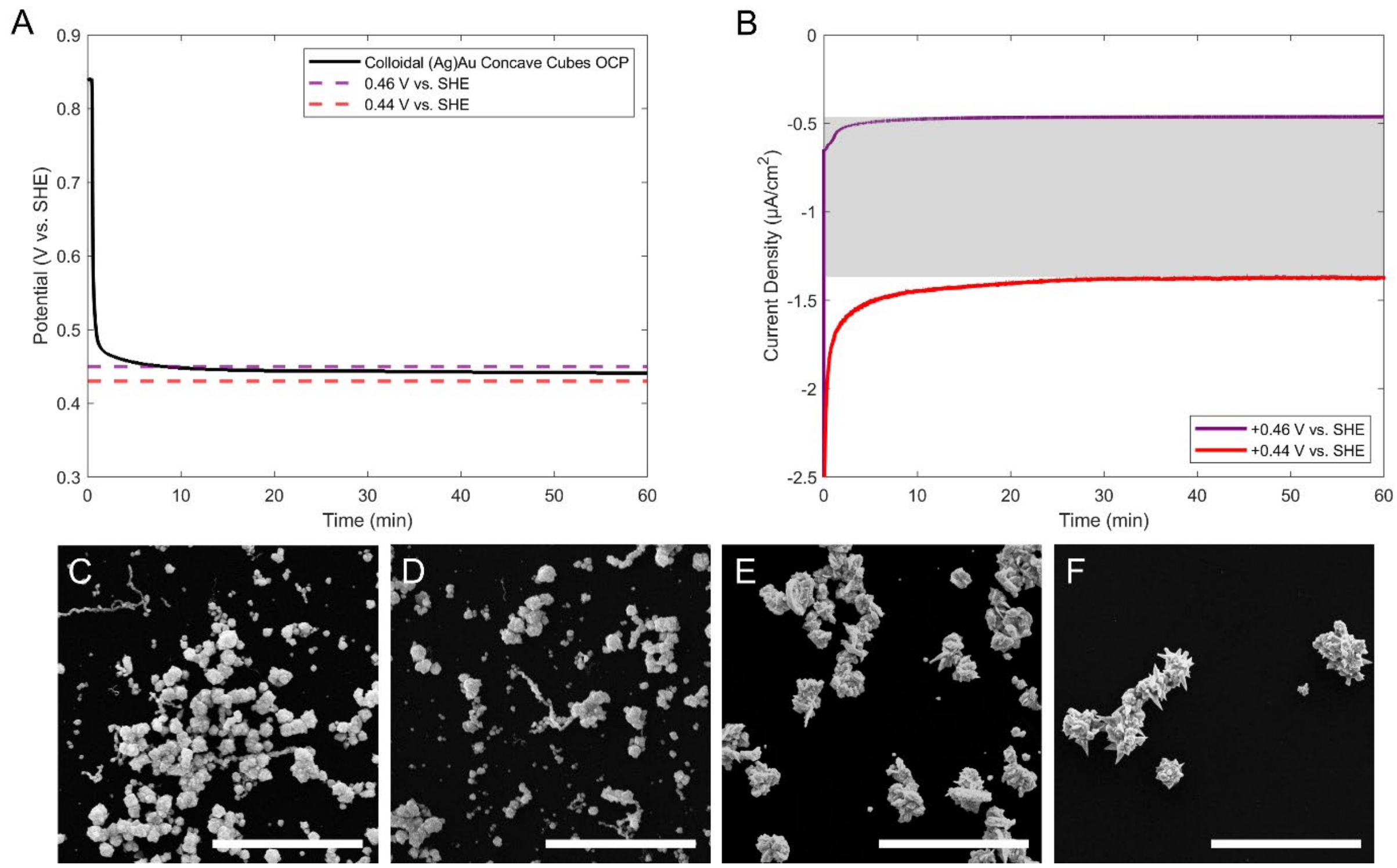

Figure S2. (A) Comparison between OCP measurement of colloidal (Ag)Au nanoparticle synthesis and applied potentials for chronoamperometry experiments. (B) Chronoamperometry traces for potentiostatic electrodeposition of (Ag)Au nanoparticles in 100 mM CTAC with 92.0 μM Ag^+ using either + 0.46 V vs. SHE (purple) or + 0.44 V vs. SHE (red) applied potential for 60 minutes. The gray box represents the range of current densities (-0.45 μA/cm^2 and -1.35 μA/cm^2) between the steady-state portions of the two current transients, which provides an estimate of the region in which optimal current densities for galvanostatic (Ag)Au nanoparticle electrodeposition are likely to be found. (C-E) SEM images of (Ag)Au nanoparticles synthesized using (C-D) + 0.46 V vs. SHE or (E-F) + 0.44 V vs. SHE applied potential. Scale bars for (C-F): 1 μm. Black trace in (A) adapted with permission from ref. 12. Available under a Creative Commons CC-BY 4.0 license. Copyright 2026 Halford, Sublett, and Personick.

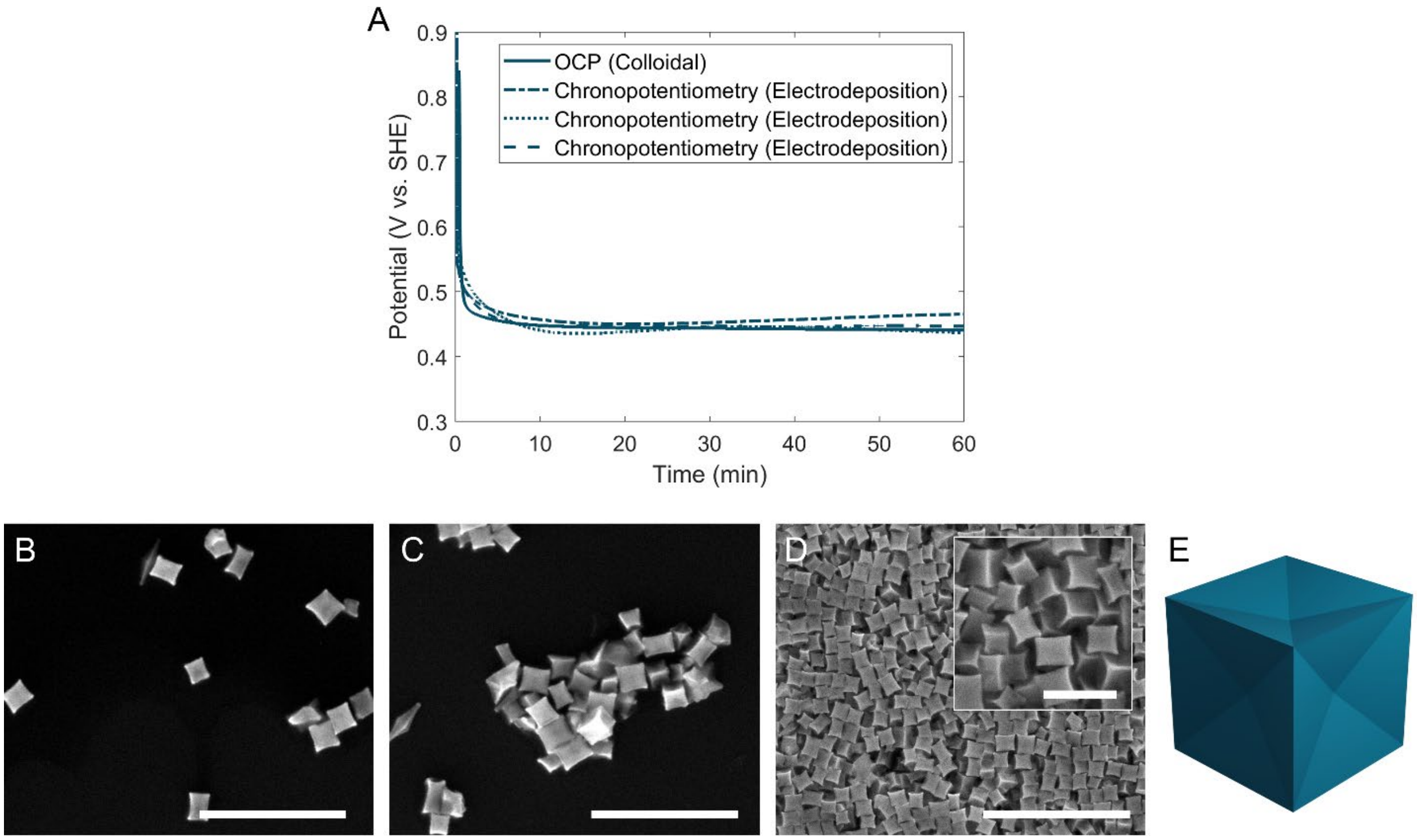

Figure S3. (A) Comparison between OCP measurement of colloidal CC growth (solid dark teal) and triplicate chronopotentiometry measurements (dashed dark teal) of galvanostatic electrodeposition of CC in 125 mM cetyltrimethylammonium chloride (CTAC) with 20.9 µM Ag^{+} and -0.815 µA/cm^2 applied current for 60 minutes, onto 200-fold diluted Au seeds. (B-C) Representative SEM images from triplicate electrodeposition experiments of electrodeposited (Ag)Au CC nanoparticles deposited onto Au seeds (200-fold diluted seed colloid; standard surface coverage). See Figure 1C-E. (D) SEM images of colloidal (Ag)Au CC nanoparticles for comparison. (E) Model of a CC. Scale bars: 1 µm (B-D) and 250 nm (D inset).

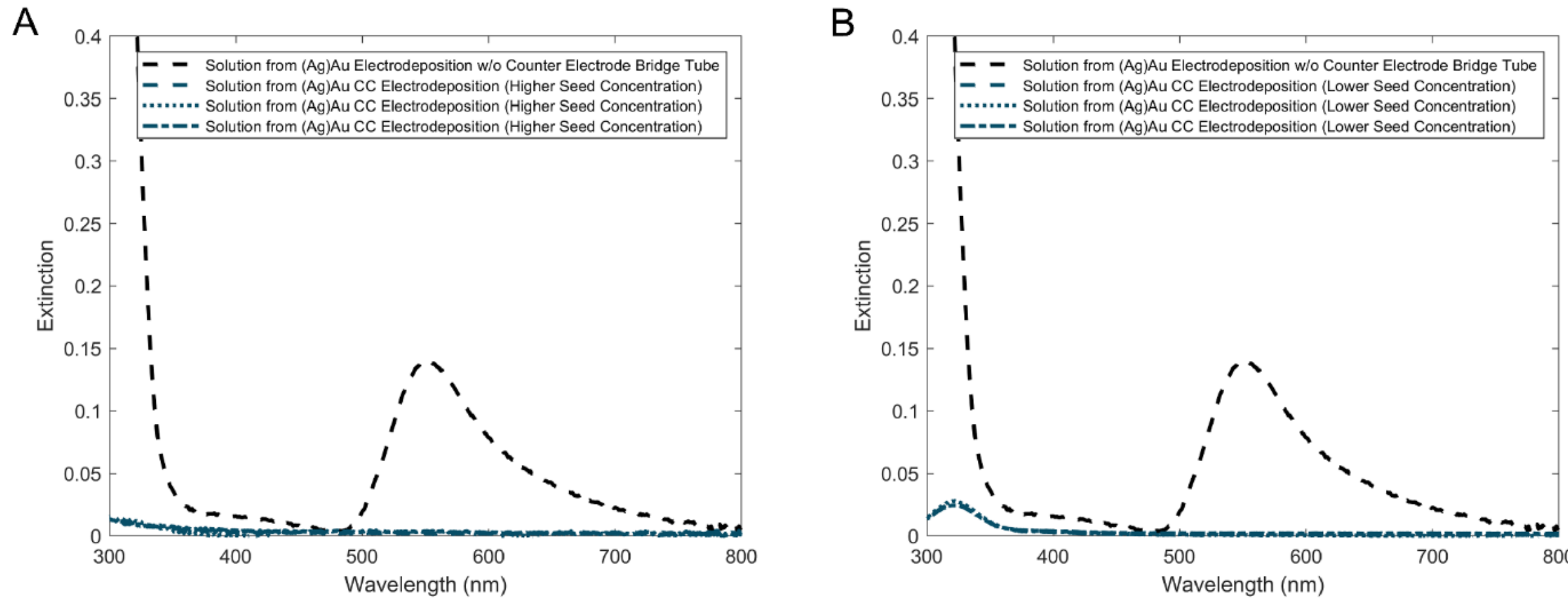

Figure S4. (A-B) UV-vis spectra of growth solutions following electrodeposition of (Ag)Au CC (-0.815 μA/cm^2 applied for 60 minutes) with (A) higher Au seed surface coverage (200-fold diluted seed colloid) and (B) lower Au seed surface coverage (500-fold diluted seed colloid), showing a lack of solution phase nanoparticle growth. A representative growth solution following an electrodeposition synthesis of (Ag)Au nanoparticles (-0.815 μA/cm^2 applied for 60 minutes) with a Pt wire counter electrode inserted directly into solution, in which solution-phase growth of (Ag)Au nanoparticles occurs due to chemical reduction, is provided for comparison.

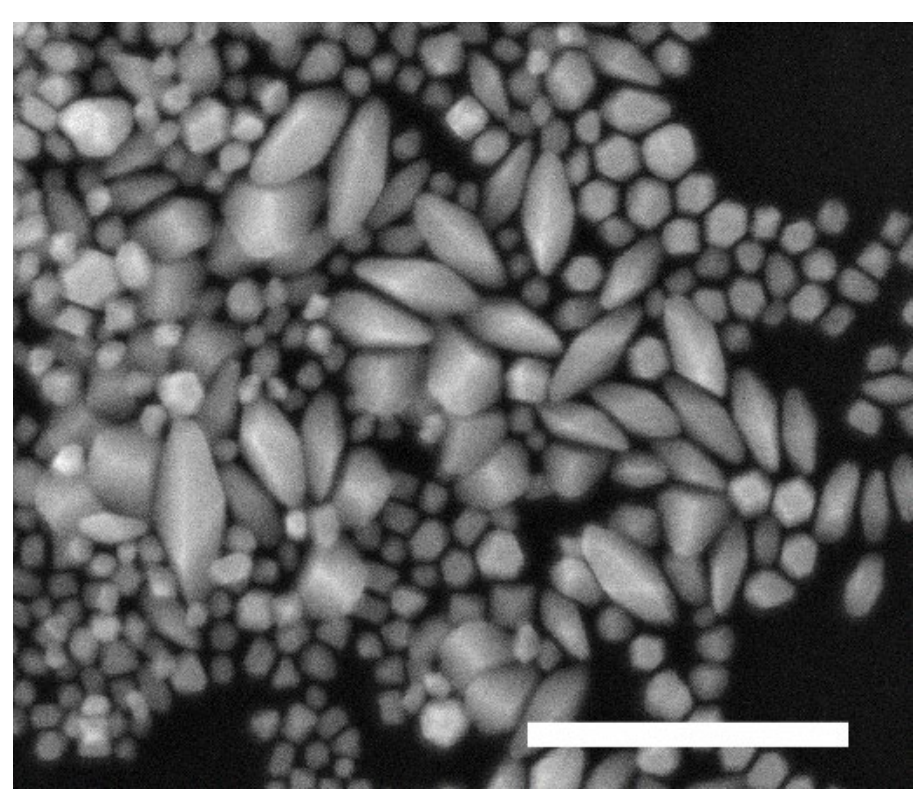

Figure S5. SEM image of colloidal (Ag)Au nanoparticles synthesized in 100 mM CTAC with 20.9 μM Ag^+ in the growth solution. Scale bar: 500 nm.

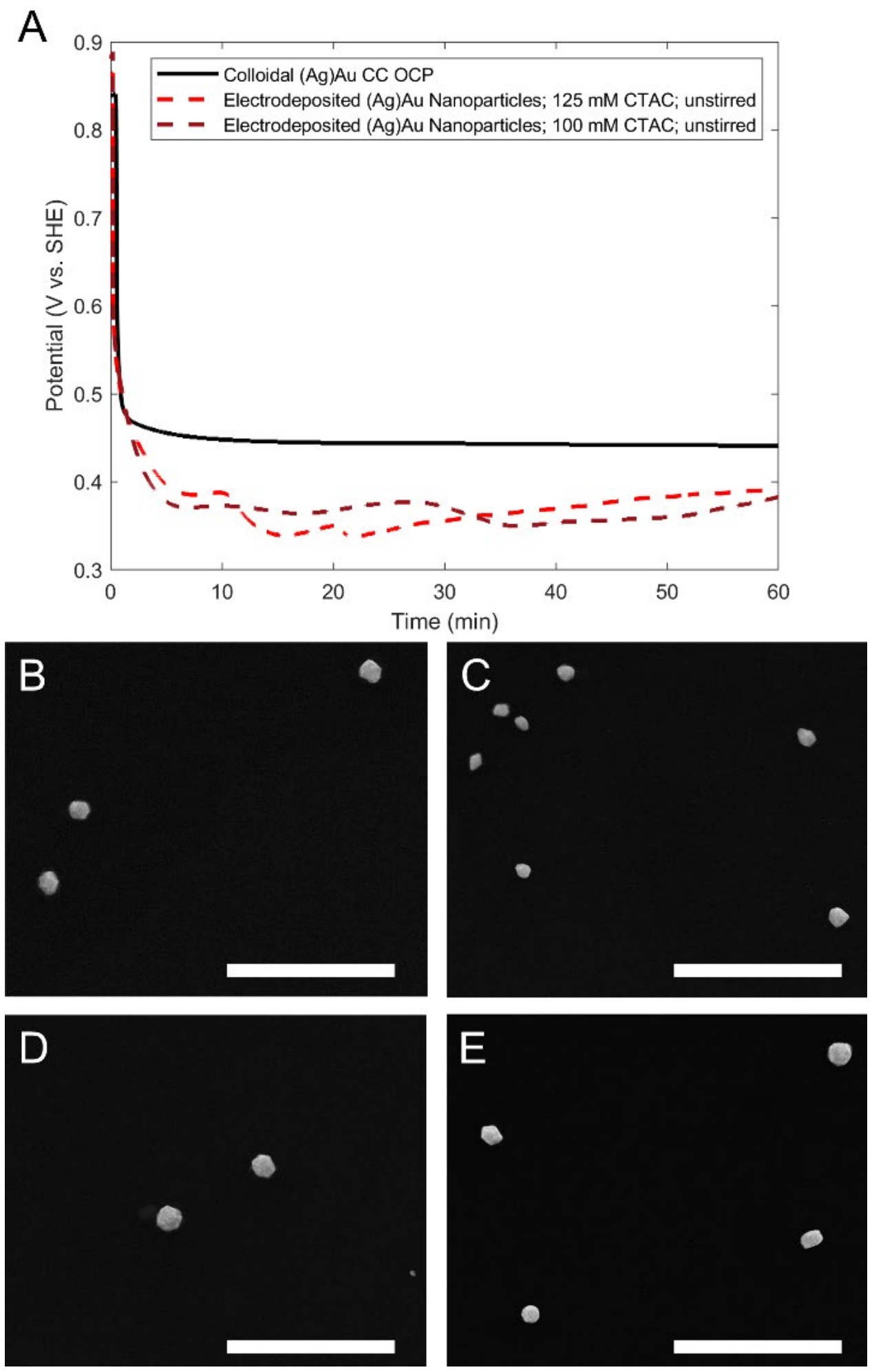

Figure S6. (A) Comparison between OCP measurement of colloidal (Ag)Au CC growth (black) and chronopotentiometry measurements of unstirred galvanostatic electrodeposition of (Ag)Au nanoparticles in 125 mM CTAC (dashed red) and 100 mM CTAC (dashed dark red) with 30.0 μM Ag^{+} and -0.815 $\mu A/cm^2$ applied current for 60 minutes, onto 500-fold diluted Au seeds. (B-E) SEM images of (Ag)Au nanoparticles electrodeposited without stirring in (B-C) 125 mM CTAC (red trace) and (D-E) 100 mM CTAC (dark red trace). Scale bars: 500 nm. Black trace in (A) adapted with permission from ref. 12. Available under a Creative Commons CC-BY 4.0 license. Copyright 2026 Halford, Sublett, and Personick.

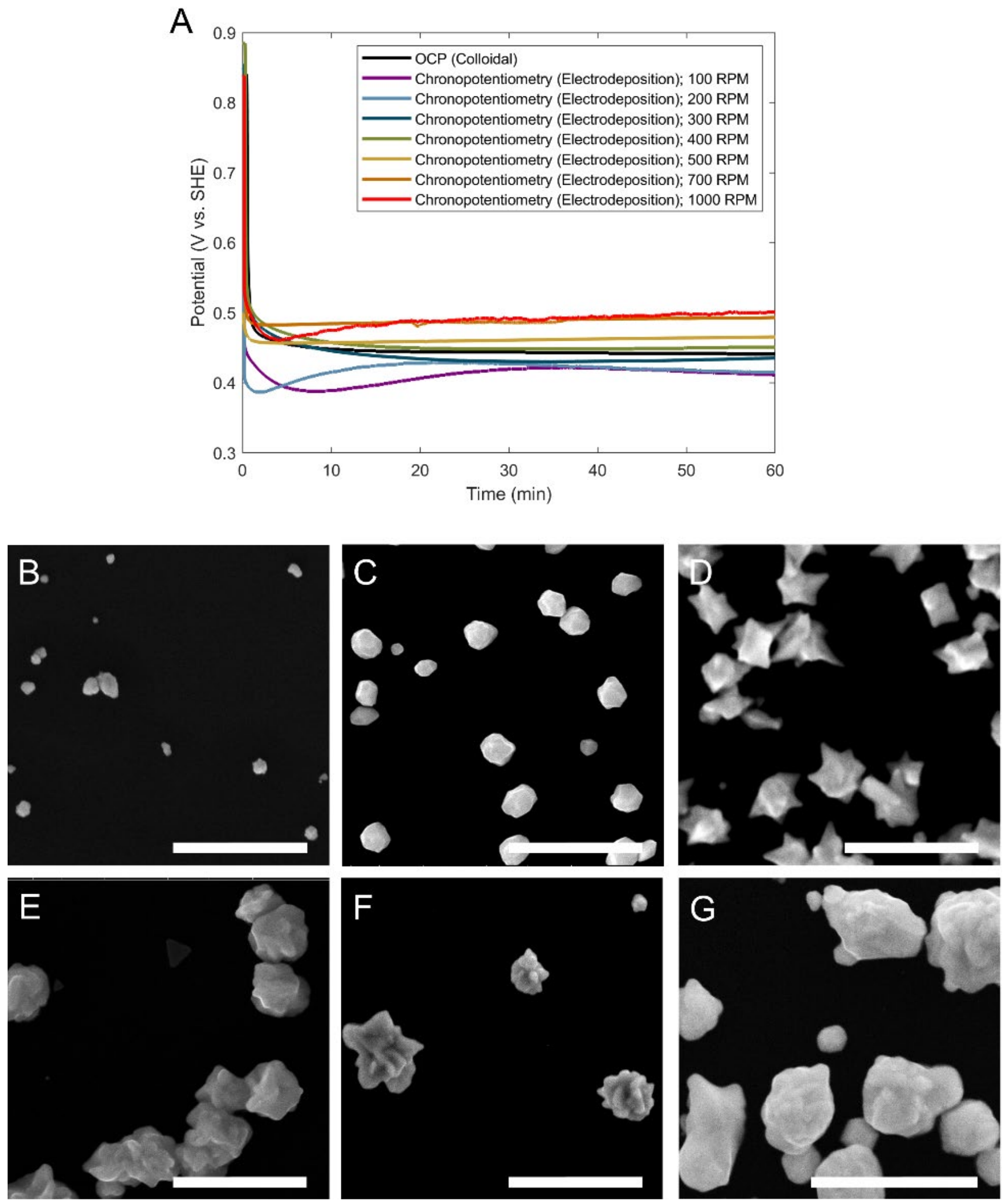

Figure S7. (A) Comparison between OCP measurement of colloidal (Ag)Au CC growth (black) and chronopotentiometry measurements of galvanostatic electrodeposition of (Ag)Au nanoparticles with varied mechanical stir rates, under otherwise standard, optimized conditions. 300 RPM (dark teal) is the standard stir rate. (B-G) SEM images of (Ag)Au nanoparticles electrodeposited with stir rates of (B) 100 RPM (purple trace), (C) 200 RPM (light blue trace), (D) 400 RPM (light green trace), (E) 500 RPM (yellow trace), (F) 700 RPM (orange trace) and (G) 1000 RPM (red trace). Scale bars: 500 nm. Black trace in (A) adapted with permission from ref. 12. Available under a Creative Commons CC-BY 4.0 license. Copyright 2026 Halford, Sublett, and Personick.

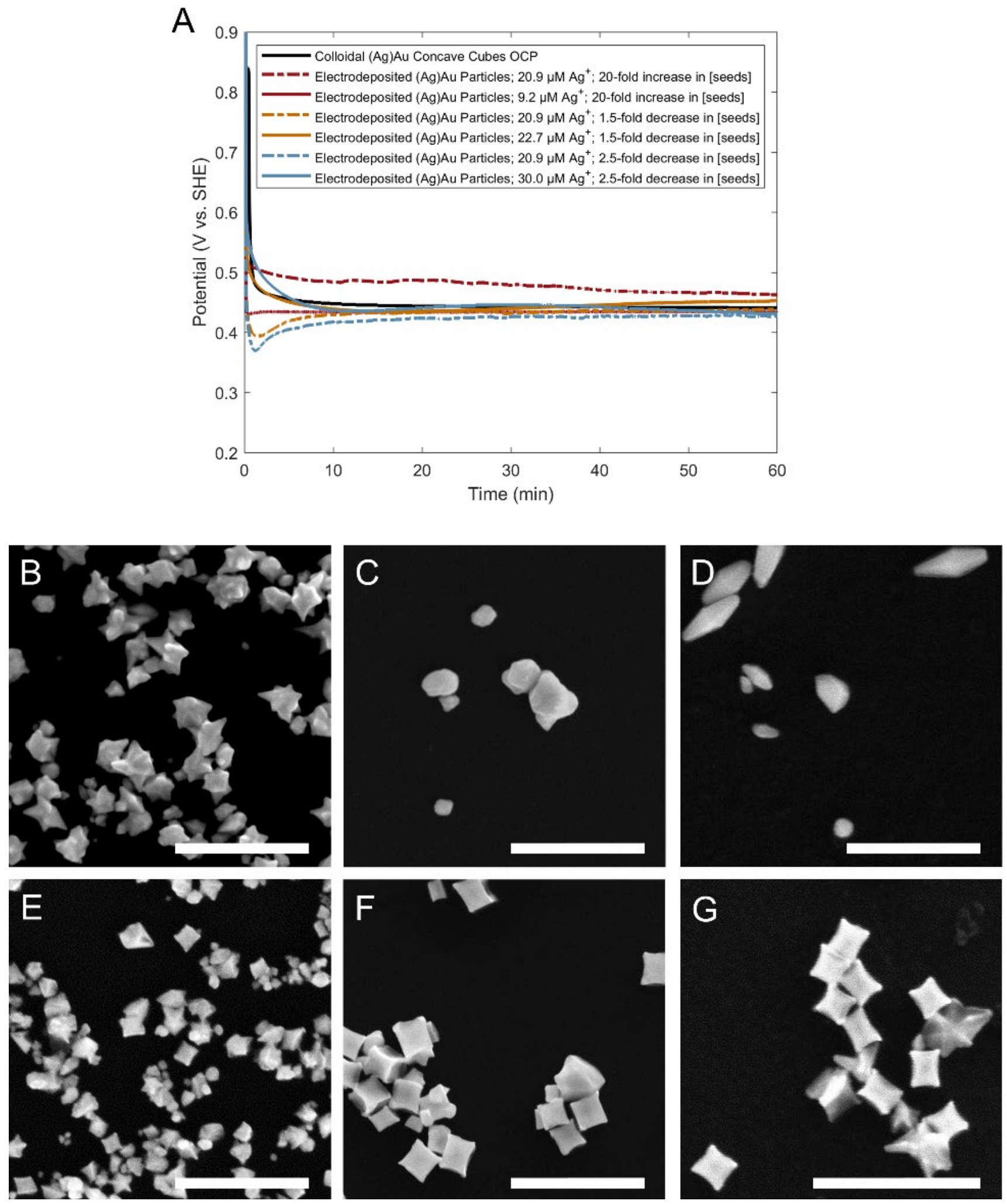

Figure S8. (A) Comparison between OCP measurement of colloidal (Ag)Au CC growth and chronopotentiometry measurements of galvanostatic electrodeposition of (Ag)Au nanoparticles with different concentrations of Au seed particles dropcast and dried on the working electrode. Dashed colored lines represent electrodeposition with 20.9 µM Ag^+ in the growth solution, while solid-colored lines represent electrodeposition with the optimal [Ag^+] for CC formation at each seed concentration: 9.2 µM Ag^+ for a 20-fold increase in seed concentration (relative to standard conditions), 22.7 µM Ag^+ for a 1.5-fold decrease in seed concentration, and 30.0 µM Ag^+ for 1.5-fold decrease in seed concentration. (B-G) SEM images of (Ag)Au nanoparticles electrodeposited with (B) 20.9 µM Ag^+ and 20-fold higher [seeds], (C) 20.9 µM Ag^+ and 1.5-fold lower [seeds], (D) 20.9 µM Ag^+ and 2.5-fold lower [seeds], (E) 9.2 µM Ag^+ and 20-fold higher [seeds], (F) 22.7 µM Ag^+ and 1.5-fold lower [seeds], and (G) 30.0 µM Ag^+ and 2.5-fold lower [seeds]. Scale bars: 1 µm. Black trace in (A) adapted with permission from ref. 12. Available under a Creative Commons CC-BY 4.0 license. Copyright 2026 Halford, Sublett, and Personick.

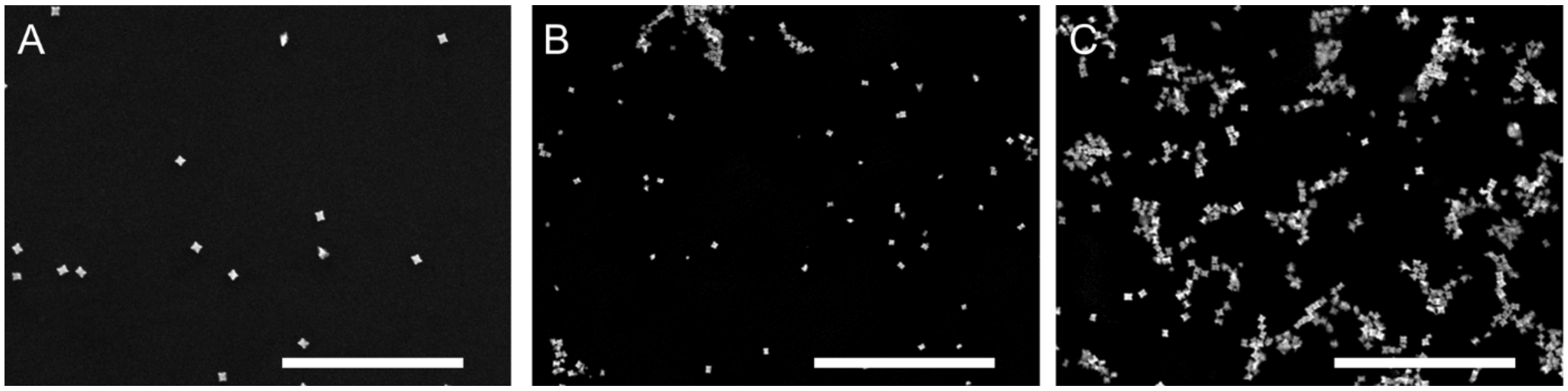

Figure S9. (A-C) Low-magnification SEM images showing representative electrodeposited (Ag)Au CC coverage in the center of the working electrode with 1 µL of different concentrations of Au seed colloid. These represent a (A) 2.5-fold decrease in seed colloid concentration relative to standard conditions, (B) 1.5-fold decrease in seed colloid concentration relative to standard conditions, or (C) standard concentration of Au seed particles dropcast and dried on the electrode. Scale bars: 5 µm.

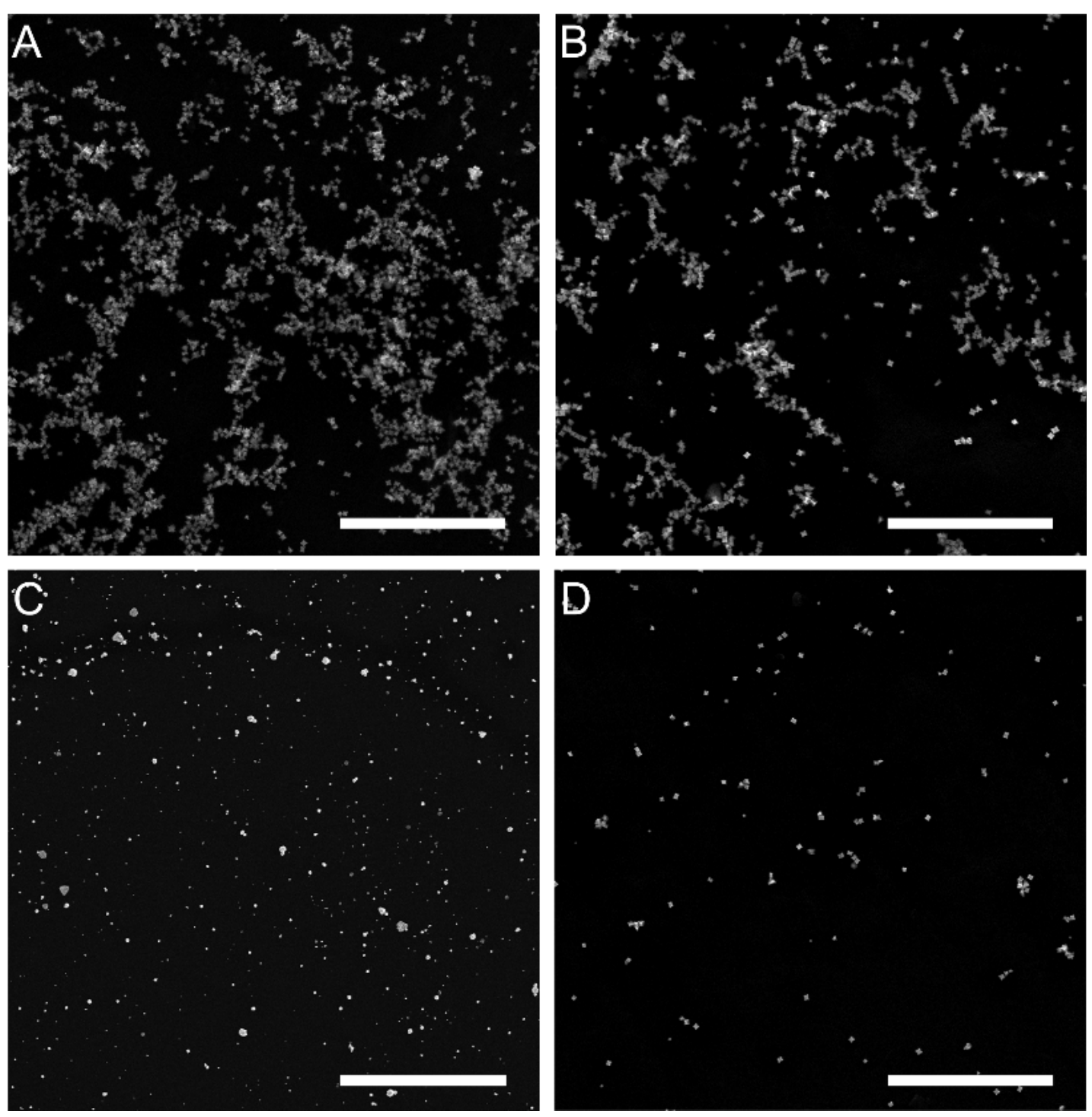

Figure S10. (A-D) Representative low-magnification SEM images used to determine particle surface density of electrodeposited (Ag)Au CC for different surface coverages of Au seeds, as referred to in the text. (A) 20-fold increase in seed colloid concentration; 39±25 particles per μm^2, (B) standard seed colloid concentration; 23±7 particles per μm^2, (C) 1.5-fold decrease in seed colloid concentration; 13±9 particles per μm^2, (D) 2.5-fold decrease in seed colloid concentration; 3±1 particles per μm^2. Scale bars: 10 µm.

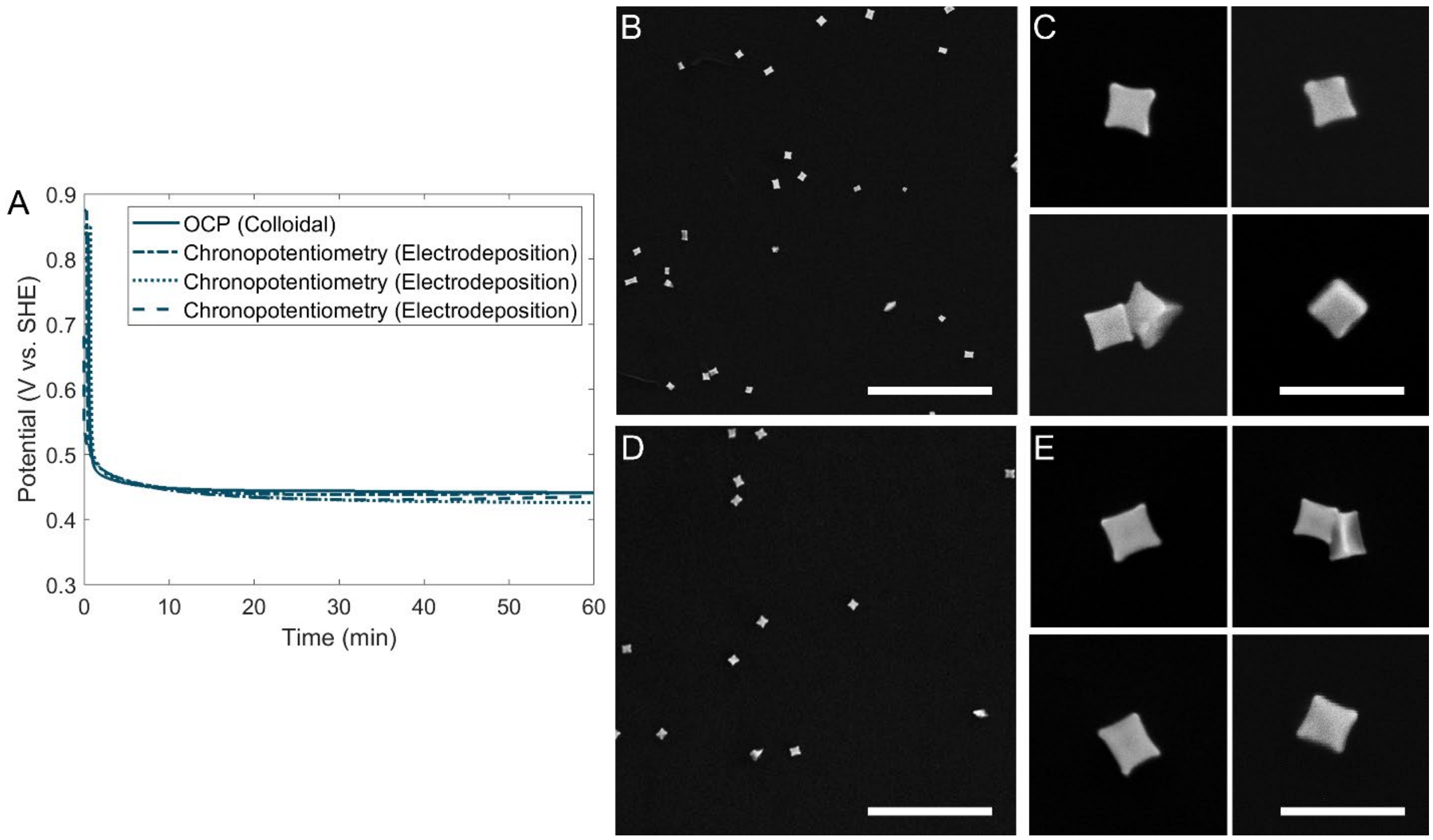

Figure S11. (A) Comparison between OCP measurement of colloidal CC growth (solid line) and triplicate chronopotentiometry measurements (dotted and/or dashed lines) of galvanostatic electrodeposition of CC in 125 mM cetyltrimethylammonium chloride (CTAC) with 20.9 µM Ag^+ and -0.815 µA/cm^2 applied current for 60 minutes, onto Au seeds (500-fold diluted seed colloid). (B-D) Representative SEM images of electrodeposited CC nanoparticles from triplicate electrodeposition experiments, showing (B,D) deposition density and (C,E) high-magnification images of individual CC particles. See Figure 1F-H for SEM images from the first of the triplicate electrodeposition experiments. Scale bars: 5 µm (B, D) and 500 nm (C, E).

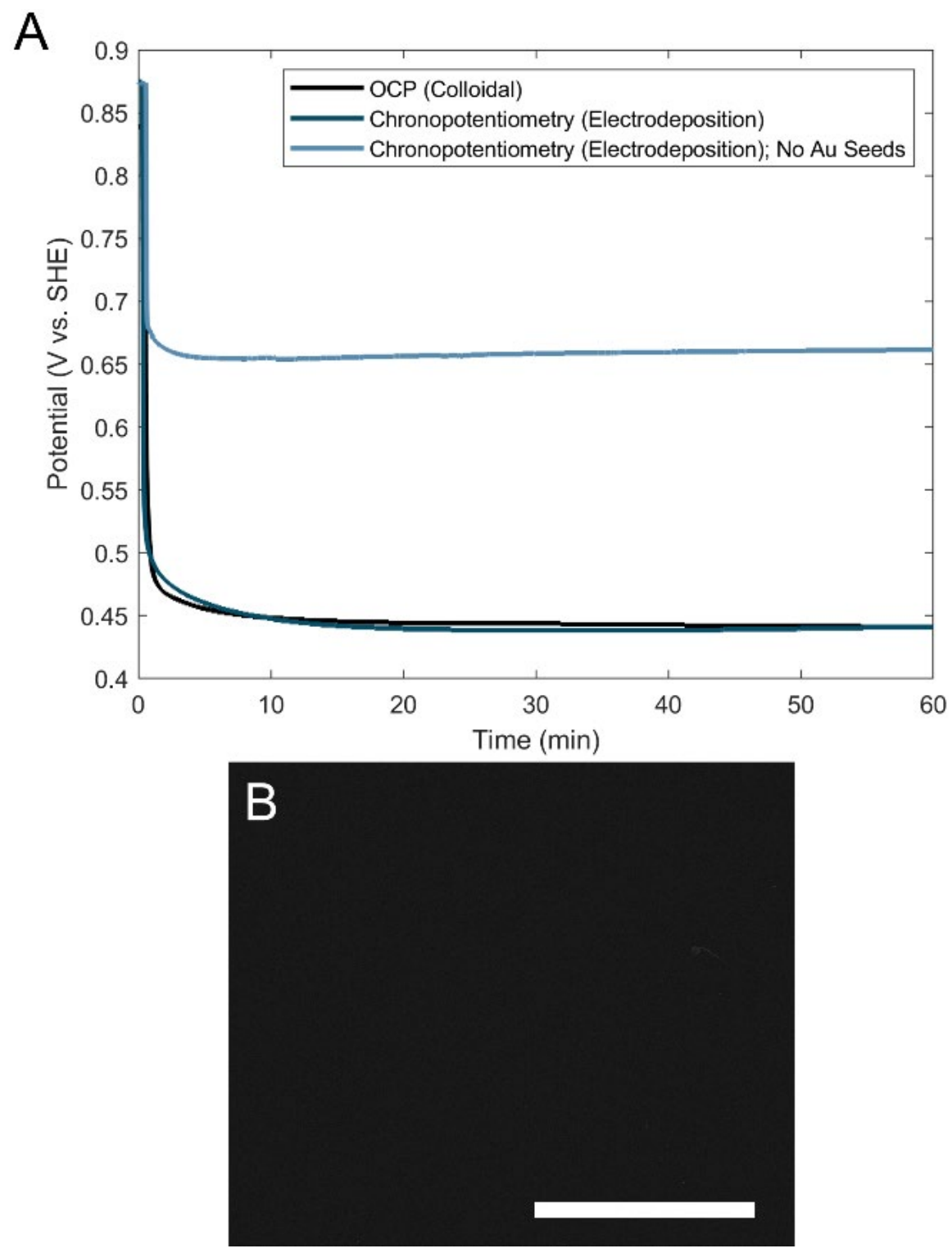

Figure S12. (A) Comparison between OCP measurement of colloidal (Ag)Au CC growth (black), chronopotentiometry measurement of standard galvanostatic electrodeposition of (Ag)Au CC onto 500x diluted Au seeds (dark teal), and chronopotentiometry measurement of an analogous (Ag)Au electrodeposition without Au seeds on the working electrode (light blue). (B) Low-magnification SEM image of the unseeded working electrode following electrodeposition under otherwise standard (Ag)Au CC deposition conditions (light blue), showing an absence of electrodeposited nanoparticles. Scale bar: 5 µm. Black trace in (A) adapted with permission from ref. 12. Available under a Creative Commons CC-BY 4.0 license. Copyright 2026 Halford, Sublett, and Personick.

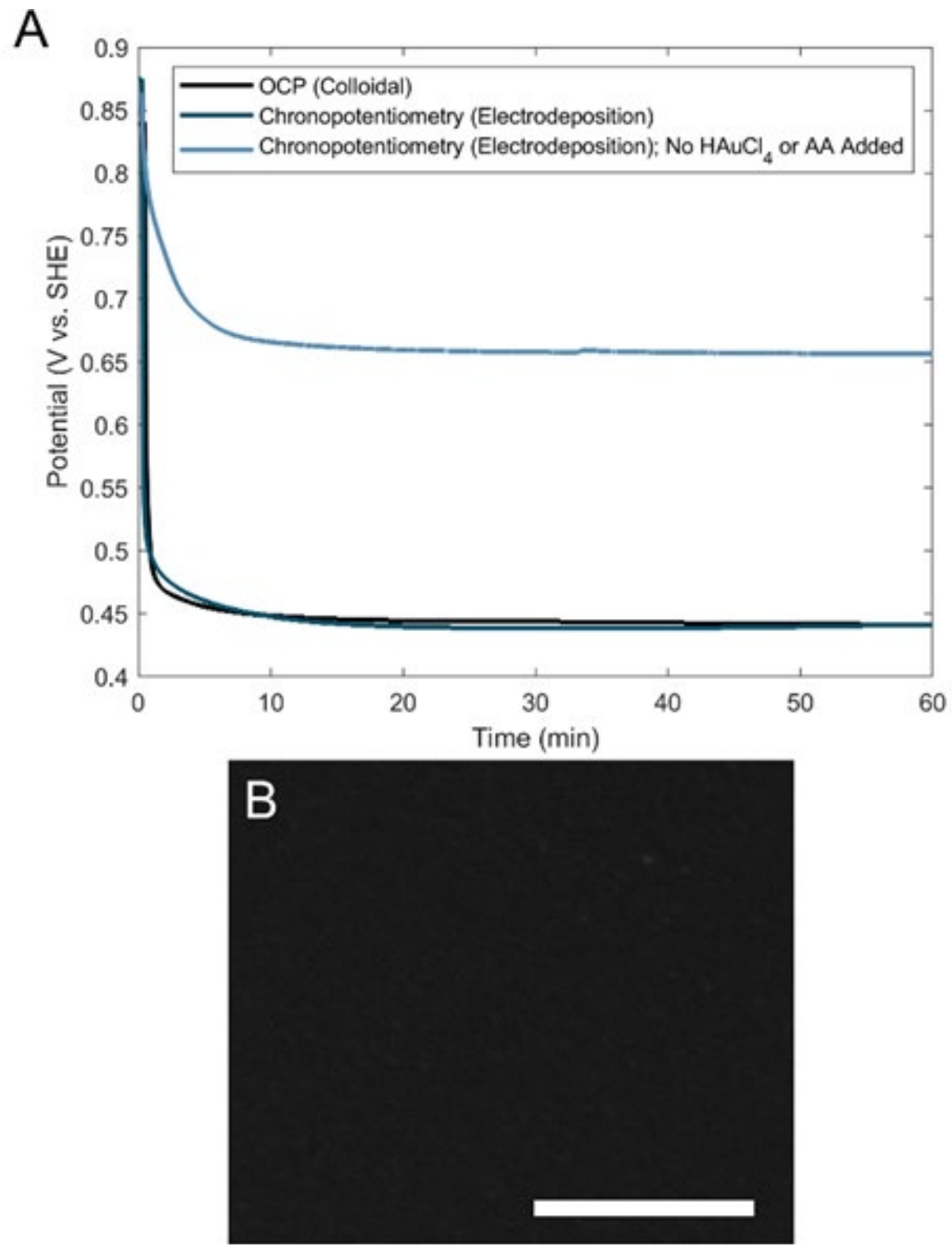

Figure S13. (A) Comparison between OCP measurement of colloidal (Ag)Au CC growth (black), chronopotentiometry measurement of standard galvanostatic electrodeposition of (Ag)Au CC onto 500x diluted Au seeds (dark teal), and chronopotentiometry measurement of an analogous (Ag)Au electrodeposition without $HAuCl_4$ or stoichiometric AA added to the growth solution (light blue). (B) Low-magnification SEM image of the working electrode following electrodeposition without $HAuCl_4$ or stoichiometric AA added to the growth solution (light blue), showing an absence of electrodeposited nanoparticles. Scale bar: 5 µm. Black trace in (A) adapted with permission from ref. 12. Available under a Creative Commons CC-BY 4.0 license. Copyright 2026 Halford, Sublett, and Personick.

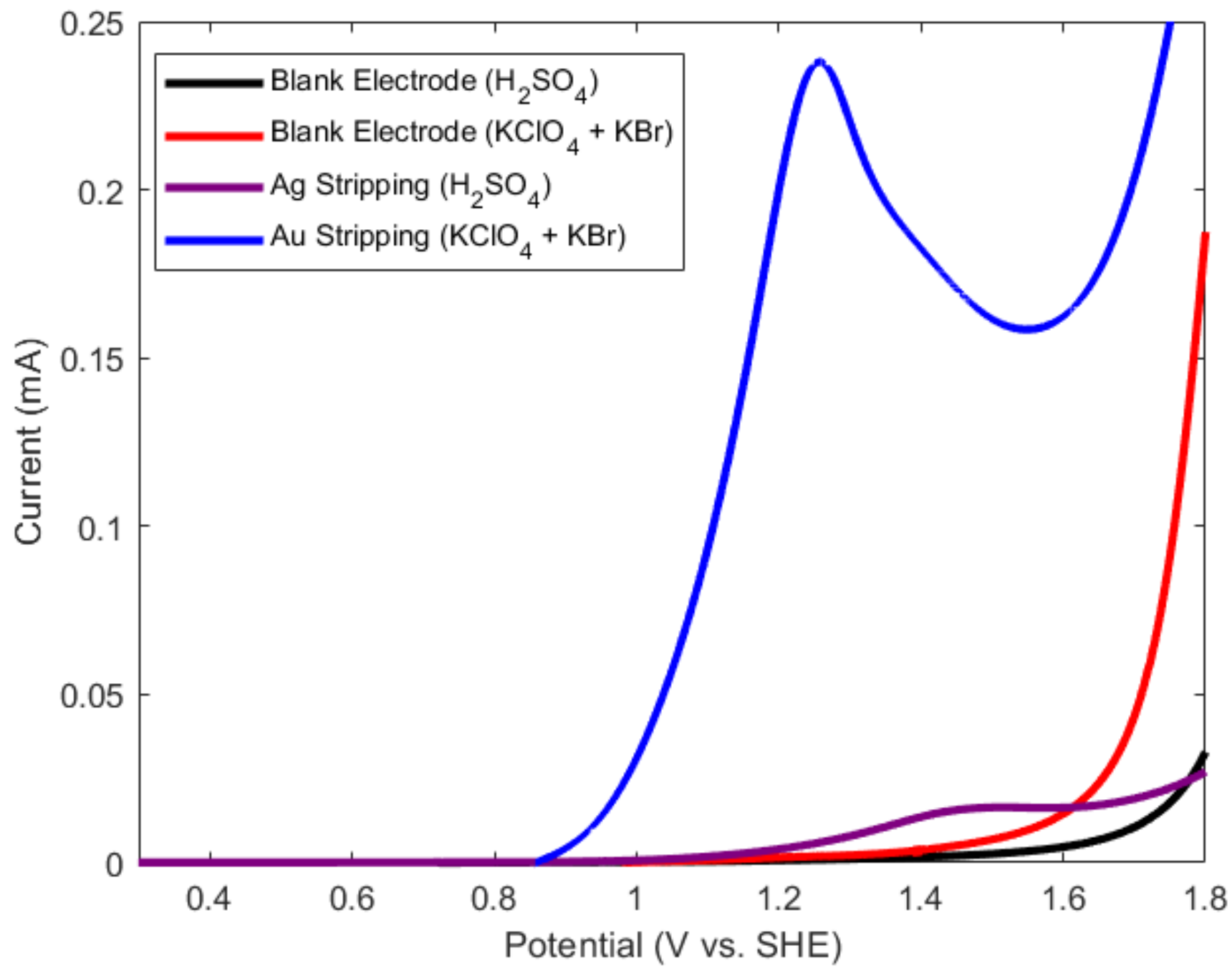

Figure S14. ASVs of electrodeposited (Ag)Au CC (500-fold diluted seed colloid), cleaned minimally with warm water, showing a positive shift in the position of the Ag stripping peak (purple), while the Au peak position (blue) is typical. The ASV for Ag was obtained in 100 mM H_2SO_4 electrolyte solution first and then the ASV for Au was obtained in a 10 mM KBr plus 100 mM $KClO_4$ electrolyte solution, both at a scan rate of 30 mV/s. The peak between 1.6 and 1.8 V vs. SHE is attributable to water splitting.

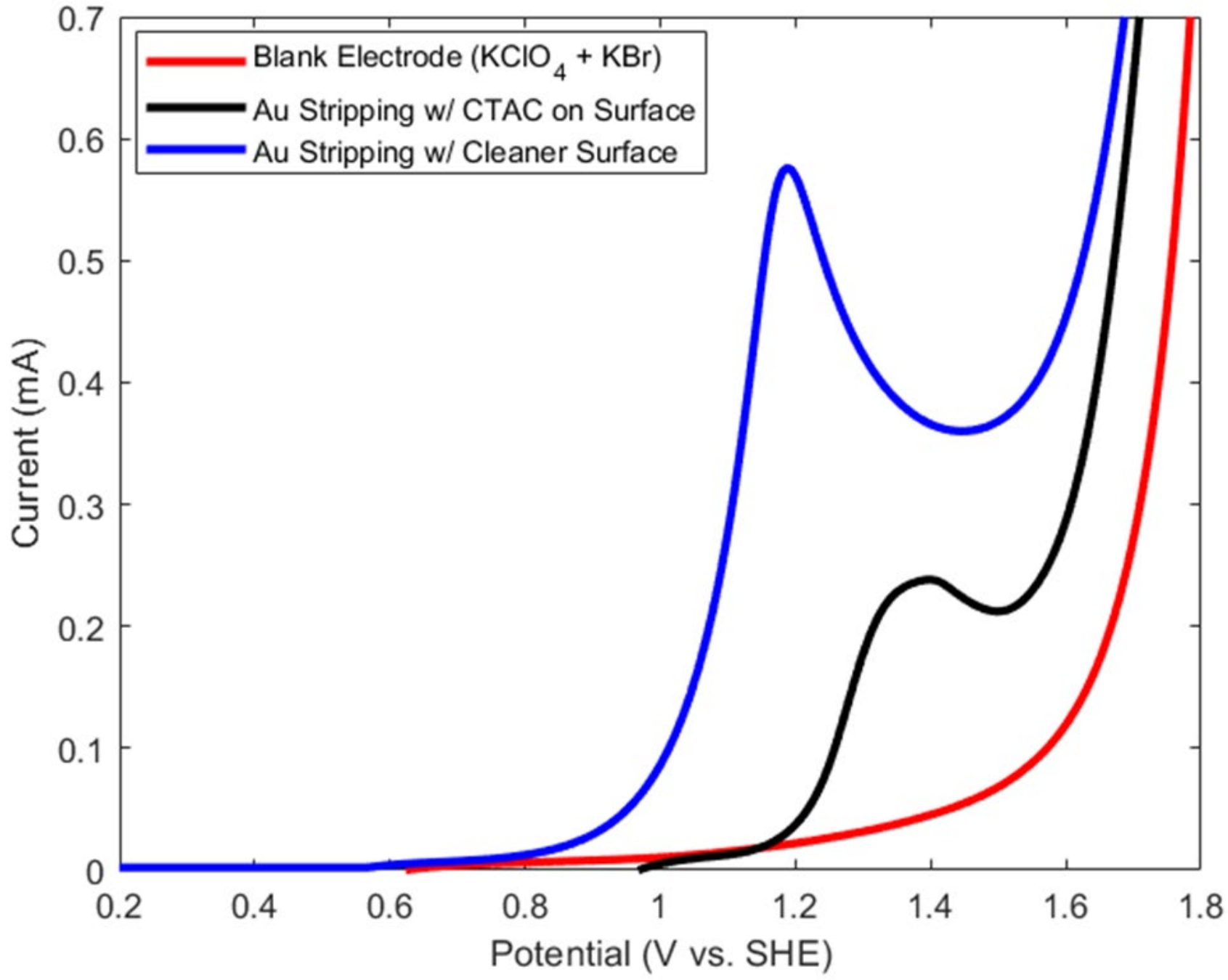

Figure S15. ASVs of electrodeposited monometallic Au nanoparticles (200-fold diluted seed colloid), cleaned minimally with warm water (black) or cleaned repeatedly with warm water and methanol (blue). When the electrode is cleaned more minimally, more CTAC is present on the particles' surfaces, causing a decrease in current magnitude and positive shift in the potential of the Au stripping peak. The ASV for Au was obtained in a 10 mM KBr plus 100 mM $KClO_4$ electrolyte solution, without any prior stripping steps, at a scan rate of 30 mV/s. The peak between 1.6 and 1.8 V vs. SHE is attributable to water splitting.

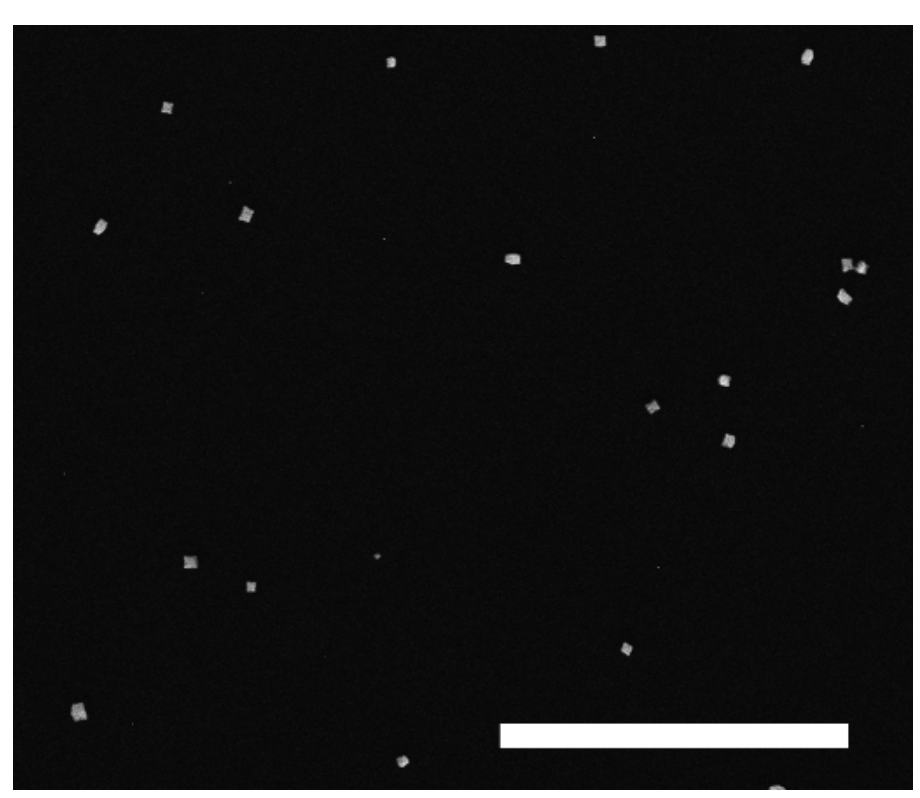

Figure S16. SEM image of electrodeposited (Ag)Au CC nanoparticles following washing four times with warm water and one time with warm methanol. This electrode was not subject to ASV experiments. The image confirms that the density of particles on the electrode is not significantly decreased by the washing steps taken before ASV. Scale bar: 10 µm.

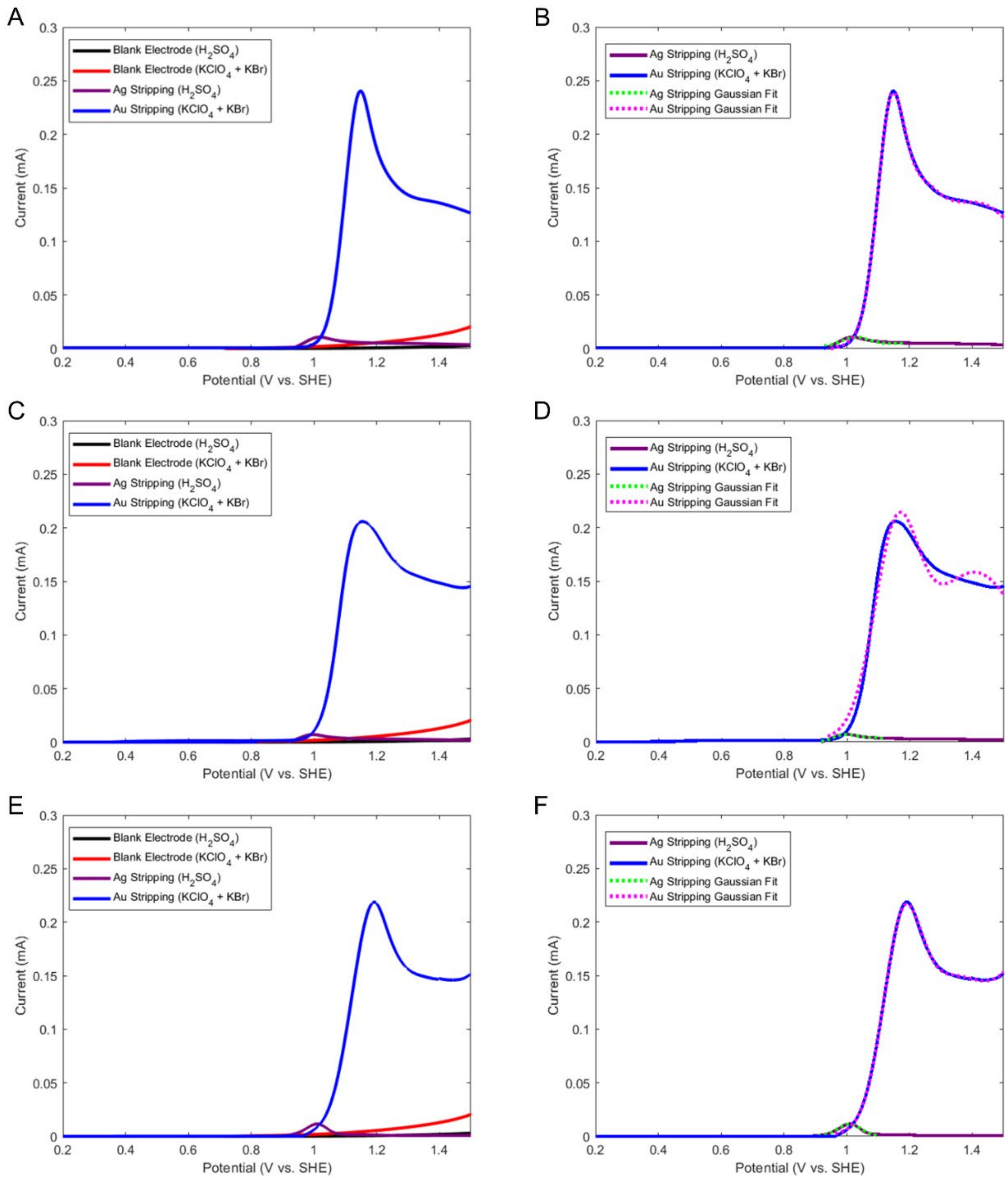

Figure S17. (A, C, E) Triplicate ASVs of electrodeposited (Ag)Au CC (500-fold diluted seed colloid), cleaned four times with warm water and one time with warm methanol. The Ag peak position (purple) is consistent with UPD of a submonolayer of Ag on Au. The Au peak position (blue) is typical of bulk Au deposition. The ASV for Ag was obtained in 100 mM H_2SO_4 electrolyte solution first and then the ASV for Au was obtained in a 10 mM KBr plus 100 mM $KClO_4$ electrolyte solution, both at a scan rate of 30 mV/s. The peak between 1.1 and 1.5 V vs. SHE in the ASV of a blank electrode in 10 mM KBr plus 100 mM $KClO_4$ electrolyte (red) is attributable to the onset of water splitting. (B, D, F) Gaussian fits of the Ag and Au ASVs used to integrate stripping peaks to obtain Ag:Au charge ratios. The Ag stripping peak was fit with a single Gaussian (dotted green) and the Au stripping peak was fit with a two-term Gaussian (dotted pink) to account for a shoulder peak and/or convolution with the water-splitting peak. See Tables S1 and S2 for further detail about Gaussian fits displayed in (B, D, F).

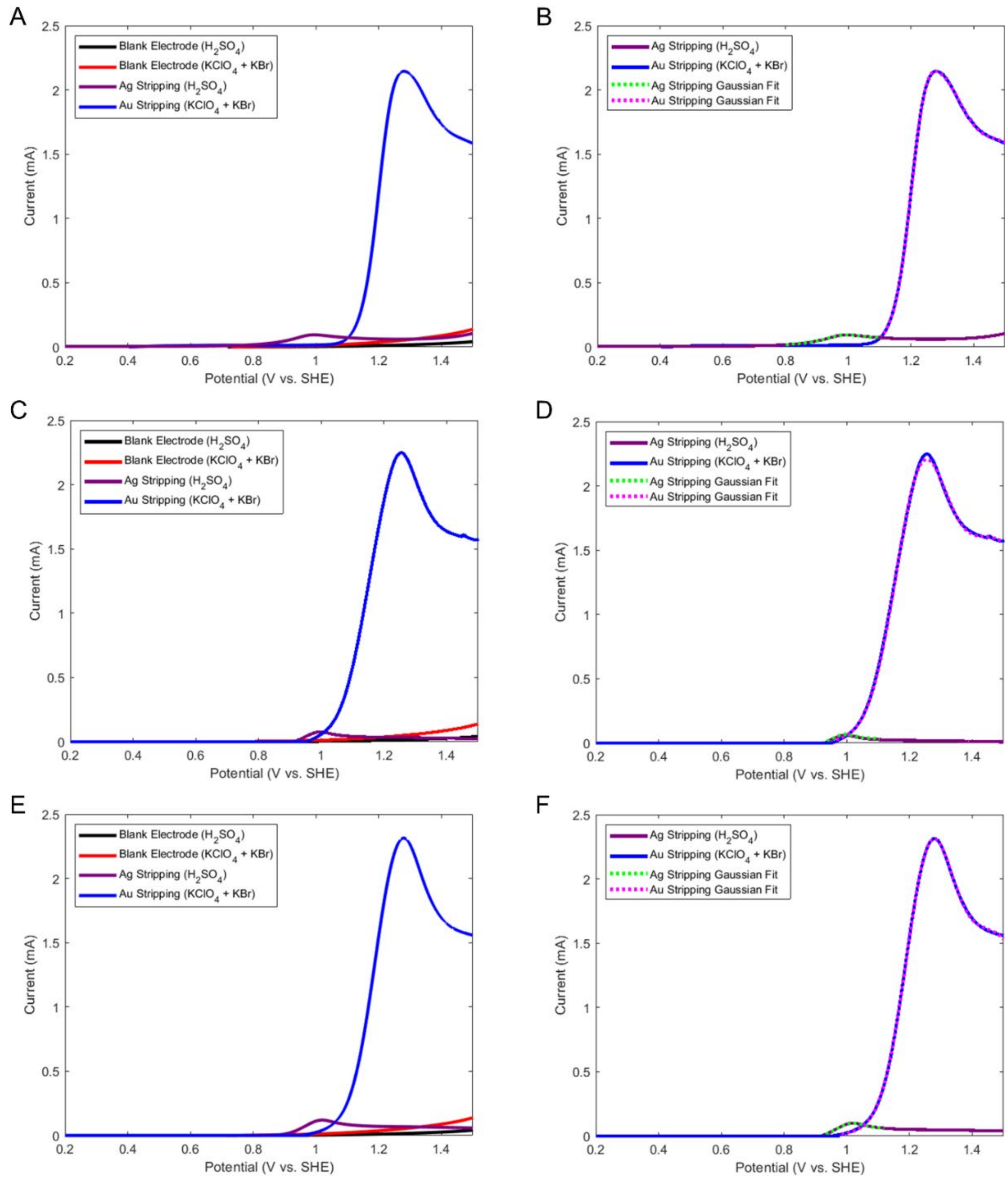

Figure S18. (A, C, E) Triplicate ASVs of colloidal (Ag)Au CC, washed five times and dropcast on a GCE in a 1:1 mixture of water and methanol. The Ag peak position (purple) is consistent with UPD of a submonolayer of Ag on Au. The Au peak position (blue) is typical of bulk Au deposition. The ASV for Ag was obtained in 100 mM H_2SO_4 electrolyte solution first and then the ASV for Au was obtained in a 10 mM KBr plus 100 mM $KClO_4$ electrolyte solution, both at a scan rate of 30 mV/s. The peak between 1.1 and 1.5 V vs. SHE in the ASV of a blank electrode in10 mM KBr plus 100 mM $KClO_4$ electrolyte (red) is attributable to the onset of water splitting. (B, D, F) Gaussian fits of the Ag and Au ASVs used to integrate stripping peaks to obtain Ag:Au charge ratios. The Ag stripping peak was fit with a single Gaussian (dotted green) and the Au stripping peak was fit with a two-term Gaussian (dotted pink) to account for a shoulder peak and/or convolution with the water-splitting peak. See Tables S1 and S2 for further detail about Gaussian fits displayed in (B, D, F).

Table S1. Gaussian Fits for Ag Stripping Peaks.

Sample	Fit for Current* (Single-Term Gaussian)	R^2
Electrodeposited (Ag)Au CC; Replicate 1; Ag Stripping	$0.0084 \cdot e^{-\frac{(x-1.0134)^2}{(2\cdot 0.0542)^2}}$	0.9897
Electrodeposited (Ag)Au CC; Replicate 2; Ag Stripping	$0.0104 \cdot e^{-\frac{(x-0.9889)^2}{(2\cdot 0.0468)^2}}$	0.9927
Electrodeposited (Ag)Au CC; Replicate 3; Ag Stripping	$0.0111 \cdot e^{-\frac{(x-1.0090)^2}{(2\cdot 0.0550)^2}}$	0.9302
Colloidal (Ag)Au CC; Replicate 1; Ag Stripping	$0.1029 \cdot e^{-\frac{(x-0.9831)^2}{(2\cdot 0.0529)^2}}$	0.9904
Colloidal (Ag)Au CC; Replicate 2; Ag Stripping	$0.1186 \cdot e^{-\frac{(x-1.0189)^2}{(2\cdot 0.0771)^2}}$	0.9980
Colloidal (Ag)Au CC; Replicate 3; Ag Stripping	$0.1051 \cdot e^{-\frac{(x-1.0317)^2}{(2\cdot 0.0690)^2}}$	0.9955

*Calculated using MATLAB Curve Fitter

Table S2. Gaussian Fits for Au Stripping Peaks.

Sample	Fit for Current* (Two-Term Gaussian)**	R^2
Electrodeposited (Ag)Au CC; Replicate 1; Au Stripping	$\left(0.1781 \cdot e^{-\frac{(x-1.1514)^2}{(2\cdot 0.0725)^2}}\right) + \left(0.1489 \cdot e^{-\frac{(x-1.3695)^2}{(2\cdot 0.2245)^2}}\right)$	0.9839
Electrodeposited (Ag)Au CC; Replicate 2; Au Stripping	$\left(0.1549 \cdot e^{-\frac{(x-1.1553)^2}{(2\cdot 0.0968)^2}}\right) + \left(0.1581 \cdot e^{-\frac{(x-1.4069)^2}{(2\cdot 0.2267)^2}}\right)$	0.9299
Electrodeposited (Ag)Au CC; Replicate 3; Au Stripping	$\left(0.1635 \cdot e^{-\frac{(x-1.1750)^2}{(2\cdot 0.0839)^2}}\right) + \left(0.1270 \cdot e^{-\frac{(x-1.3782)^2}{(2\cdot 0.2817)^2}}\right)$	0.9998
Colloidal (Ag)Au CC; Replicate 1; Au Stripping	$\left(1.5741 \cdot e^{-\frac{(x-1.268)^2}{(2\cdot 0.1032)^2}}\right) + \left(1.6438 \cdot e^{-\frac{(x-1.5515)^2}{(2\cdot 0.2733)^2}}\right)$	0.9733
Colloidal (Ag)Au CC; Replicate 2; Au Stripping	$\left(1.5979 \cdot e^{-\frac{(x-1.2215)^2}{(2\cdot 0.1039)^2}}\right) + \left(1.5629 \cdot e^{-\frac{(x-1.4655)^2}{(2\cdot 0.2225)^2}}\right)$	0.9999
Colloidal (Ag)Au CC; Replicate 3; Au Stripping	$\left(1.5614 \cdot e^{-\frac{(x-1.2512)^2}{(2\cdot 0.1146)^2}}\right) + \left(1.5737 \cdot e^{-\frac{(x-1.4718)^2}{(2\cdot 0.2440)^2}}\right)$	0.9996

*Calculated using MATLAB Curve Fitter
**The first Gaussian term corresponds to the bulk Au stripping peak, while the second Gaussian term corresponds to the shoulder of a water splitting peak

Table S3. Ag and Au Anodic Stripping Voltammetry (ASV) Peak Potentials and Calculated Values.

ASV Sample	Ag Stripping E_p (V vs. SHE)	Au Stripping E_p (V vs. SHE)	Calculated Ag Stripping Charge (µC)	Calculated Au Stripping Charge (mC)	Calculated Ag:Au Ratio*
Electrodeposited (Ag)Au CC; Replicate 1	1.02	1.16	14.06	3.763	0.0112
Electrodeposited (Ag)Au CC; Replicate 2	0.99	1.15	10.29	2.997	0.0103
Electrodeposited (Ag)Au CC; Replicate 3	1.01	1.19	13.15	3.056	0.0129
Colloidal (Ag)Au CC; Replicate 1	0.98	1.25	69.07	19.98	0.0104
Colloidal (Ag)Au CC; Replicate 2	1.01	1.22	80.63	20.92	0.0116
Colloidal (Ag)Au CC; Replicate 3	1.04	1.25	98.90	22.35	0.0133

*Compare to a ~0.012 Ag:Au ratio obtained by ICP-OES for colloidal (Ag)Au CC of similar size[11]

Table S4. Shape Distribution of (Ag)Au Nanoparticles Electrodeposited with 9.2 µM Ag^+ and 20-Fold Increase in Au Seed Concentration.

Shape	Count (n = 1382)	%
concave cube	378	27.4
concave right bipyramid (single twinned)	33	2.4
decahedron (5-fold twinned)	207	15.0
rhombic dodecahedron	4	0.3
octahedron	7	0.5
triangular plate	6	0.4
small pseudospherical	426	30.8
not shaped or undefined facets	321	23.2

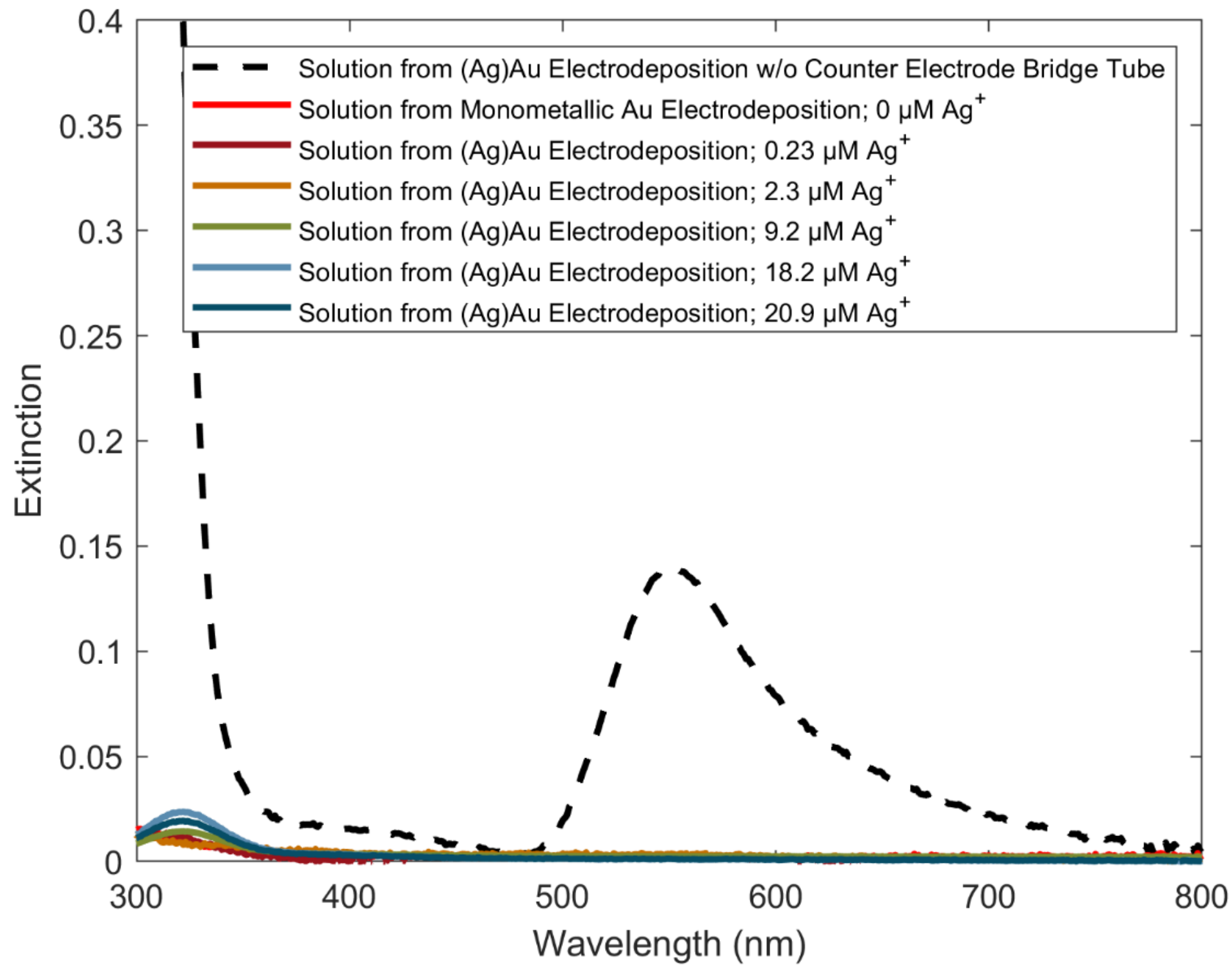

Figure S19. UV-vis spectra of growth solutions following electrodeposition of monometallic Au and (Ag)Au nanoparticles with varied [Ag^+] and -0.815 µA/cm^2 applied for 60 minutes, showing a lack of solution phase nanoparticle growth. A representative growth solution following an electrodeposition synthesis of (Ag)Au nanoparticles (-0.815 µA/cm^2 applied for 60 minutes) with a Pt wire counter electrode inserted directly into solution, in which solution-phase growth of (Ag)Au nanoparticles occurs due to chemical reduction, is provided for comparison.

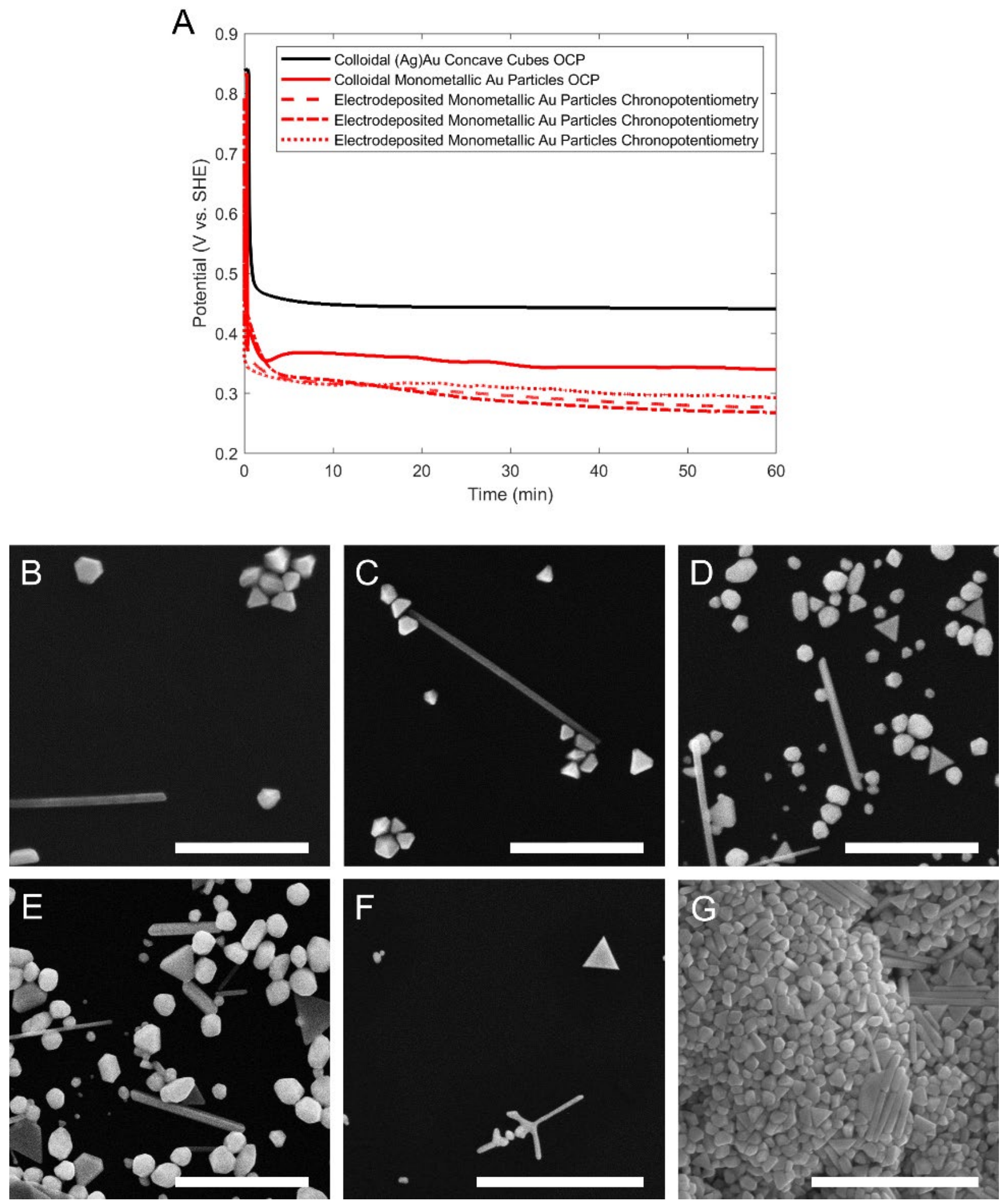

Figure S20. (A) Comparison between OCP measurements of colloidal (Ag)Au CC (black) and colloidal monometallic Au (solid red) nanoparticle growth and chronopotentiometry measurements (dotted red) of galvanostatic electrodeposition of monometallic Au nanoparticles in 125 mM CTAC with -0.815 $\mu A/cm^2$ applied current for 60 minutes. (B-F) Representative SEM images of electrodeposited monometallic Au nanoparticles, showing a range of shapes including rods, octahedra, triangular plates, and truncated bitetrahedra. (G) SEM image of colloidal monometallic Au nanoparticles for comparison. Scale bars: 1 µm. Solid black and red traces in (A) adapted with permission from ref. 12. Available under a Creative Commons CC-BY 4.0 license. Copyright 2026 Halford, Sublett, and Personick.

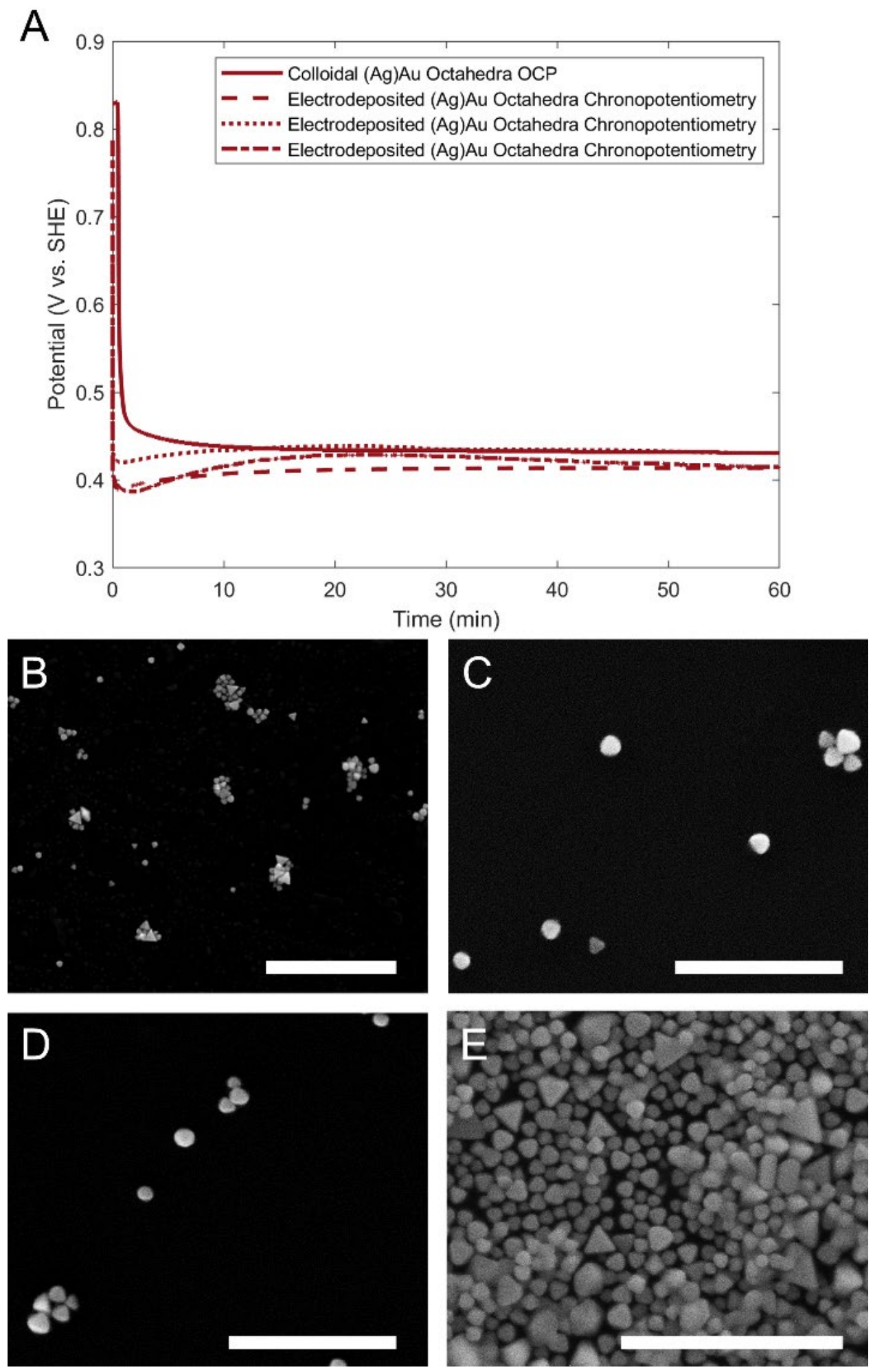

Figure S21. (A) Comparison between OCP measurement of colloidal (Ag)Au octahedra growth and chronopotentiometry measurements of galvanostatic electrodeposition of (Ag)Au octahedra in 125 mM CTAC with 0.23 µM Ag^+ and -0.815 µA/cm^2 applied current for 60 minutes. (B-D) Representative SEM images of electrodeposited (Ag)Au octahedral nanoparticles, showing (B) representative deposition density and (C-D) particle morphologies. (E) SEM images of colloidal (Ag)Au octahedra for comparison. Scale bars: 5 µm (B) and 1 µm (C-E). Solid red trace in (A) adapted with permission from ref. 12. Available under a Creative Commons CC-BY 4.0 license. Copyright 2026 Halford, Sublett, and Personick.

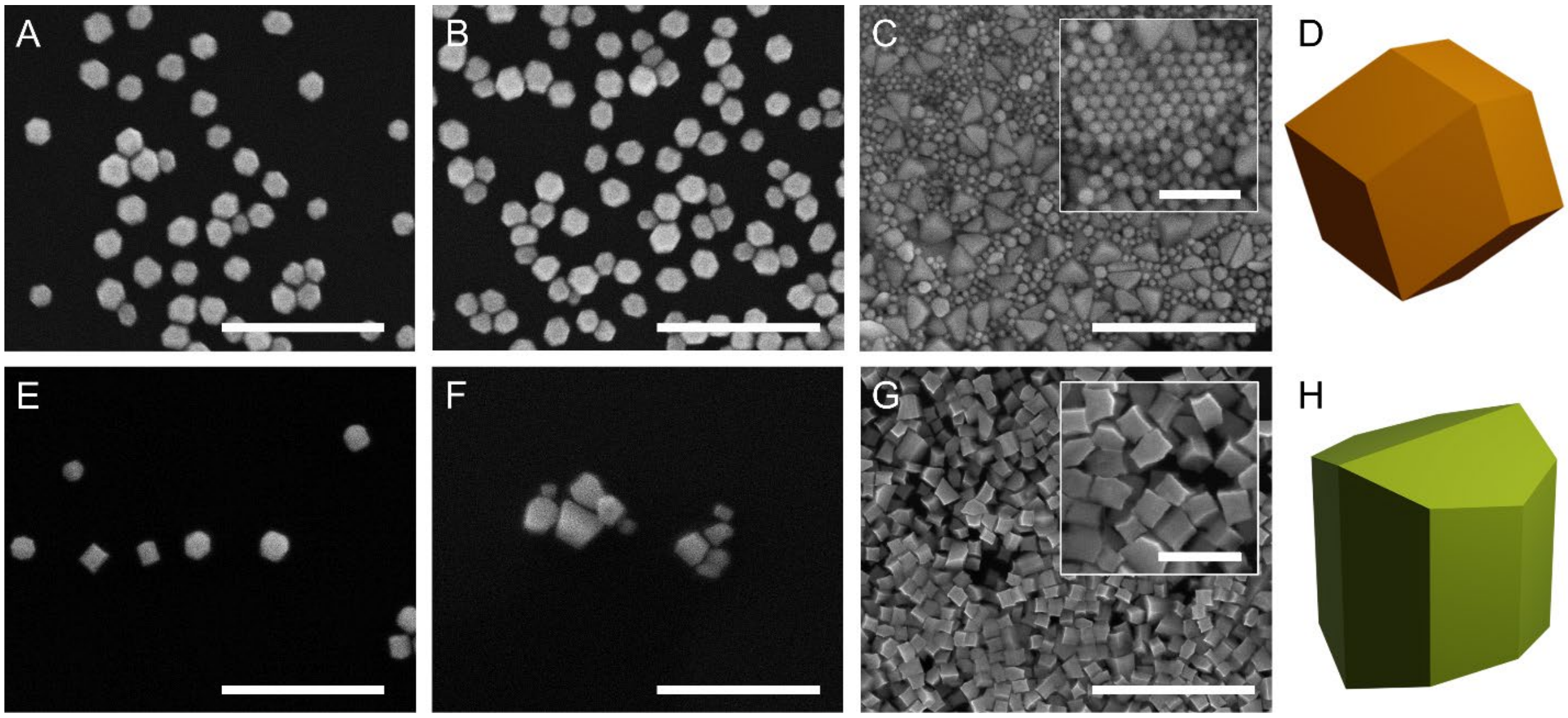

Figure S22. (A-B) Additional representative SEM images of electrodeposited (Ag)Au RD nanoparticles deposited onto Au seeds (200-fold diluted seed colloid; standard surface coverage). Note that the area of the electrode shown in image (B) has an atypically high density of electrodeposited nanoparticles. (C) SEM images of colloidal (Ag)Au RD and {110} bipyramids, for comparison. (D) Model of an RD. (E-F) Additional representative SEM images of electrodeposited (Ag)Au TDP nanoparticles deposited onto Au seeds (200-fold diluted seed colloid; standard surface coverage). (G) SEM images of colloidal (Ag)Au TDP for comparison. (H) Model of a TDP. Scale bars: 500 nm (A-C, E-G) and 125 nm (C and G insets).

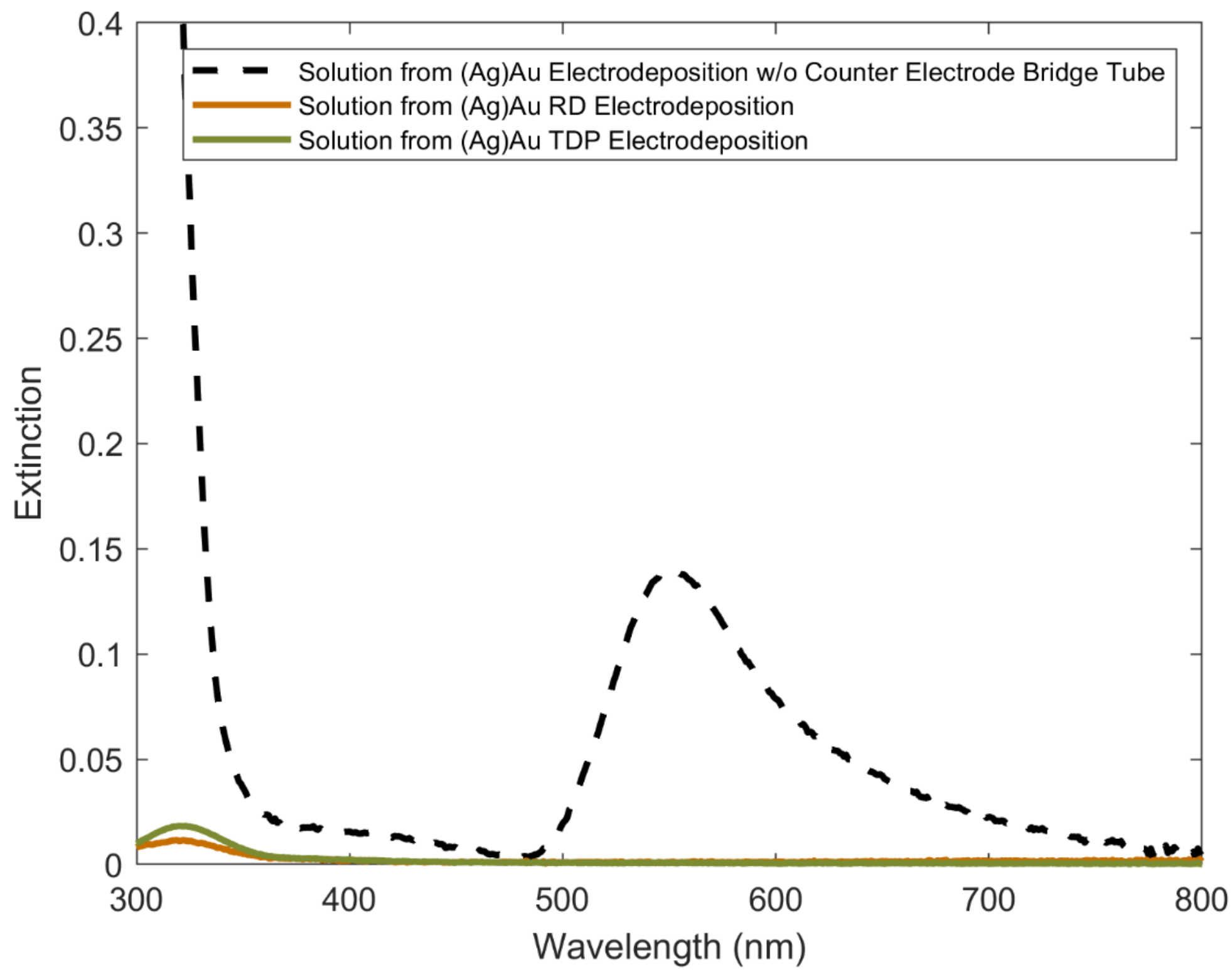

Figure S23. UV-vis spectra of growth solutions following electrodeposition of (Ag)Au RD and TDP nanoparticles deposited onto Au seeds (200-fold diluted seed colloid; standard surface coverage; -0.713 μA/cm^2 applied for 60 minutes for RD; -0.739 μA/cm^2 applied for 60 minutes for TDP), showing a lack of solution phase nanoparticle growth. A representative growth solution following an electrodeposition synthesis of (Ag)Au nanoparticles (-0.815 μA/cm^2 applied for 60 minutes) with a Pt wire counter electrode inserted directly into solution, in which solution-phase growth of (Ag)Au nanoparticles occurs due to chemical reduction, is provided for comparison.

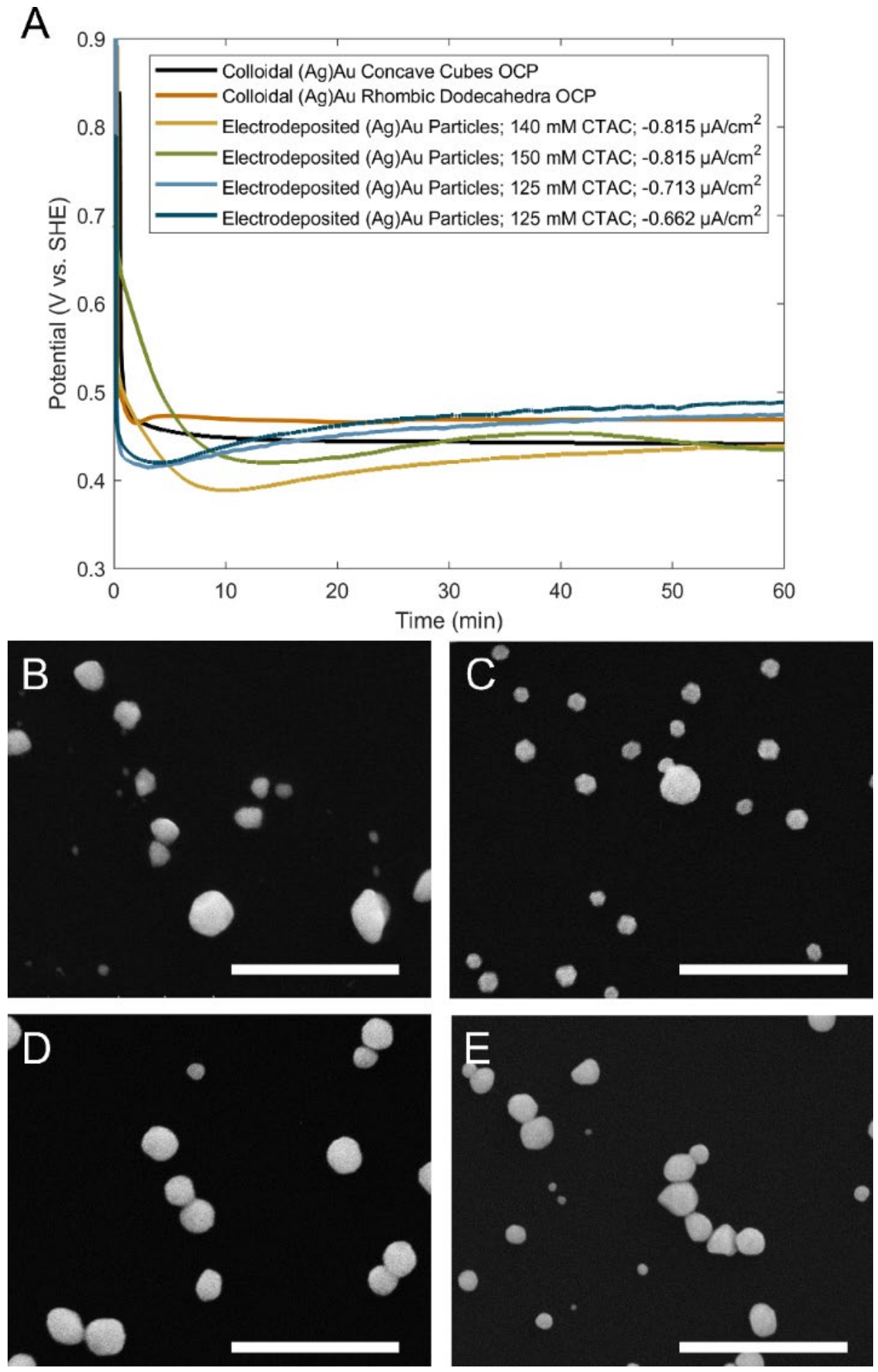

Figure S24. (A) Comparison between OCP measurements of colloidal (Ag)Au CC (black) and colloidal (Ag)Au rhombic dodecahedra (orange) growth and chronopotentiometry measurements of galvanostatic electrodeposition of (Ag)Au nanoparticles with 2.3 µM Ag^+, 125-150 mM CTAC, and -0.815 to -0.662 µA/cm^2 applied current for 60 minutes. (B-E) SEM images of (Ag)Au nanoparticles electrodeposited with (B) 140 mM CTAC and -0.815 µA/cm^2 applied current (yellow trace), (C) 150 mM CTAC and -0.815 µA/cm^2 applied current (light green trace), (D) 125 mM CTAC and -0.713 µA/cm^2 applied current (light blue trace), and (E) 125 mM CTAC and -0.662 µA/cm^2 applied current (teal trace). Scale bars: 1 µm. Black and orange traces in (A) adapted with permission from ref. 12. Available under a Creative Commons CC-BY 4.0 license. Copyright 2026 Halford, Sublett, and Personick.

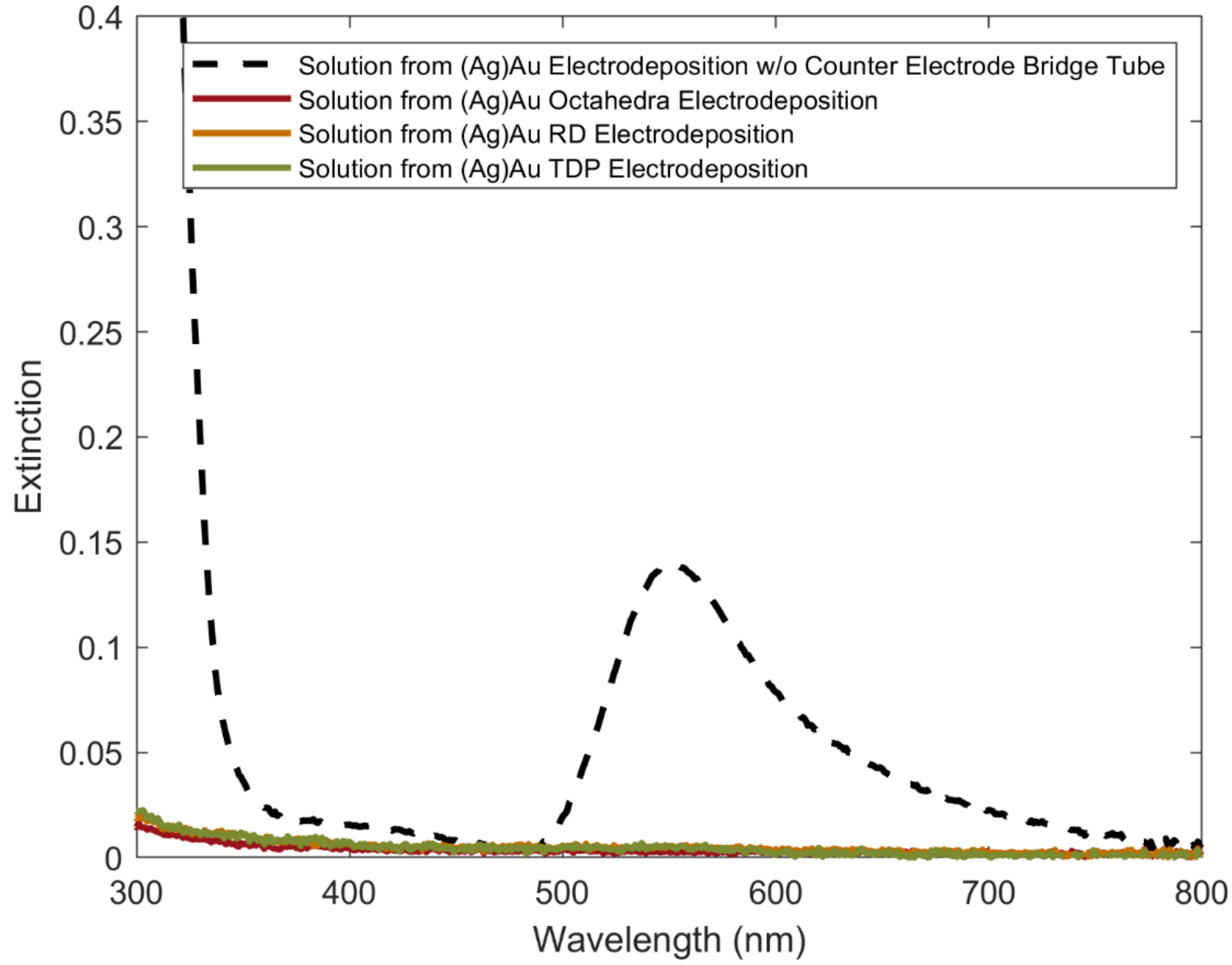

Figure S25. UV-vis spectra of growth solutions following electrodeposition of (Ag)Au octahedra, RD, and TDP nanoparticles using 500-fold diluted seed colloid (-0.739 μA/cm^2 applied for 60 minutes for octahedra and TDP; -0.713 μA/cm^2 applied for 60 minutes for RD), showing a lack of solution phase nanoparticle growth. A representative growth solution following an electrodeposition synthesis of (Ag)Au nanoparticles (-0.815 μA/cm^2 applied for 60 minutes) with a Pt wire counter electrode inserted directly into solution, in which solution-phase growth of (Ag)Au nanoparticles occurs due to chemical reduction, is provided for comparison.

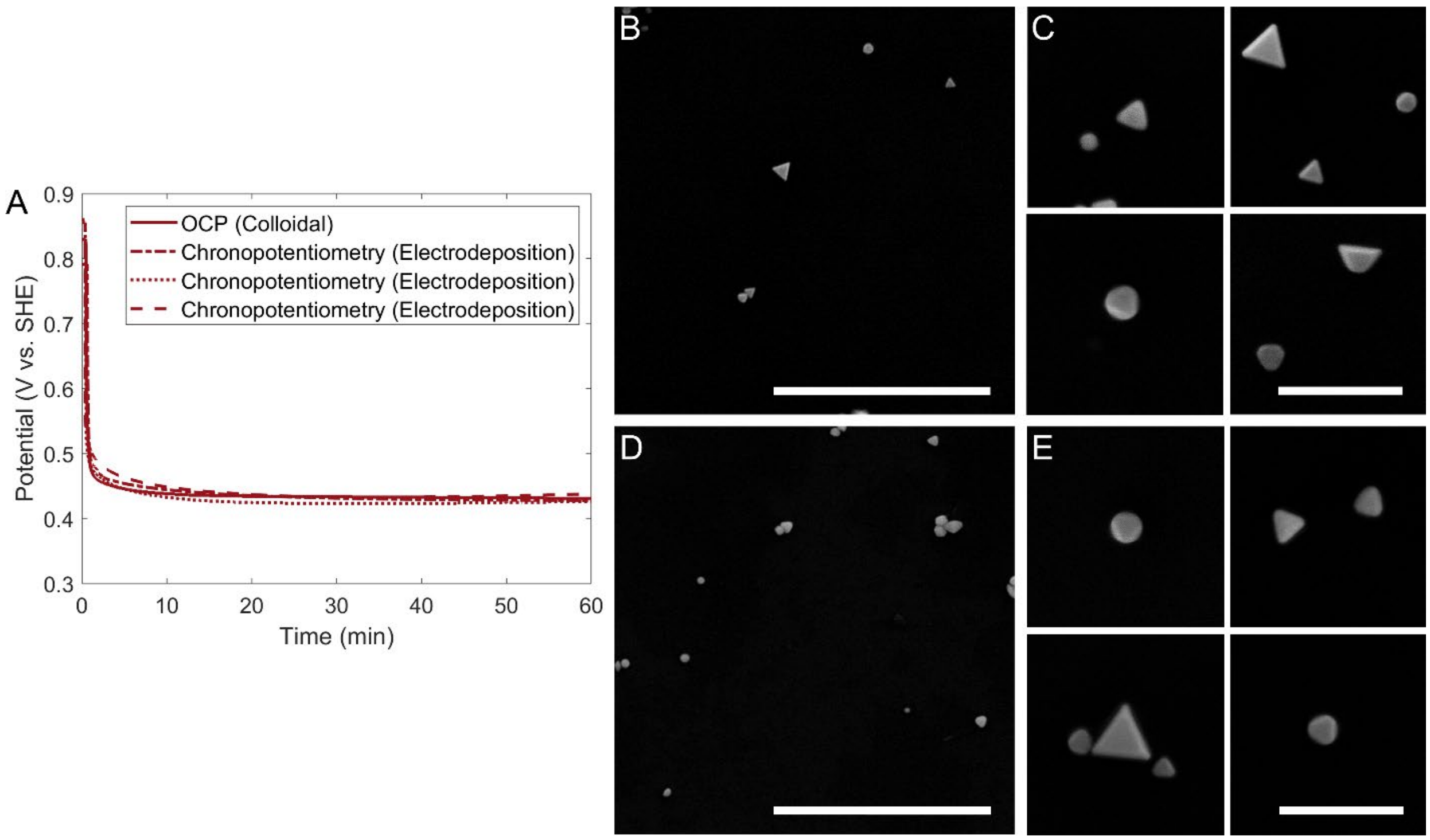

Figure S26. (A) Comparison between OCP measurements of colloidal octahedra growth and triplicate chronopotentiometry measurements of galvanostatic electrodeposition of octahedra. Octahedra were electrodeposited in 125 mM CTAC with 0.33 µM Ag^+ and -0.739 µA/cm^2 applied current for 60 minutes, onto Au seeds (500-fold diluted seed colloid). (B-E) Representative SEM images of electrodeposited octahedral nanoparticles from triplicate electrodeposition experiments, showing (B, D) deposition density and (C, E) high-magnification images of individual particles. See Figure 4A-C for SEM images from the first of the triplicate electrodeposition experiments. Scale bars: 5 µm (B, D) and 500 nm (C, E).

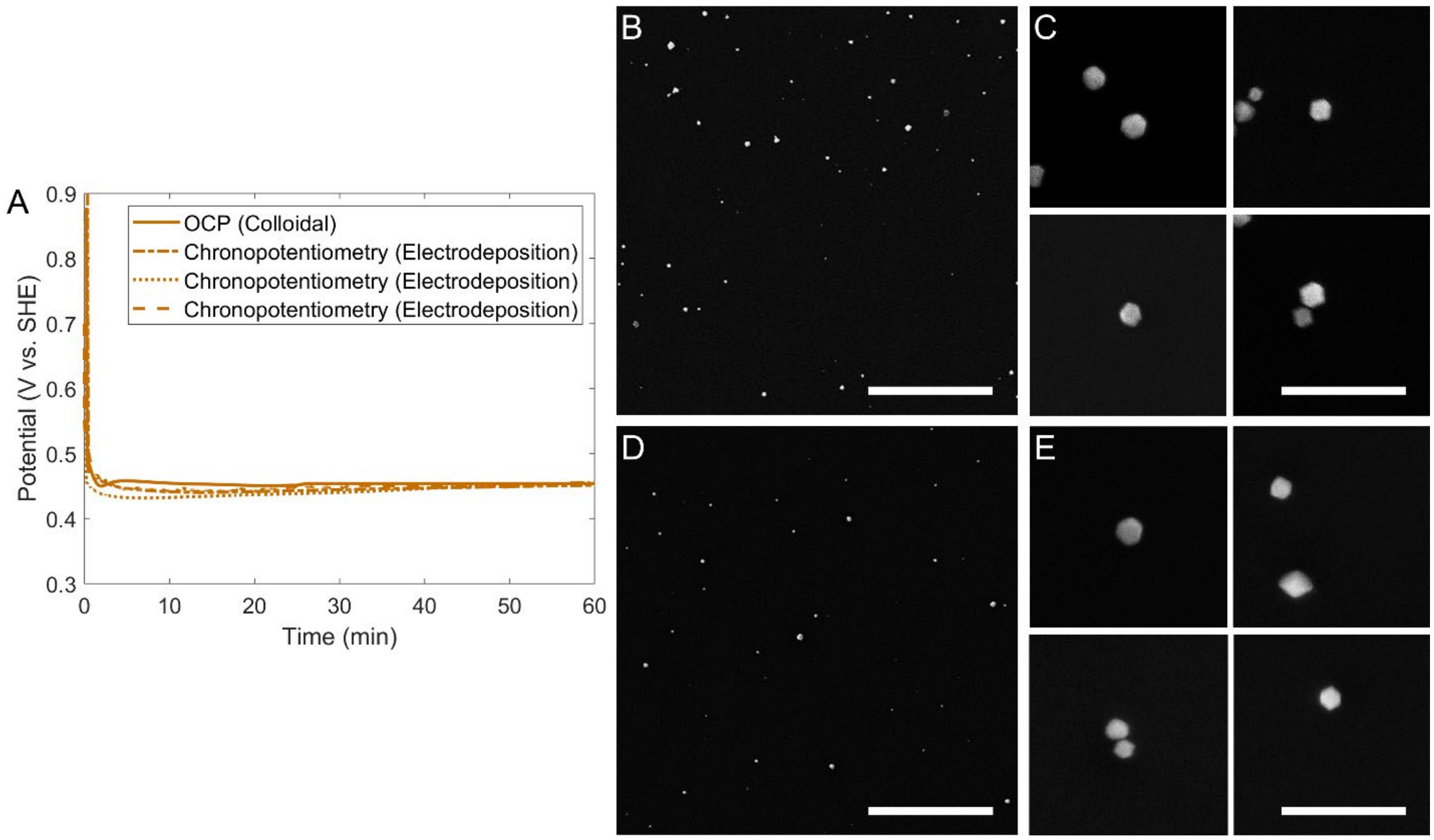

Figure S27. (A) Comparison between OCP measurements of colloidal RD growth and triplicate chronopotentiometry measurements of galvanostatic electrodeposition of RD. RD were electrodeposited in 140 mM CTAC with 3.3 µM Ag^{+} and -0.713 µA/cm^2 applied current for 60 minutes, onto Au seeds (500-fold diluted seed colloid). (B-E) Representative SEM images of electrodeposited RD nanoparticles from triplicate electrodeposition experiments, showing (B, D) deposition density and (C, E) high-magnification images of individual RD particles. See Figure 4D-F for SEM images from the first of the triplicate electrodeposition experiments. Scale bars: 5 µm (B, D) and 500 nm (C, E).

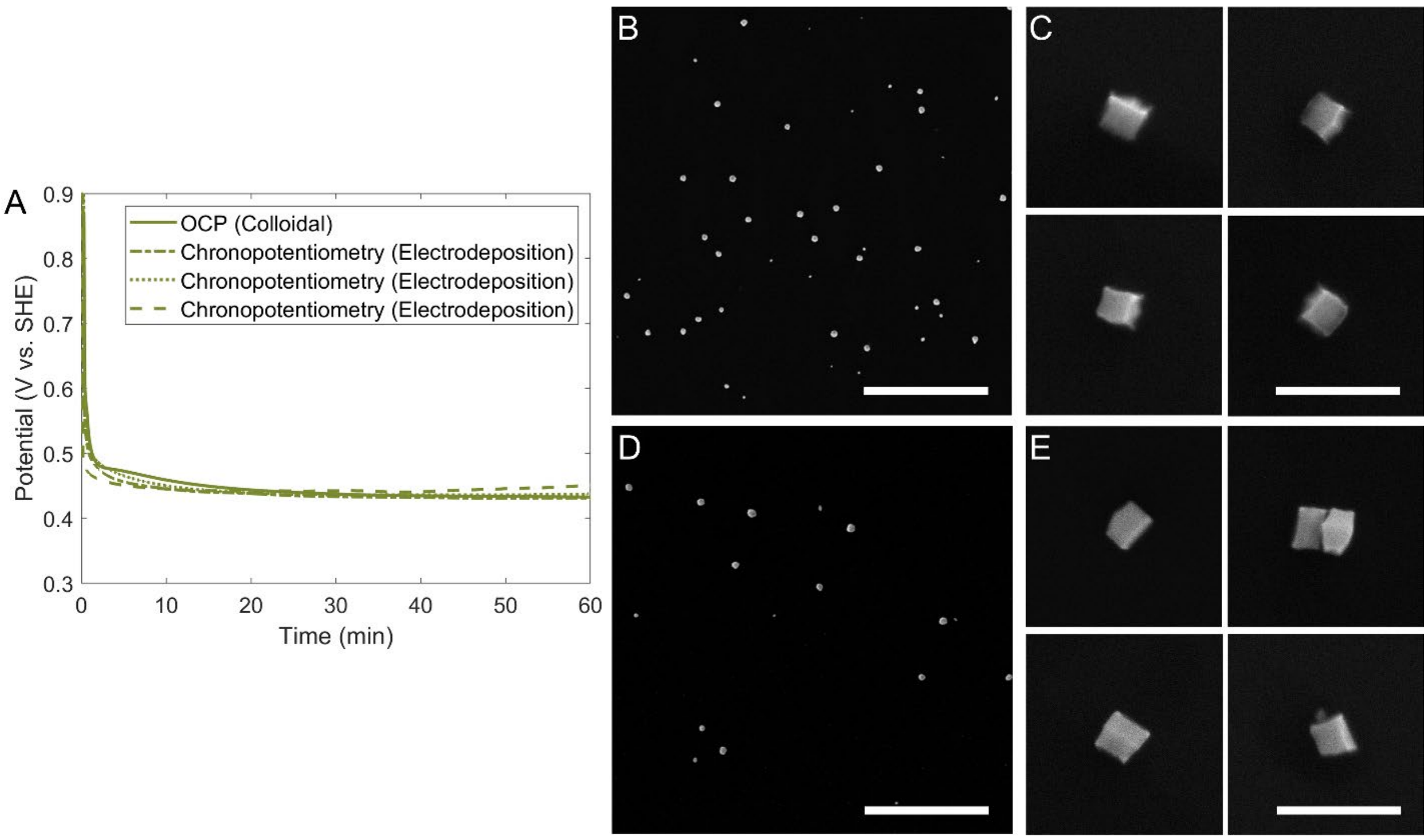

Figure S28. (A) Comparison between OCP measurements of colloidal TDP growth and triplicate chronopotentiometry measurements of galvanostatic electrodeposition of TDP. TDP were electrodeposited in 130 mM CTAC with 15.5 μM Ag^+ and -0.739 μA/cm^2 applied current for 60 minutes, onto Au seeds (500-fold diluted seed colloid). (B-E) Representative SEM images of electrodeposited TDP nanoparticles from triplicate electrodeposition experiments, showing (B, D) deposition density and (C, E) high-magnification images of individual TDP particles. See Figure 4G-I for SEM images from the first of the triplicate electrodeposition experiments. Scale bars: 5 μm (B, D) and 500 nm (C, E).

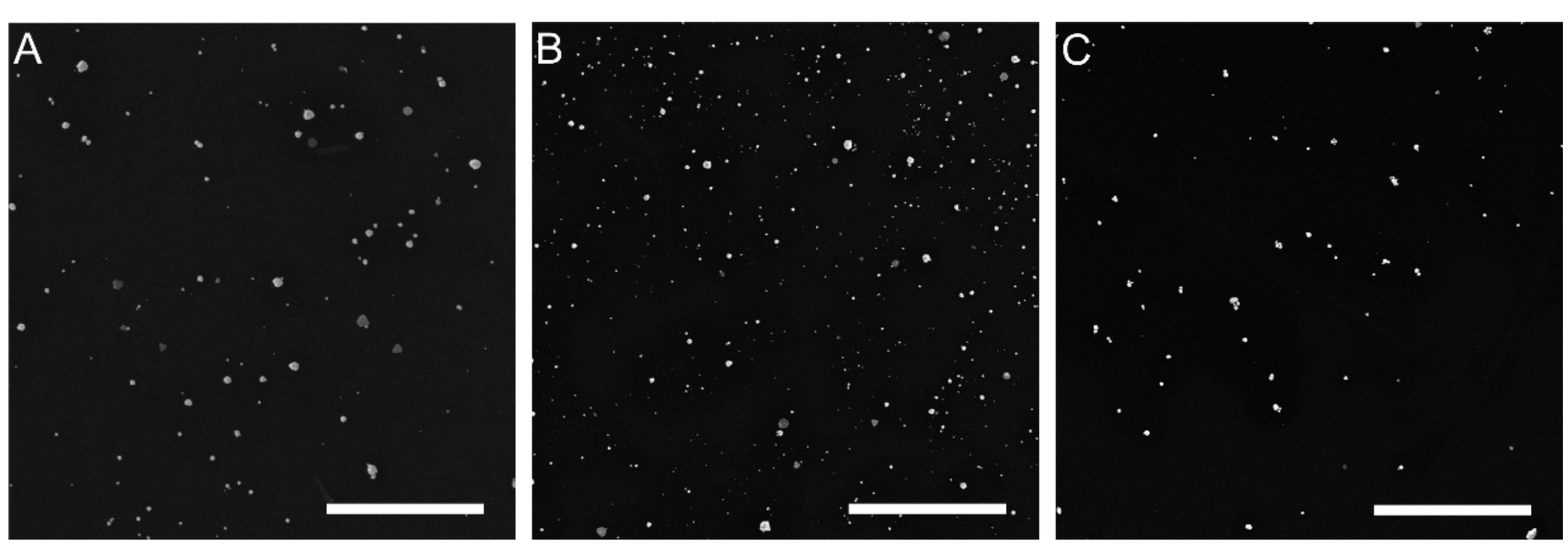

Figure S29. (A-C) Representative low-magnification SEM images used to determine particle surface density of electrodeposited (Ag)Au nanoparticles for different shapes deposited with 500-fold dilution of the seed colloid. (A) (Ag)Au octahedra; 3±1 particles per μm^2, (B) RD; 5±2 particles per μm^2, and (C) TDP; 4±2 particles per μm^2. These particle surface densities are comparable to electrodeposited (Ag)Au CC at the same seeding conditions (4±2 particles per μm^2). Scale bars: 10 μm.

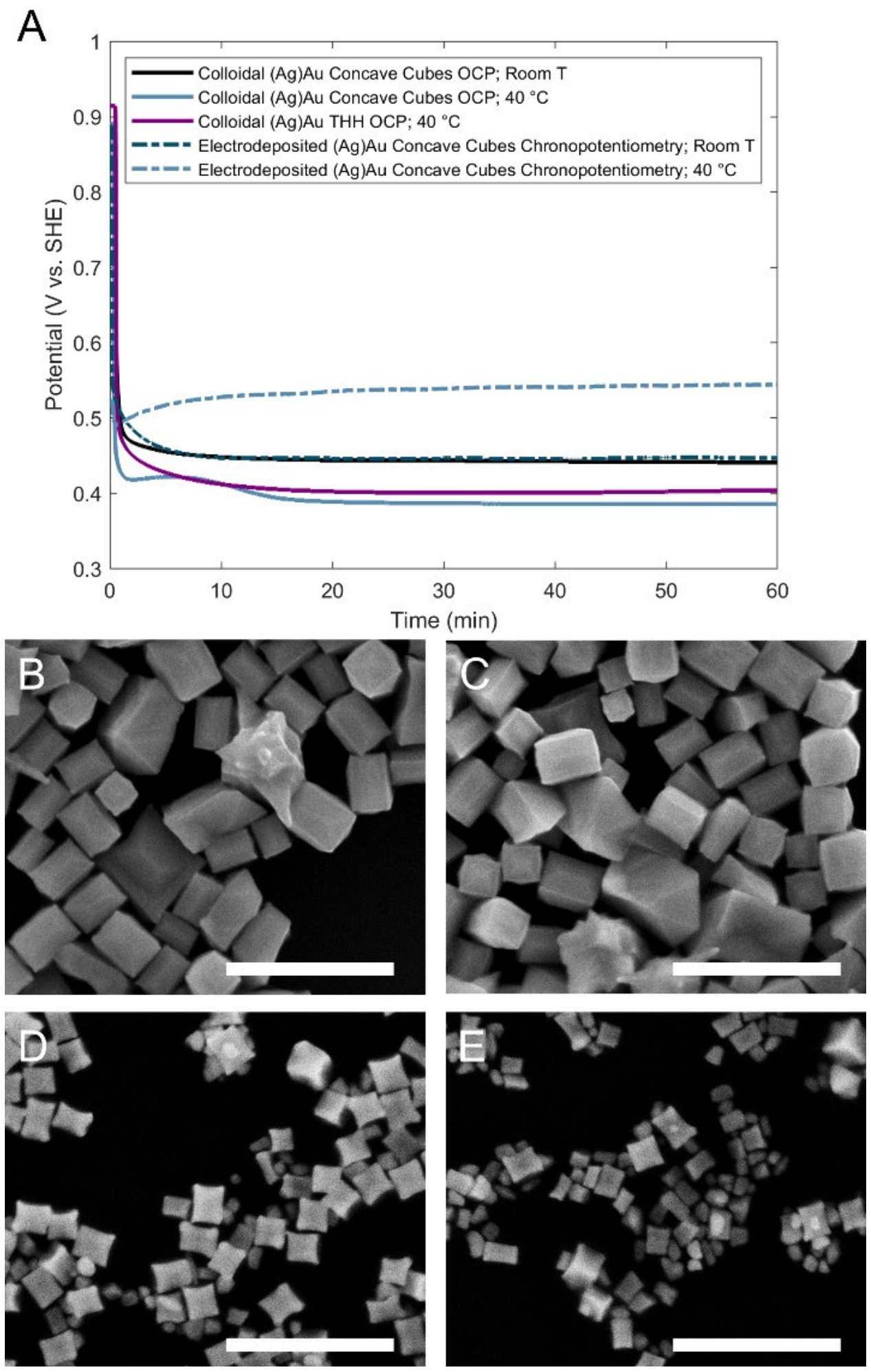

Figure S30. (A) Comparison between OCP measurement of colloidal (Ag)Au CC growth at room temperature (solid black), in CTAC at 40 °C (concave cubes; solid light blue), and in CTAB at 40 °C (elongated THH; solid purple) and chronopotentiometry measurements of galvanostatic electrodeposition of (Ag)Au CC in 125 mM CTAC with 20.9 µM Ag^{+} and -0.815 µA/cm^2 applied current for 60 minutes at room temperature (dotted dark teal) and at 40 °C (dotted light blue). (B-C) Representative SEM images of colloidal (Ag)Au elongated THH and side products from the reaction in CTAB at 40 °C. (D-E) Representative SEM images of (Ag)Au CC nanoparticles electrodeposited at 40 °C. Scale bars: 500 nm (B-C) and 1 µm (D-E). Black trace in (A) adapted with permission from ref. 12. Available under a Creative Commons CC-BY 4.0 license. Copyright 2026 Halford, Sublett, and Personick.

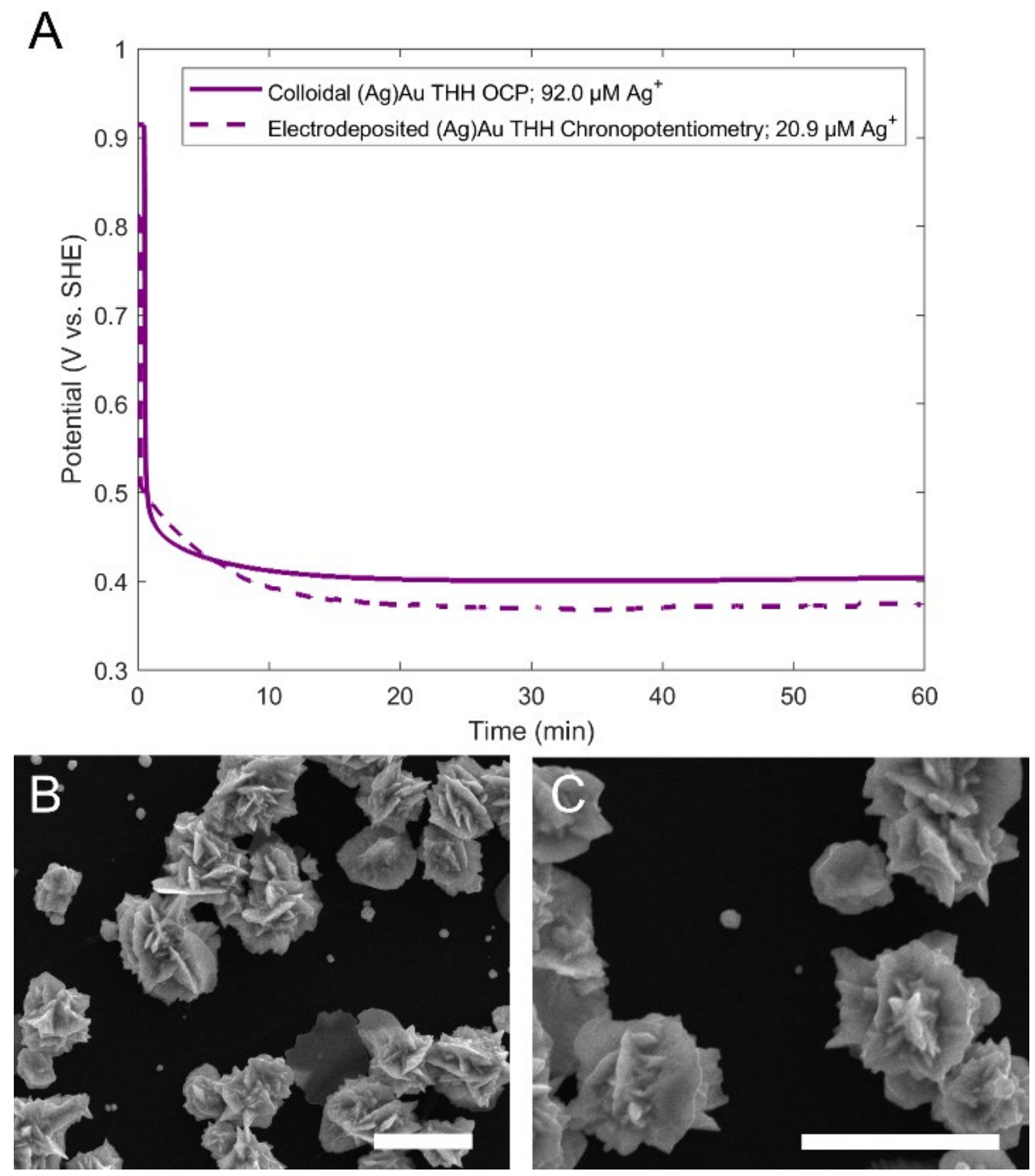

Figure S31. (A) Comparison between OCP measurement of (Ag)Au THH growth in 100 mM CTAB at 40 °C (solid purple) and chronopotentiometry measurement of galvanostatic electrodeposition of (Ag)Au nanoparticles in 125 mM CTAB at 40 °C with 20.9 μM Ag^+ and -0.815 μA/cm^2 applied current for 60 minutes, onto 500-fold diluted CTAB-capped Au seed colloid (dashed purple). (B-C) Representative SEM images of electrodeposited (Ag)Au nanoparticles. Scale bars: 1 μm.

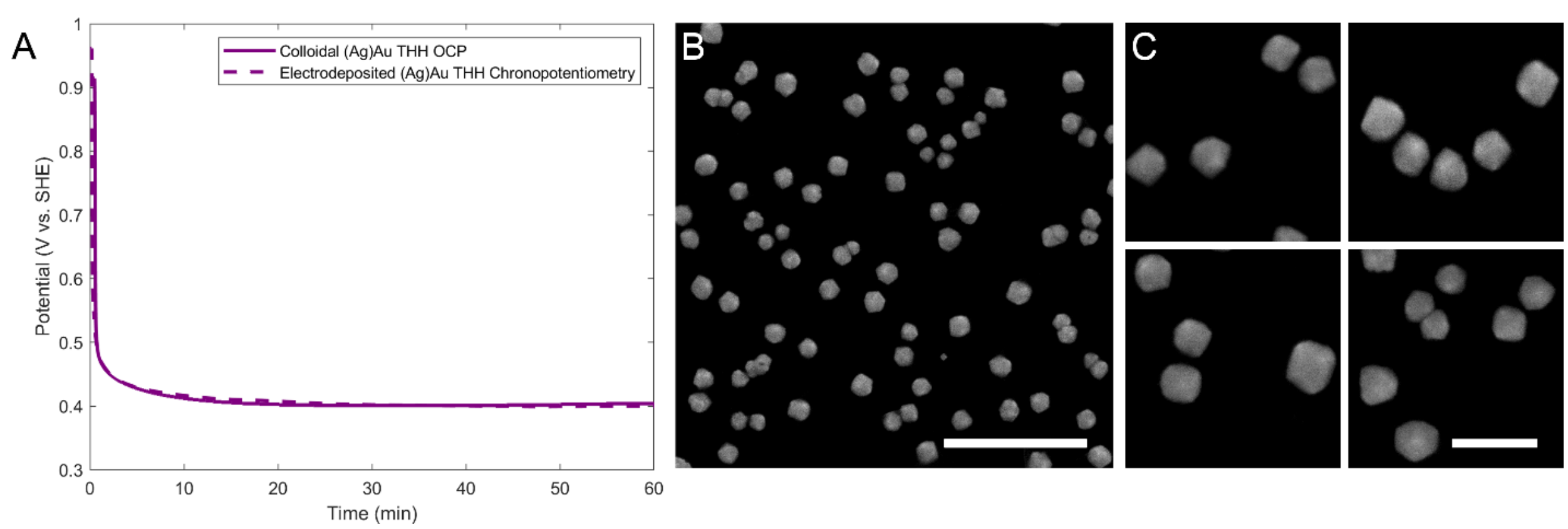

Figure S32. (A) Comparison between OCP measurements of colloidal THH growth and chronopotentiometry measurements of galvanostatic electrodeposition of THH in 125 mM CTAB with 1.3 μM Ag^+ and -0.866 μA/cm^2 applied current for 60 minutes, onto 500-fold diluted CTAB-capped Au seed colloid. (B-C) Representative SEM images of electrodeposited THH, showing (B) deposition density and (C) particle morphology. Scale bars: 3 μm (B) and 500 nm (C).

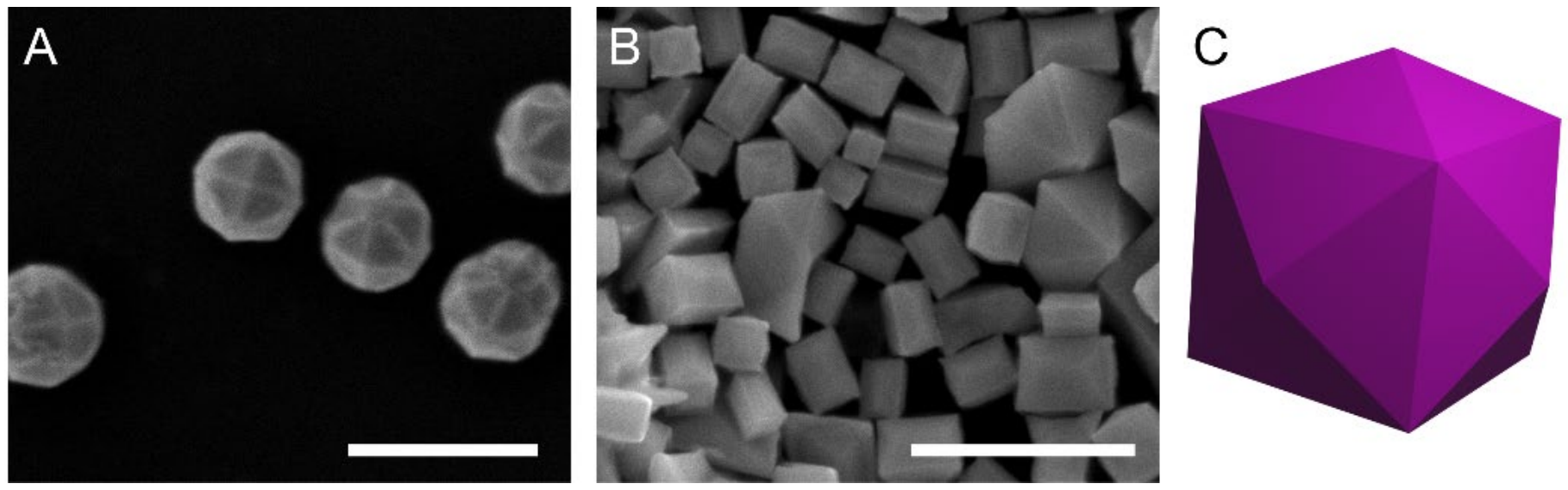

Figure S33. (A) Additional representative SEM image from the first replicate electrodeposition of (Ag)Au THH nanoparticles synthesized with 1000-fold diluted CTAB-capped Au seeds. See Figure 5. (B) SEM images of colloidal (Ag)Au elongated THH, for comparison. (C) Model of a THH. Scale bars: 500 nm.

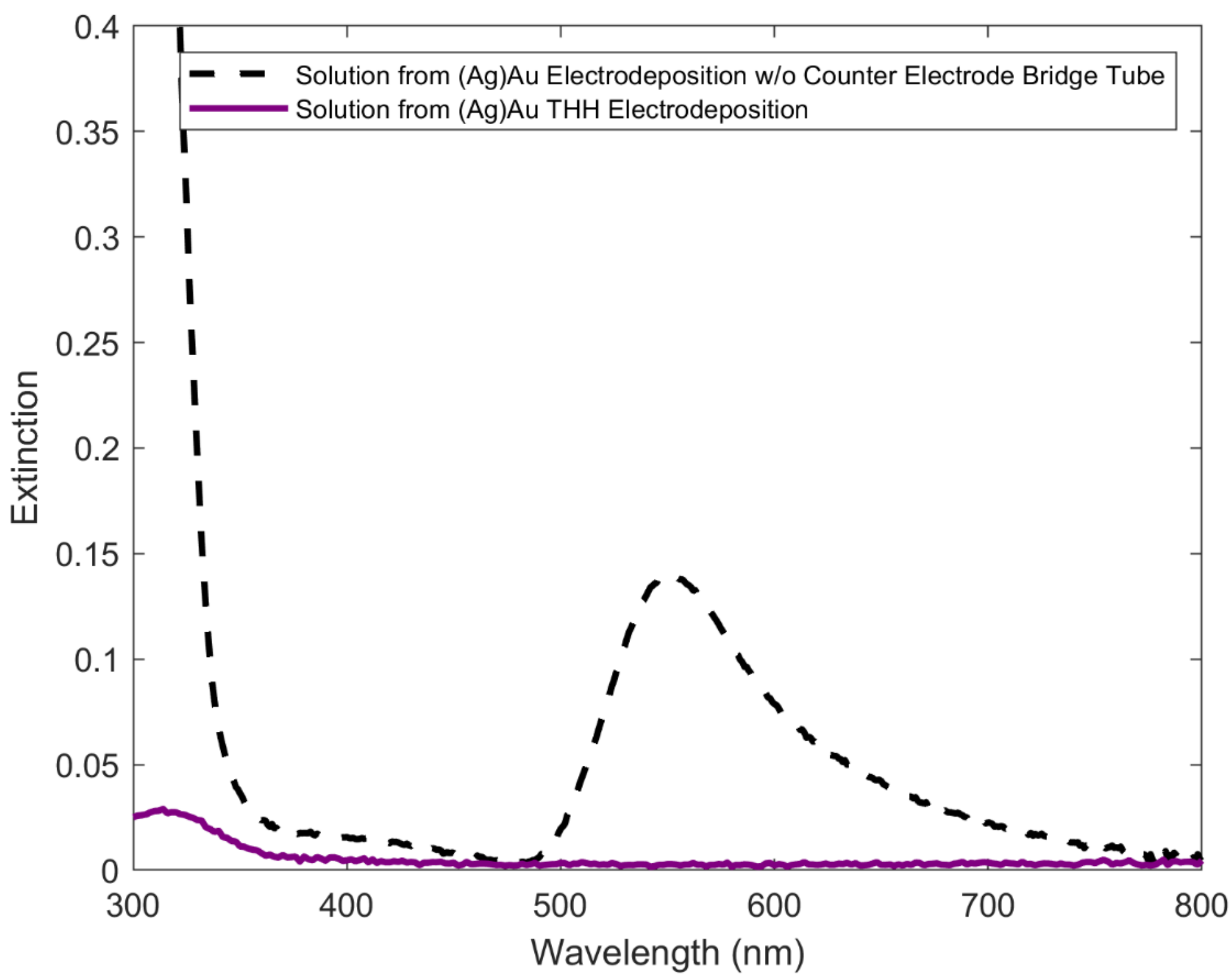

Figure S34. UV-vis spectra of growth solutions following electrodeposition of (Ag)Au THH nanoparticles (-0.713 μA/cm^2 applied for 60 minutes; CTAB; 40 °C), showing a lack of solution phase nanoparticle growth. A representative growth solution following an electrodeposition synthesis of (Ag)Au nanoparticles (-0.815 μA/cm^2 applied for 60 minutes; CTAC; room temperature) with a Pt wire counter electrode inserted directly into solution, in which solution-phase growth of (Ag)Au nanoparticles occurs due to chemical reduction, is provided for comparison.

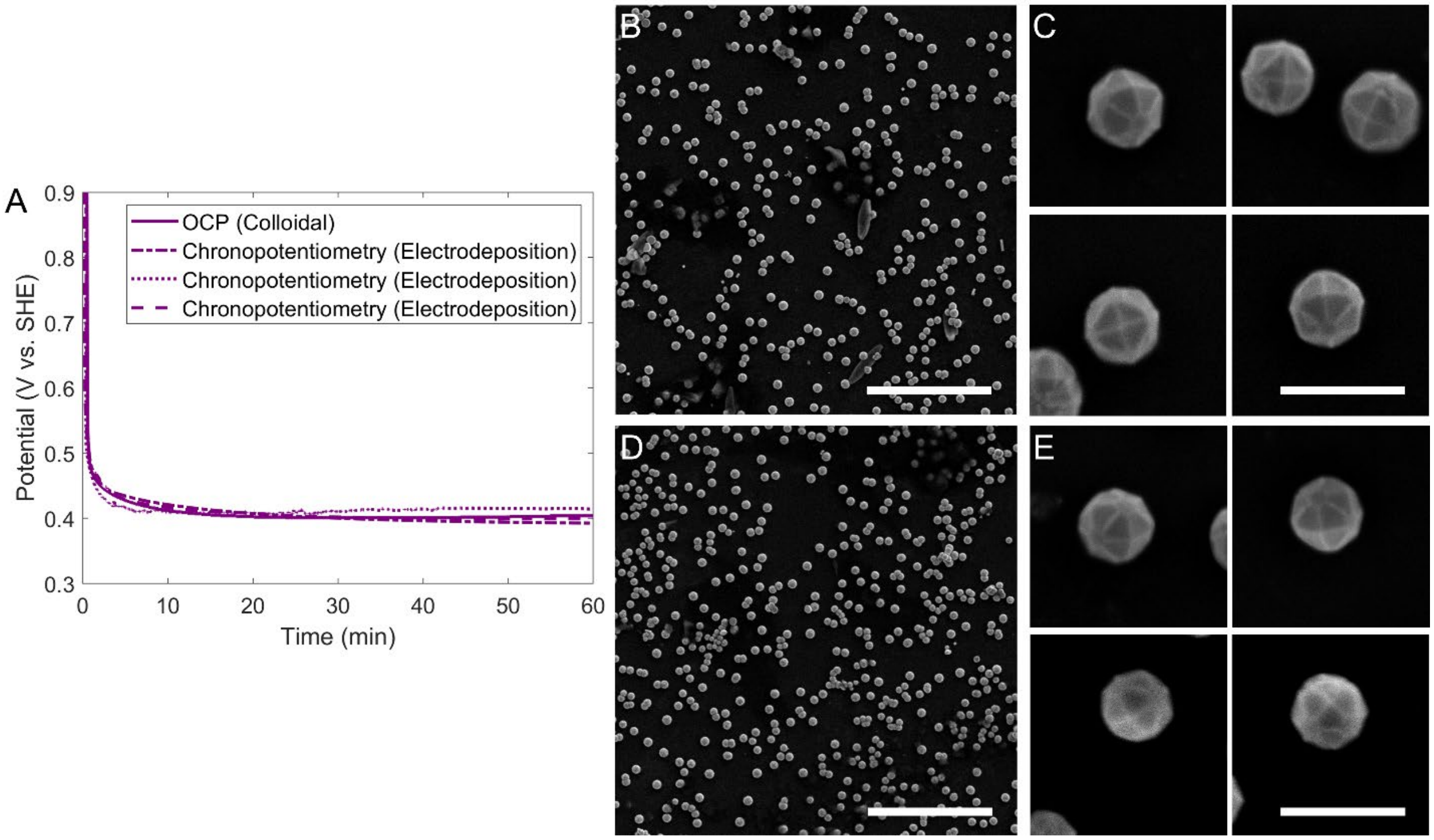

Figure S35. (A) Comparison between OCP measurement of colloidal THH growth and triplicate chronopotentiometry measurements of galvanostatic electrodeposition of THH in 115 mM CTAB with 2.3 µM Ag^+ and -0.713 µA/cm^2 applied current for 60 minutes, onto 1000-fold diluted CTAB-capped Au seeds. (B-E) Representative SEM images of electrodeposited THH nanoparticles from triplicate electrodeposition experiments, showing (B, D) deposition density and (C, E) high-magnification images of individual THH particles. See Figure 5 for SEM images from the first of the triplicate electrodeposition experiments. Scale bars: 5 µm (B, D) and 500 nm (C, E).

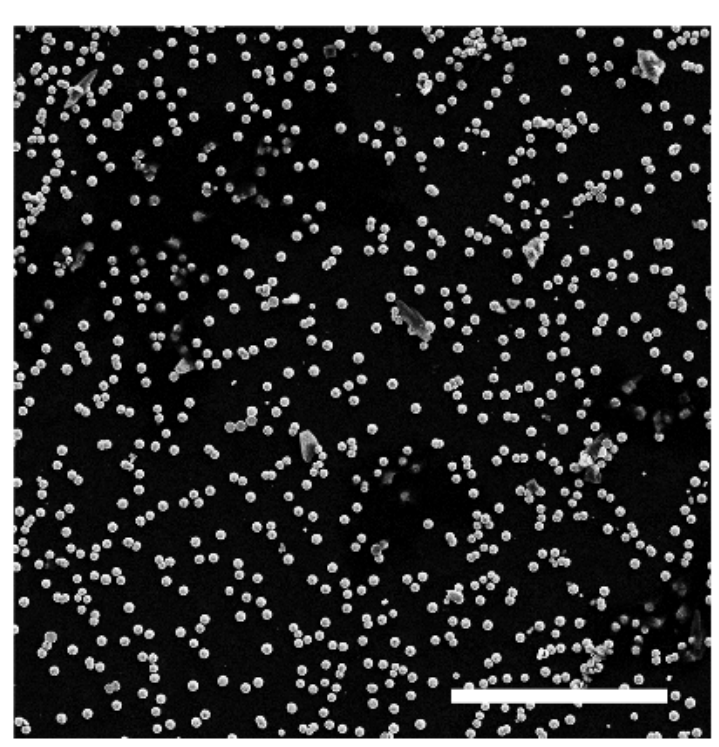

Figure S36. Representative low-magnification SEM image used to determine particle surface density (11±4 particles per µm^2) of electrodeposited (Ag)Au THH deposited with 1000-fold dilution of the seed colloid. Scale bars: 10 µm.

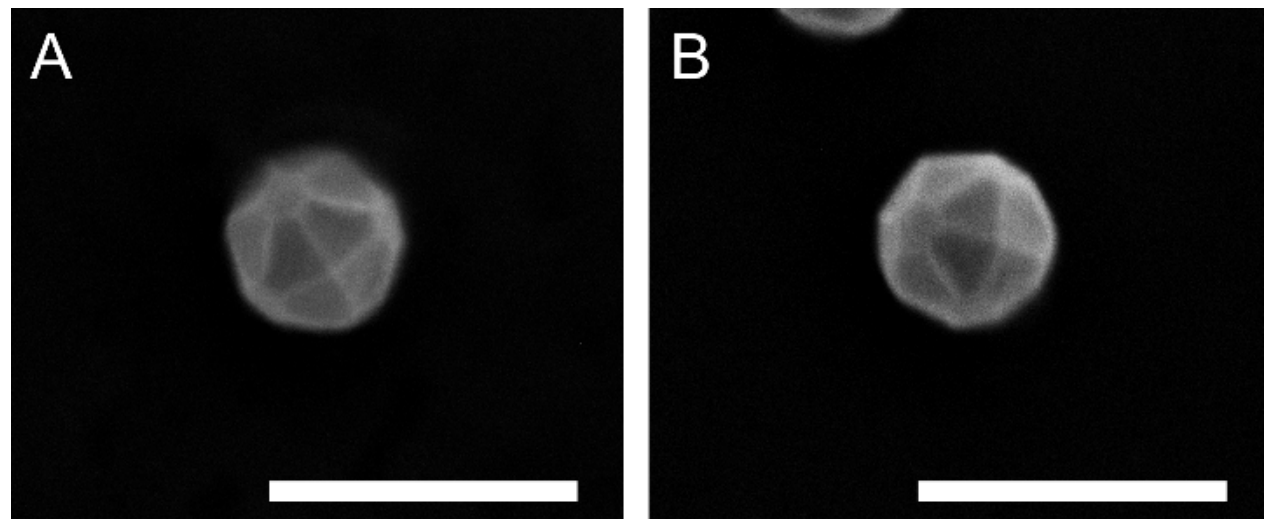

Figure S37. (A-B) Additional representative SEM images of electrodeposited (Ag)Au THH nanoparticles, showing corner truncations. Scale bars: 500 nm.

Table S5. Experimental Conditions for Electrodeposition of Shaped (Ag)Au Nanoparticles.

**Nanoparticle Shape**	**Dilution Factor for Dropcast Au Seed Colloid**	**[CTAC] or [CTAB]**	**[$AgNO_3$]**	**Current Density**
(Ag)Au CC; high seed density	200-fold dilution	10 mL of 125 mM CTAC	23 µL of 10 mM $AgNO_3$	-0.815 $\mu A/cm^2$
(Ag)Au CC; low seed density	500-fold dilution	10 mL of 125 mM CTAC	33 µL of 10 mM $AgNO_3$	-0.815 $\mu A/cm^2$
(Ag)Au CC and side products; highest seed density	10-fold dilution	10 mL of 125 mM CTAC	10 µL of 10 mM $AgNO_3$	-0.815 $\mu A/cm^2$
(Ag)Au octahedra; high seed density	200-fold dilution	10 mL of 125 mM CTAC	1 µL of 2.5 mM $AgNO_3$	-0.815 $\mu A/cm^2$
(Ag)Au octahedra; low seed density	500-fold dilution	10 mL of 125 mM CTAC	1 µL of 3.6 mM $AgNO_3$	-0.739 $\mu A/cm^2$
(Ag)Au RD; high seed density	200-fold dilution	10 mL of 140 mM CTAC	2.5 µL of 10 mM $AgNO_3$	-0.713 $\mu A/cm^2$
(Ag)Au RD; low seed density	500-fold dilution	10 mL of 140 mM CTAC	3.6 µL of 10 mM $AgNO_3$	-0.713 $\mu A/cm^2$
(Ag)Au TDP; high seed density	200-fold dilution	10 mL of 130 mM CTAC	10 µL of 10 mM $AgNO_3$	-0.739 $\mu A/cm^2$
(Ag)Au TDP; low seed density	500-fold dilution	10 mL of 130 mM CTAC	17 µL of 10 mM $AgNO_3$	-0.739 $\mu A/cm^2$
(Ag)Au THH	500-fold dilution	10 mL of 125 mM CTAC	1.4 µL of 10 mM $AgNO_3$	-0.866 $\mu A/cm^2$
(Ag)Au THH	1000-fold dilution	10 mL of 115 mM CTAC	2.5 µL of 10 mM $AgNO_3$	-0.713 $\mu A/cm^2$

References

(1) Pattadar, D. K.; Masitas, R. A.; Stachurski, C. D.; Cliffel, D. E.; Zamborini, F. P. Reversing the Thermodynamics of Galvanic Replacement Reactions by Decreasing the Size of Gold Nanoparticles. *J. Am. Chem. Soc.* **2020**, *142*, 19268–19277. https://doi.org/10.1021/jacs.0c09426

(2) Kang, H.; Kim, B.-G.; Na, H. B.; Hwang, S. Anti-Galvanic Reduction of Silver Ion on Gold and Its Role in Anisotropic Growth of Gold Nanomaterials. *J. Phys. Chem. C* **2015**, *119*, 25974–25982. https://doi.org/10.1021/acs.jpcc.5b08216

(3) Toh, H. S.; Batchelor-McAuley, C.; Tschulik, K.; Uhlemann, M.; Crossley, A.; Compton, R. G. The Anodic Stripping Voltammetry of Nanoparticles: Electrochemical Evidence for the Surface Agglomeration of Silver Nanoparticles. *Nanoscale* **2013**, *5*, 4884. https://doi.org/10.1039/c3nr00898c

(4) Jacobs, E. S. Anodic Stripping Voltammetry of Gold and Silver with Carbon Paste Electrodes. *Anal. Chem.* **1963**, *35*, 2112–2115. https://doi.org/10.1021/ac60206a037

(5) Henglein, A. Physicochemical Properties of Small Metal Particles in Solution: “Microelectrode” Reactions, Chemisorption, Composite Metal Particles, and the Atom-to-Metal Transition. *J. Phys. Chem.* **1993**, *97*, 5457–5471. https://doi.org/10.1021/j100123a004

(6) Plieth, W. J. Electrochemical Properties of Small Clusters of Metal Atoms and Their Role in the Surface Enhanced Raman Scattering. *J. Phys. Chem.* **1982**, *86*, 3166–3170. https://doi.org/10.1021/j100213a020

(7) Ivanova, O. S.; Zamborini, F. P. Electrochemical Size Discrimination of Gold Nanoparticles Attached to Glass/Indium−Tin-Oxide Electrodes by Oxidation in Bromide-Containing Electrolyte. *Anal. Chem.* **2010**, *82*, 5844–5850. https://doi.org/10.1021/ac101021q

(8) Sagberg, P.; Lund, W. Trace Metal Analysis by Anodic-Stripping Voltammetry Effect of Surface-Active Substances. *Talanta* **1982**, *29*, 457–460. https://doi.org/10.1016/0039-9140(82)80194-X

(9) Kubiak, W. W.; Wang, J. Anodic-Stripping Voltammetry of Heavy Metals in the Presence of Organic Surfactants. *Talanta* **1989**, *36*, 821–824. https://doi.org/10.1016/0039-9140(89)80161-4

(10) Sharma, J. N.; Pattadar, D. K.; Mainali, B. P.; Zamborini, F. P. Size Determination of Metal Nanoparticles Based onElectrochemically Measured Surface-Area-to-Volume Ratios. *Anal. Chem.* **2018**, *90*, 9308–9314. https://doi.org/10.1021/acs.analchem.8b01905

(11) Personick, M. L.; Langille, M. R.; Zhang, J.; Mirkin, C. A. Shape Control of Gold Nanoparticles by Silver Underpotential Deposition. *Nano Lett.* **2011**, *11*, 3394–3398. https://doi.org/10.1021/nl201796s

(12) Halford, G. C.; Sublett, A. M.; Personick, M. L. Real-Time Electroanalytical Measurements of Dynamic Reaction Chemistry Provide Generalizable Mechanistic Considerations for Metal Nanoparticle Synthesis. *J. Am. Chem. Soc.* **2026**, *148*, 7280–7299. https://doi.org/10.1021/jacs.5c19321.
